# Azolium Control of the Osmium-Promoted Aromatic C–H Bond Activation in 1,3-Disubstituted Substrates

**DOI:** 10.1021/acs.organomet.1c00565

**Published:** 2021-11-18

**Authors:** Lara Cancela, Miguel A. Esteruelas, Montserrat Oliván, Enrique Oñate

**Affiliations:** Departamento de Química Inorgánica-Instituto de Síntesis Química y Catálisis Homogénea (ISQCH)-Centro de Innovación en Química Avanzada (ORFEO-CINQA), Universidad de Zaragoza-CSIC, 50009 Zaragoza, Spain

## Abstract

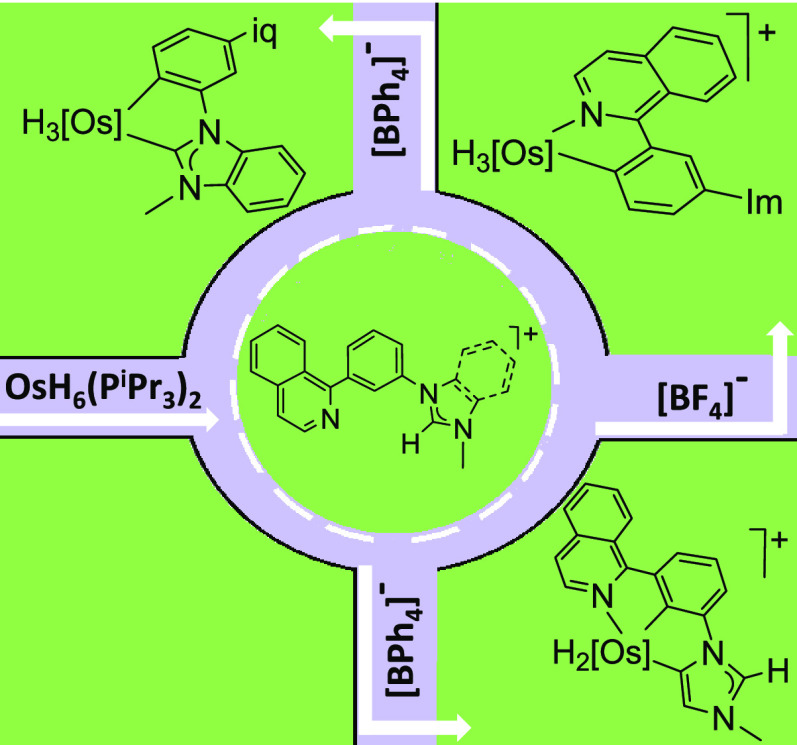

The hexahydride complex OsH_6_(P^i^Pr_3_)_2_ promotes the C–H bond activation of the 1,3-disubstituted phenyl group of the [BF_4_]^−^ and [BPh_4_]^−^ salts of the cations 1-(3-(isoquinolin-1-yl)phenyl)-3-methylimidazolium and 1-(3-(isoquinolin-1-yl)phenyl)-3-methylbenzimidazolium. The reactions selectively afford neutral and cationic trihydride-osmium(IV) derivatives bearing κ^2^-*C,N*- or κ^2^-*C,C*-chelating ligands, a cationic dihydride-osmium(IV) complex stabilized by a κ^3^-*C,C,N*-pincer group, and a bimetallic hexahydride formed by two trihydride-osmium(IV) fragments. The metal centers of the hexahydride are separated by a bridging ligand, composed of κ^2^-*C,N*- and κ^2^-*C,C*-chelating moieties, which allows electronic communication between the metal centers. The wide variety of obtained compounds and the high selectivity observed in their formation is a consequence of the main role of the azolium group during the activation and of the existence of significant differences in behavior between the azolium groups. The azolium role is governed by the anion of the salt, whereas the azolium behavior depends upon its imidazolium or benzimidazolium nature. While [BF_4_]^−^ inhibits the azolium reactions, [BPh_4_]^−^ favors the azolium participation in the activation process. In contrast to benzimidazolylidene, the imidazolylidene resulting from the deprotonation of the imidazolium substituent coordinates in an abnormal fashion to direct the phenyl C–H bond activation to the 2-position. The hydride ligands of the cationic dihydride-osmium(IV) pincer complex display intense quantum mechanical exchange coupling. Furthermore, this salt is a red phosphorescent emitter upon photoexcitation and displays a noticeable catalytic activity for the dehydrogenation of 1-phenylethanol to acetophenone and of 1,2-phenylenedimethanol to 1-isobenzofuranone. The bimetallic hexahydride shows catalytic synergism between the metals, in the dehydrogenation of 1,2,3,4-tetrahydroisoquinoline and alcohols.

## Introduction

The transition-metal-promoted activation of aromatic C–H bonds is one of the most relevant reactions in current chemistry,^1^ due to the wide range of fields with which it is connected, ranging from organic^2^ and organometallic^3^ synthesis to catalysis^4^ and materials science.^5^ The reaction is initiated by the coordination of the C–H bond to the unsaturated metal center of the promoter.^6^ The resulting σ-intermediate evolves by oxidative addition of the C–H bond or heterolytic C–H splitting. In the last case, the abstractor of the proton is a ligand of the metal coordination sphere or an external base.^7^ In accordance with this sequence of events, the activation energy for the C–H bond rupture depends upon two factors: the stability of the σ-intermediate and the C–H bond dissociation energy of the coordinated bond.^8^ Because in aromatic organic molecules the strengths of the different C(sp^2^)–H bonds are similar, the activation is mainly governed by the stability of the σ-intermediate, which is a function of the steric hindrance experienced by the coordinated C–H bond. As a consequence, the selectivity of C(sp^2^)–H bond activation in substituted aromatic arenes is kinetically controlled by steric factors.^9^

The presence of a substituent with coordinating ability in the arene selectively ties the activation at the *ortho* position.^10^ Although the latter is sterically hindered and therefore the last position being activated, the substituent thermodynamically abducts the *ortho*-activation product by coordination.^11^ This is of central importance for the comprehension of catalytic organic reactions of *ortho*-CH functionalization.^12^ Since a catalytic cycle represents the reaction pathway with the lowest activation energy and the *ortho*-metalation reaction has an activation energy higher than those of other C–H bond activations in the same ring, the *o*-CH bond activation should form part of the fast stage of the functionalization, the *ortho*-metalated intermediate being the resting state of the catalyst. An additional issue of selectivity appears when the arene bears several substituents with coordinating ability. Then, understanding the drivers of the selectivity in the activation process is especially relevant to control the products. In such a case, in addition to the steric hindrance of the C–H bonds, the coordinating ability of the different substituents should be also taken into account. The study of the selectivity is particularly challenging when the substituted arene is a part of an imidazolium salt because the imidazolylidene coordination can take place at different positions^13^ and has proved to be anion dependent.^14^ Furthermore, the necessary imidazolium C–H bond activation requires specific procedures for each case.^15^

**Chart 1:**
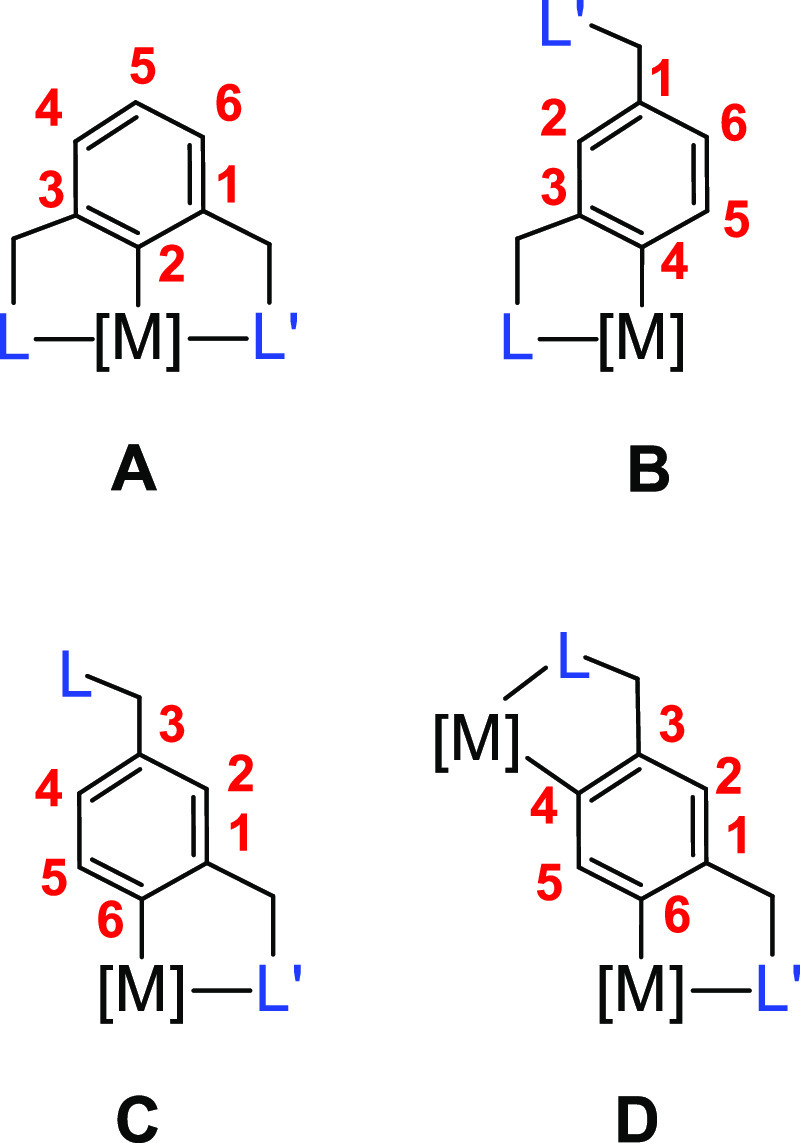
Possible Products of Thermodynamic Control for the C–H Bond Activation of an Aryl Substrate Asymmetrically Substituted with Coordinating Groups

The study of C–H bond activation reactions of aryl substrates asymmetrically 1,3-disubstituted with coordinating groups is particularly challenging. Three different activations can have a thermodynamic preference in this case, which give rise to four distinct stable situations (Chart 1). Activation at the congested 2-position lead to pincer-type derivatives (**A**),^16^ whereas separate activations at positions 4 and 6 provoke κ^2^-*C,L* and κ^2^-*C,L’* coordinations of the activated substrate, which generate mononuclear derivatives bearing C–L and C–L′ chelating ligands (**B** and **C**, respectively).^17^ In contrast, the simultaneous or sequential C–H bond activations of both positions yield a bimetallic species (**D**).^18^ The 5-position is the most accessible. This kinetically favors its activation. However, the absence of a neighboring group with coordinating ability causes such a C–H bond activation to be inhibited from a thermodynamic point of view.

**Chart 2:**
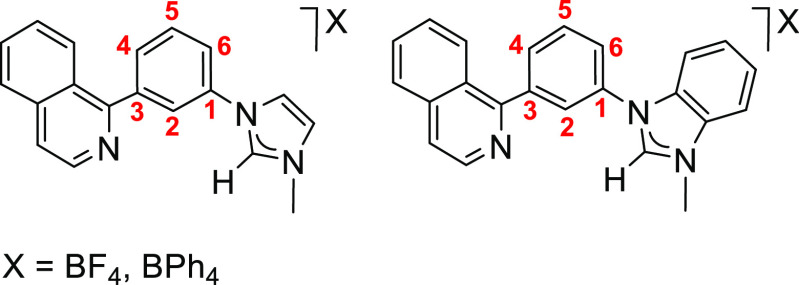
Azolium Salts Used in This Study

The chemistry of the polyhydrides of platinum-group metals is an area of great potential. Such a prospect is the consequence of the proven ability of these compounds to activate σ-bonds,^19^ which allows them to connect with fields such as organic synthesis,^20^ the preparation of new types of phosphorescent emitters for OLED devices,^14e,21^ and hydrogen storage and transport.^22^ Among the compounds of this class, the osmium-hexahydride OsH_6_(P^i^Pr_3_)_2_^23^ (**1**) occupies a prominent position due to its versatility for promoting C–H bond activation reactions.^24^ We are not strangers to the interest in the polyhydride chemistry nor to the aromatic C–H bond activations of substrates asymmetrically 1,3-disubstituted. Thus, in the search for understanding the factors that govern the challenging selectivity of these reactions, we have investigated the behavior of the [BF_4_]^−^ and [BPh_4_]^−^ salts of cations 1-(3-(isoquinolin-1-yl)phenyl)-3-methylimidazolium and 1-(3-(isoquinolin-1-yl)phenyl)-3-methylbenzimidazolium (Chart 2) toward **1**.

This paper describes the selectivity of the osmium-promoted C–H bond activation of the salts shown in Chart 2, as a function of the anion and the azolium substituent and the catalytic performance of the isolated complexes for hydrogen generation by dehydrogenation of 1,2,3,4-tetrahydroisoquinoline and alcohols.

## Results and Discussion

### Complexes Resulting from [BF_4_]^−^ and [BPh_4_]^−^ Salts of 1-(3-(Isoquinolin-1-yl)phenyl)-3-methylimidazolium

The most clean, direct, and straightforward procedure to introduce an imidazolylidene ligand into the coordination sphere of a transition metal is generally direct metalation.^15^ The latter can take place by oxidative addition of an imidazolium C–H bond to an unsaturated metal fragment and by displacement of a coordinated Brønsted base, as a result of its protonation with the imidazolium salt. Complex **1** shows a marked tendency to undergo the reductive elimination of molecular hydrogen, at moderate temperatures (>50 °C), to afford the unsaturated tetrahydride OsH_4_(P^i^Pr_3_)_2_ (**E**), which is the true species responsible for the proved ability of **1** to activate σ-bonds.^11c,20d,20f,24^ On the other hand, the hydrides of **1** are basic enough to promote the deprotonation of imidazolium salts. The addition of the proton initially leads to the known trihydride-bis(dihydrogen) derivative [OsH_3_(η^2^-H_2_)_2_(P^i^Pr_3_)_2_]^+^, which loses molecular hydrogen and dimerizes to form the bimetallic cation [{OsH_2_(P^i^Pr_3_)_2_}_2_(μ-H)_3_]^+^ in equilibrium with the deprotonated polyhydride (P^i^Pr_3_)_2_H_2_Os(μ-H)_3_OsH(P^i^Pr_3_)_2_.^25^ To prevent side products resulting from the formation of the OsH_7_ cation, the reactions of **1** with imidazolium salts are usually performed in the presence of triethylamine, including those where the imidazolylidene ligand acts as a chelating assistant.^26^

**Scheme 1:**
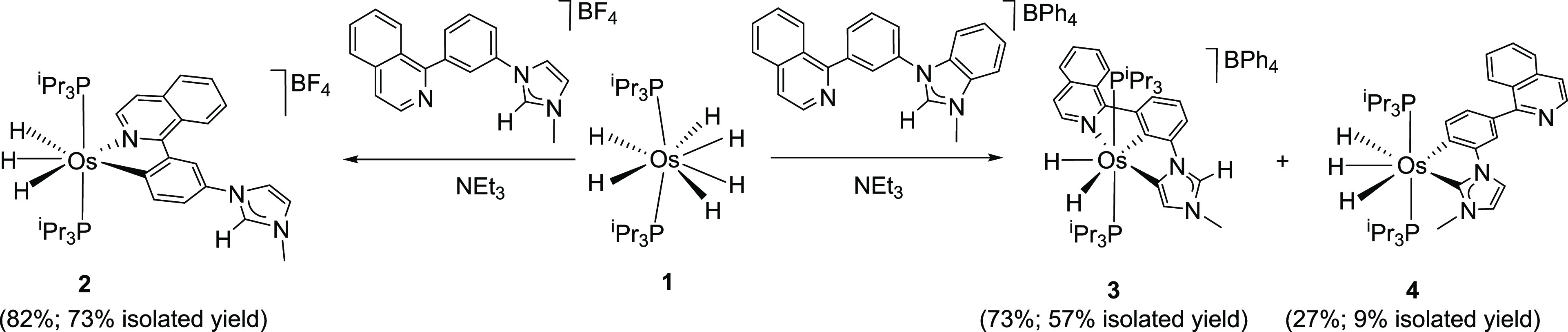
Formation of Complexes **2**–**4**

Treatment of toluene solutions of **1** with 1.0 equiv of the [BF_4_]^−^ salt of 1-(3-(isoquinolin-1-yl)phenyl)-3-methylimidazolium, in the presence of 15 equiv of triethylamine, under reflux leads to the cationic trihydride derivative **2** (Scheme 1) in 82% yield after 24 h, according to the ^1^H and ^31^P{^1^H} NMR spectra of the crude reaction product in dichloromethane-*d*_2_. The reaction can be rationalized as the isoquinolinyl-assisted activation of the C–H bond of the phenyl group at position 4 promoted by tetrahydride **E**. The imidazolium moiety of the salt does not interfere during the process. Consistently, in this case, the formation of the trihydride also takes place in the absence of the amine and in the same extension.

**Figure 1:**
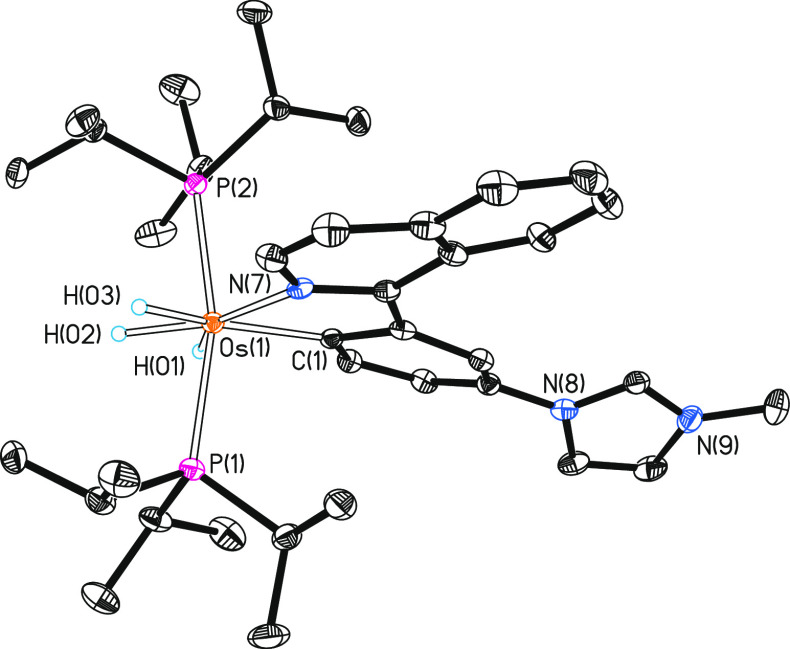
Molecular diagram of one of the two independent cations of complex **2** (ellipsoids shown at 50% probability) in the asymmetric units. All hydrogen atoms (except the hydrides) are omitted for clarity. Selected bond distances (Å) and angles (deg): Os–P(1) = 2.3527(10), 2.3368(11), Os–P(2) = 2.3367(10), 2.3423(11), Os–C(1) = 2.102(4), 2.098(4), Os–N(7) = 2.151(3), 2.151(3); P(1)–Os–P(2) = 164.64(4), 166.91(4), C(1)–Os–N(7) = 75.51(14), 75.06(14).

Complex **2** was isolated as a red solid in 73% yield and characterized by X-ray diffraction analysis. The structure has two cations and two anions chemically equivalent, but these are crystallographically independent in the asymmetric unit. Figure 1 gives a view of a cation. The metal center displays a typical coordination for a *d*^4^ ion. Thus, the polyhedron can be idealized as a pentagonal bipyramid with axial phosphines (P(1)–Os(1)–P(2) = 164.64(4) and 166.91(4)°). The κ^2^-*C,N*-chelate group, which acts with bite angles of 75.51(14) and 75.06(14)° (C(1)–Os(1)–N(7)), and the hydride ligands, which are separated by more than 1.6 Å (X-ray and DFT calculations (B3LYP-D3(SMD)/6-31G**(SDD)), lie at the base. The ^1^H, ^13^C{^1^H}, and ^31^P{^1^H} NMR spectra in dichloromethane-*d*_2_ are consistent with the solid-state structure. In agreement with the presence of three inequivalent hydride ligands, the ^1^H spectrum at 193 K shows three high-field resonances at −6.19, −10.71, and −12.10 ppm. The most noticeable signal in the ^13^C{^1^H} spectrum is a triplet (^2^*J*_C–P_ = 5.8 Hz) at 199.1 ppm, corresponding to the metalated carbon atom. The ^31^P{^1^H} spectrum displays a singlet at 22.1 ppm, as expected for equivalent phosphines.

There are significant differences in behavior between the [BF_4_]^−^ and [BPh_4_]^−^ salts of the cation 1-(3-(isoquinolin-1-yl)phenyl)-3-methylimidazolium. Tentatively, these differences may be associate with the distinct sizes of the anions, which influence the cation–anion association and the respective solvations. In contrast to the [BF_4_]^−^ salt, the [BPh_4_]^−^ counterpart allows the disinhibition of the reactivity of the imidazolium moiety. This favors the activations of the C–H bonds of the phenyl group at the 2- and 6-positions (Scheme 1). Thus, the treatment of a toluene solution of **1** with the [BPh_4_]^−^ salt, in the presence of 15 equiv of triethylamine, under reflux affords a mixture of the cationic dihydride pincer complex **3** (73%) and the neutral trihydride **4** (27%). The major product, complex **3**, results from the activations of C–H bonds of the imidazolium moiety and of the phenyl group at 5- and 2-positions, respectively, whereas the neutral trihydride **4** arises from similar ruptures at 2- and 6-positions of the respective rings. The formation of a pincer isomer of **3** involving the coordination of the carbon atom at the 2-position of the imidazolylidene instead of that at the 5-position was not observed. This suggests that the C–H ruptures leading to **3** are connected and take place in a sequential manner. Because the 2-position of the phenyl group is sterically more hindered than the 5-position of the imidazolium moiety, it seems reasonable to think that the latter is kinetically favored and therefore it is previous to the former. As expected from the imidazolium participation, the reaction is sensitive to triethylamine. In absence of the latter, in addition to the appearance of side products, the formation of **3** and **4** is slower.

**Figure 2:**
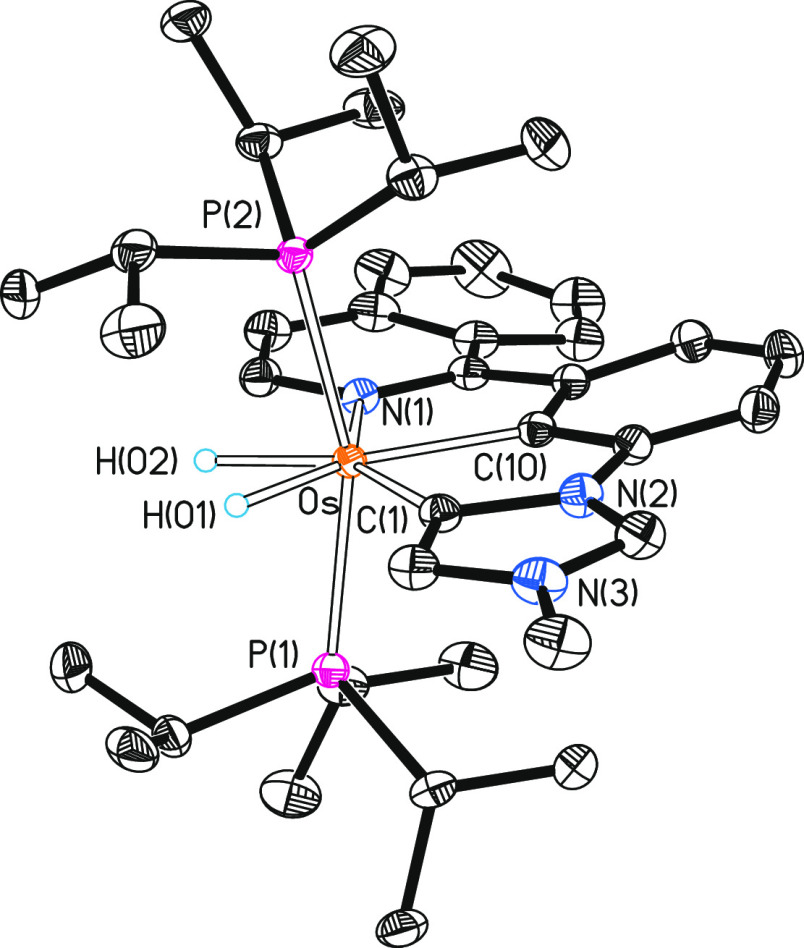
Molecular diagram of the cation of complex **3** (ellipsoids shown at 50% probability). All hydrogen atoms (except the hydrides) are omitted for clarity. Selected bond distances (Å) and angles (deg): Os–P(1) = 2.3714(5), Os–P(2) = 2.3885(5), Os–C(1) = 2.077(2), Os–C(10) = 2.053(2), Os–N(1) = 2.1456(18); P(1)–Os–P(2) = 160.46(2), C(1)–Os–N(1) = 150.42(8), C(1)–Os–C(10) = 76.18(8), C(10)–Os–N(1) = 74.24(8).

Complex **3** was separated from the crude reaction mixture by silica column chromatography, isolated as a red solid in 57% yield, and subsequently fully characterized including an X-ray diffraction analysis. Figure 2 shows a view of the cation. The structure demonstrates the formation of the pincer, involving an abnormal coordination of the imidazolylidene moiety. The osmium–imidazolylidene bond length of 2.077(2) Å (Os–C(1)) compares well with those reported for osmium compounds displaying abnormal-NHC coordination.^11c,27^ The new monoanionic *C,C,N*-pincer ligand acts with C(1)–Os–N(1), C(1)–Os–C(10), and N(1)–Os–C(10) angles of 150.42(8), 76.18(8), and 74.24(8)°, respectively, which are close to the ideal values corresponding to three consecutive positions at the base of a pentagonal bipyramid (144, 72, and 72°), the observed coordination polyhedron in this case, and point out that this pincer should be particularly useful to stabilize compounds of d^4^ ions with such a disposition of donor atoms around the metal center. The ideal pentagonal bipyramid is completed with the phosphines, which lie at the apical positions (P(1)–Os–P(2) = 160.46(2)°), and the hydrides, which are situated at the pincer plane separated by 1.55(4) Å (1.647 Å in the DFT-optimized structure).

**Figure 3:**
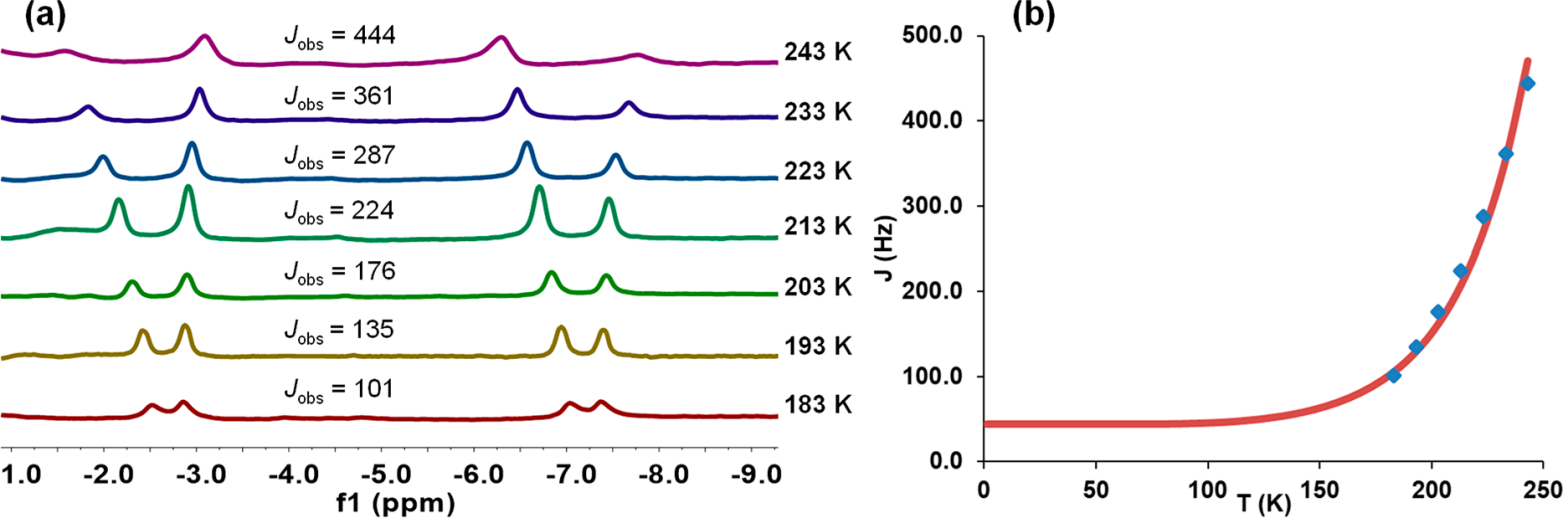
(a) ^1^H{^31^P} NMR spectra of complex **3** in the high-field region as a function of the temperature. (b) Plot of *J*_obs_ versus temperature for complex **3**.

The NMR spectra in dichloromethane-*d*_2_ are consistent with the solid-state structure. The ^1^H spectrum further reveals that the hydride ligands undergo quantum mechanical exchange coupling.^19,28^ As shown in Figure 3a, the observed H–H coupling constant (*J*_obs_) in the AB part of the ABX_2_ (X = ^31^P) spin system corresponding to the dihydride resonance (−5.7 ppm) is temperature (*T*) dependent, increasing from 101 to 444 Hz as *T* increases from 183 to 243 K. For a given hydrogen–hydrogen separation (*a*), *J*_obs_ and *T* are related through eq 1, according to a two-dimensional harmonic oscillator model,^29^ where *J*_mag_ is the classical H–H coupling constant due to the Fermi contact interaction, λ represents the hard sphere radius of the hydrides, and ν describes the H–M–H vibrational wag mode that allows the movement along the H–H vector. As for *a*, these parameters are temperature independent. Using the hydrogen–hydrogen separation obtained by DFT calculations for the optimized structure, the fitting of the plot shown in Figure 3b yields values of *J*_mag_ = 9.5 Hz, λ = 1.0 Å, and ν = 497 cm^–1^, which compare well with those obtained for other osmium(IV)-hydride compounds.^30^ In the ^13^C{^1^H} spectrum, the most noticeable resonances are two triplets at 192.4 (^2^*J*_C–P_ = 5.9 Hz) and 140.2 (^2^*J*_C–P_ = 7.3 Hz) ppm, due to the metalated C(1) and C(10) atoms, respectively. The ^31^P{^1^H} spectrum shows a singlet at 3.4 ppm, in agreement with the equivalence of the phosphines.
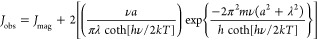
1

**Figure 4:**
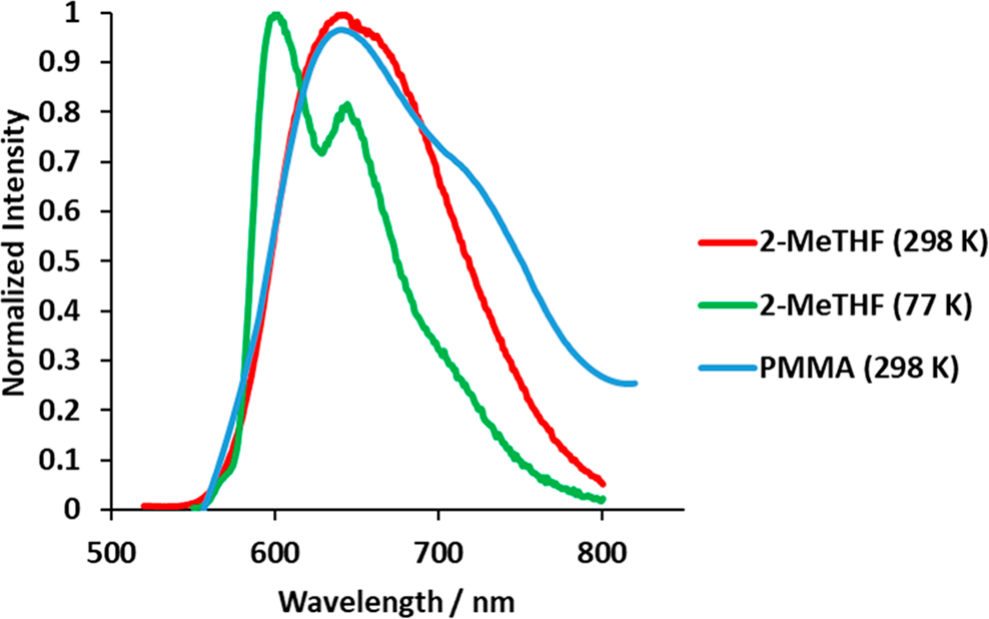
Emission spectra of complex **3**.

Complex **3** is a new case of a red phosphorescent emitter (601–644 nm) upon photoexcitation, in a 5 wt % doped poly(methyl methacrylate) (PMMA) film at room temperature and in 2-methyltetrahydrofuran (2-MeTHF) at room temperature and at 77 K (Table 1). The observed wavelengths are in accordance with those obtained by estimating the difference in energy between the optimized triplet state T_1_ and the singlet state S_0_ in THF (637 nm). Consistently, the emissions can be ascribed to this excited state. The emission spectra in the PMMA film and in 2-MeTHF at room temperature show broad structureless bands. In contrast, the spectrum in 2-MeTHF at 77 K displays a vibronic fine structure (Figure 4), which is consistent with a significant contribution of ligand-centered ^3^π–π* transitions to the excited state.^31^ The lifetimes are short and lie in a narrow range of 0.8–5.2 μs, whereas the quantum yields of about 0.20 are moderate. There is great interest in osmium(IV) emitters. In addition to being more difficult to oxidize than osmium(II) emitters, they should offer more flexibility for color tuning.^32^

**Table 1:** Emission Properties of Complex **3**

HOMO^calc^ (eV)	LUMO^calc^ (eV)	HLG^calc^ (eV)	HOMO^exp^ (eV)^a^	LUMO^exp^ (eV)^b^	calc λ_em_ (nm)^c^	medium (*T*, K)	λ_em_ (nm)	τ_obs_ (μs)	Φ	*k*_r_ (s^–1^)^d^	*k*_nr_ (s^–1^)^d^	*k*_r_/*k*_nr_
						PMMA (298)	644	0.8	0.15	1.8 × 10^5^	1.0 × 10^6^	0.18
–5.23	–2.07	3.16	–5.09	–2.95	637	MeTHF (298)	642	1.1	0.19	1.7 × 10^5^	7.3 × 10^5^	0.23
						MeTHF (77)	601	5.2				

a HOMO = −[*E*^ox^ vs Fc/Fc^+^ + 4.8] eV.

b LUMO = −[*E*^red^ vs Fc/Fc^+^ + 4.8] eV.

c Predicted from TD-DFT calculations in THF at 298 K by estimating the energy difference between the optimized T_1_ and singlet S_0_ states.

d Calculated according to the equations *k*_r_ = Φ/τ_obs_ and *k*_nr_ = (1 – Φ)/τ_obs_, where *k*_r_ is the radiative rate constant, *k*_nr_ is the nonradiative rate constant, Φ is the quantum yield, and τ_obs_ is the excited-state lifetime.

**Figure 5:**
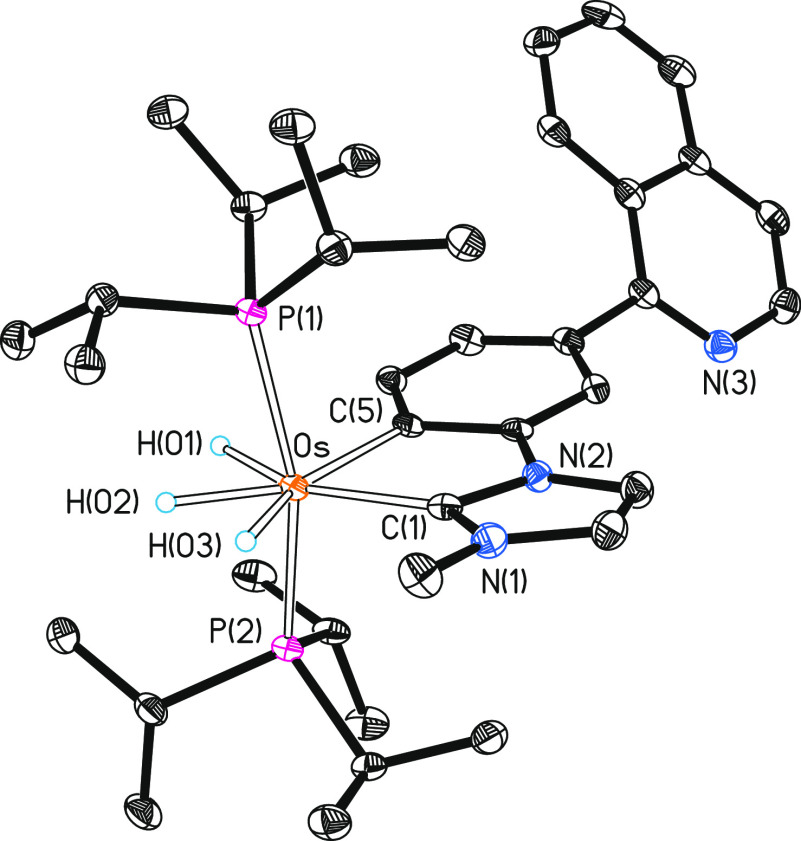
Molecular diagram of complex **4** (ellipsoids shown at 50% probability). All hydrogen atoms (except the hydrides) are omitted for clarity. Selected bond distances (Å) and angles (deg): Os–P(1) = 2.3481(8), Os–P(2) = 2.3511(8), Os–C(1) = 2.065(3), Os–C(5) = 2.128(3); P(1)–Os–P(2) = 163.47(3), C(1)–Os–C(5) = 75.83(12).

The minor complex **4** was isolated as a yellow solid in 9% yield and characterized by X-ray diffraction analysis. Its structure (Figure 5) confirmed the molecular nature of the species and the coordination of the imidazolylidene moiety by the carbon atom at the 2-position of the ring. The polyhedron around the osmium atom resembles that of **2**, with a P(1)–Os–P(2) angle of 163.47(3)°, the hydride ligands separated by more than 1.6 Å, and the imidazolylidene moiety, which forms a κ^2^-*C,C*-chelate with the *ortho*-metalated phenyl (C(1)–Os–C(5) = 75.83(12)°), occupying the isoquinolinyl group place. This ligand disposition is consistent with the NMR spectra of the molecule in dichloromethane-*d*_2_. In accordance with **2**, the ^1^H spectrum at 183 K shows three hydride resonances at −8.90, −10.38, and −10.78 ppm. In the ^13^C{^1^H} spectrum, the signals due to the metalated carbon atoms appear at 190.1 (C(1)) and 162.8 (C(5)) ppm, as triplets with C–P coupling constants of 5.9 and 7.6 Hz, respectively. The ^31^P{^1^H} spectrum contains a singlet at 24.7 ppm for the equivalent phosphines.

### Complexes Resulting from [BF_4_]^−^ and [BPh_4_]^−^ Salts of 1-(3-(Isoquinolin-1-yl)phenyl)-3-methylbenzimidazolium

**Scheme 2:**
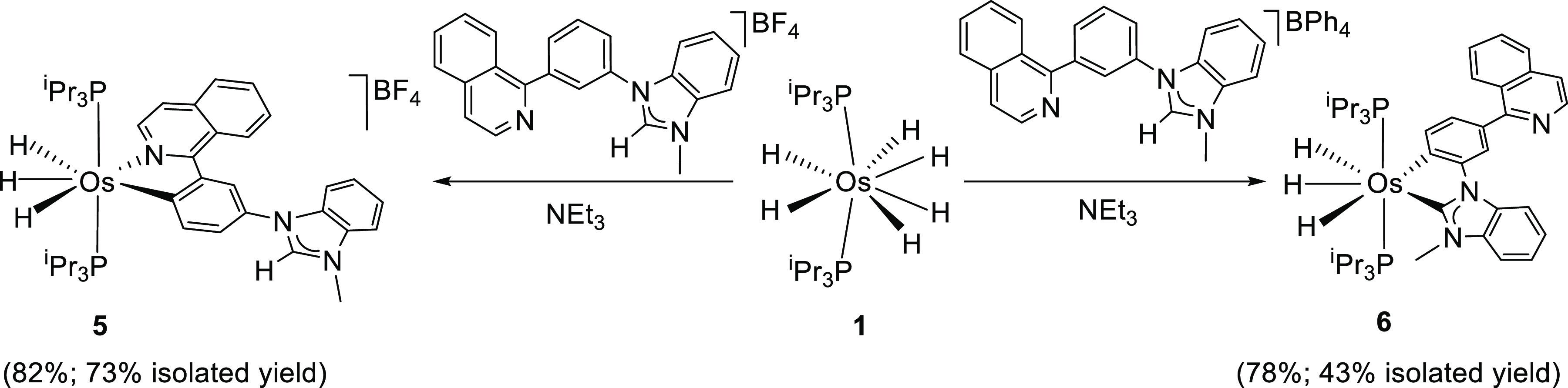
Formation of Complexes **5** and **6**

The use of a benzimidazolium fragment instead of an imidazolium moiety should prevent the formation of a pincer species related to **3**, confirming the linkage between the activation of the C–H bonds at the 5-position of the five-membered ring and the activation of the C–H bond at the 2-position of the phenyl group, while it would allow a better study of the C–H bond activation at the 6-position of the aryl group. This reasoning prompted us to study the reactions of **1** with the [BF_4_]^−^ and [BPh_4_]^−^ salts of the cation 1-(3-(isoquinolin-1-yl)phenyl)-3-methylbenzimidazolium, under the same conditions as those employed for the reactions summarized in Scheme 1. The results are consistent with those obtained for the cation 1-(3-(isoquinolin-1-yl)phenyl)-3-methylimidazolium and confirm our previous conclusions (Scheme 2).

The [BF_4_]^−^ anion inhibits the reactions of the benzimidazolium fragment, which favors the isoquinolinyl-assisted activation of the C–H bond of the phenyl group at the 4-position. Thus, the reaction of **1** with this salt leads to **5** (82%), the benzimidazolylidene counterpart of **2**, while the reaction with the [BPh_4_]^−^ salt selectively gives **6** (78%), the benzimidazolylidene counterpart of **4**. Complex **6** results from the activation of the C–H bond at the 6-position of the phenyl group along with the activation of the C–H bond at the 2-position of the benzimidazolium fragment. No pincer complex resulting from C–H bond activation of the phenyl group at the 2-position was detected, which suggests that the rupture of the C–H bond of the phenyl group at the 6-position is a NHC-assisted reaction promoted by the tetrahydride **E**, the genesis of the Os–NHC bond being a heterolytic C–H activation mediated by the triethylamine external base. Complexes **5** and **6** were isolated as red and yellow solids in 73% and 43% yields, respectively, and fully characterized by NMR spectroscopy, in dichloromethane-*d*_2_. In agreement with the imidazolylidene counterparts **2** and **4**, the ^1^H spectra at 203 K contain signals due to three inequivalent hydrides at −6.19, −10.54, and −11.99 ppm for **5** and at −8.43 and −10.02 (2H) ppm for **6**. In the ^13^C{^1^H} spectra, the resonances corresponding to the metalated carbon atoms appear as triplets at 199.7 (^2^*J*_C–P_ = 5.5 Hz) ppm for **5** and at 206.1 (^2^*J*_C–P_ = 5.8 Hz) and 161.9 (^2^*J*_C–P_ = 5.6 Hz) ppm for **6**. The ^31^P{^1^H} spectra display a singlet at 21.9 ppm for **5** and at 26.1 ppm for **6**.

### C–H Bond Activation of **6**

**Scheme 3:**
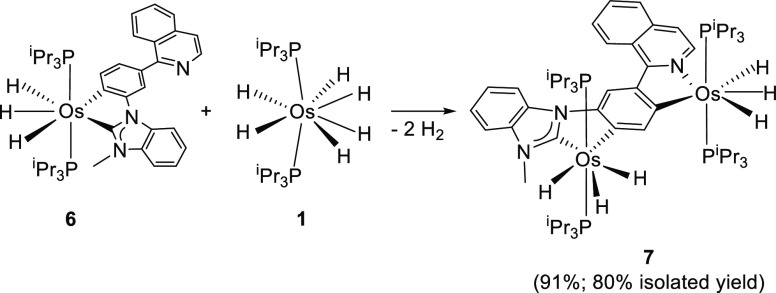
Formation of Complex **7**

The hexahydride complex **1** also activates the C–H bond of the metalated phenyl group of **6** disposed in *para* position with regard to the benzimidazolylidene moiety and *ortho* to the isoquinolyl group, the 4-position in the starting cation, to give the bimetallic hexahydride **7** (Scheme 3). At first glance, one should expect that such a complex could be also prepared from **5**, by activation of the C–H bond at 2-position of the benzimidazolium fragment along with the *ortho* metalation of the phenyl group: i.e., the activation of the C–H bond of the phenyl group disposed in a *para* position with respect to the isoquinolyl moiety, the 6-position of the original cation. However, the previously mentioned inhibition of the reactivity of the benzimidazolium moiety by the action of the [BF_4_]^−^ anion prevents such a possibility, in the presence and in absence of triethylamine and in both toluene and tetrahydrofuran, as solvents, under reflux.

**Figure 6:**
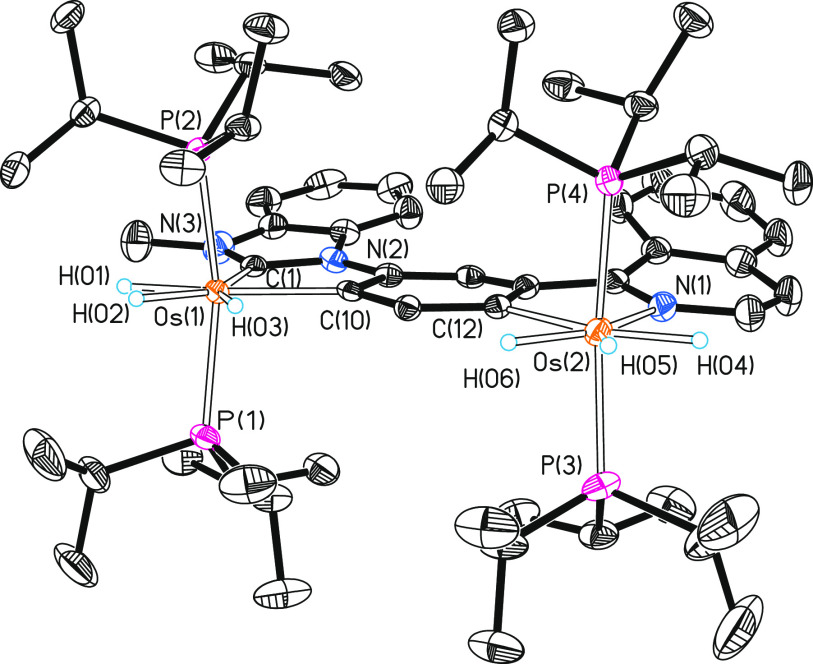
Molecular diagram of complex **7** (ellipsoids shown at 50% probability). All hydrogen atoms (except the hydrides) are omitted for clarity. Selected bond distances (Å) and angles (deg): Os(1)–P(1) = 2.3411(9), Os(1)–P(2) = 2.3462(8), Os(2)–P(3) = 2.3319(9), Os(2)–P(4) = 2.3383(9), Os(1)–C(1) = 2.064(3), Os(1)–C(10) = 2.134(3), Os(2)–C(12) = 2.125(3), Os(2)–N(1) = 2.129(3); P(1)–Os(1)–P(2) = 160.57(3), P(3)–Os(2)–P(4) = 164.30(3); C(1)–Os(1)–C(10) = 76.19(12), C(12)–Os(2)–N(1) = 76.20(12).

Complex **7** was isolated as a garnet solid in 80% yield and characterized by an X-ray diffraction analysis. Figure 6 shows its structure, which can be described as two OsH_3_(P^i^Pr_3_)_2_ metal fragments linked by a bridging ligand resulting from activations at the 4- and 6-positions of a phenyl substrate asymmetrically 1,3-disubstituted with benzimidazolylidene and isoquinolyl groups. The polyhedron around Os(1) resembles that of **6** with P(1)–Os(1)–P(2) and C(1)–Os(1)–C(10) angles of 160.57(3) and 76.19(12)°, respectively, whereas the polyhedron around Os(2) resembles that of **5** with P(3)–Os(2)–P(4) and N(1)–Os(2)–C(12) angles of 164.30(3) and 76.20(12)°, respectively. The classical nature of the polyhydride is supported by both the X-ray structure and the optimized structure through DFT calculations, which display hydride–hydride separations longer than 1.6 Å. In agreement with the structure, the NMR spectra in dichloromethane-*d*_2_ are combinations of those of **5** and **6**. The ^1^H spectrum at 203 K contains high-field signals for six inequivalent hydrides at −6.23, −8.05, −10.05 (2H), and −11.04 (2H) ppm. The ^13^C{^1^H} spectrum shows three triplets (^2^*J*_C–P_ = 6.1–5.7 Hz) for the metalated carbon atoms at 204.9, 186.5, and 166.6 ppm. The two pairs of equivalent phosphines give rise to two singlets at 26.7 and 22.6 ppm in the ^31^P{^1^H} spectrum.

The HOMO of the bimetallic complex **7** is delocalized between the metal centers and the bridge (Figure S38). Bimetallic complexes displaying frontier orbitals delocalized between the two metal centers connected by a π-linker can be viewed as being electronically coupled. Thus, one should expect that changes in the electron density at one site would perturb the electron density at the other.^33^ The redox potential separation between successive redox processes is frequently used as a first evaluation of the electronic coupling.^34^ To analyze this possibility in **7**, we evaluated its redox properties. A cyclic voltammetry experiment was carried out under argon, in dichloromethane solution, with [Bu_4_N]PF_6_ as the supporting electrolyte (0.1 M). Four oxidation peaks at −0.60 ([Os_2_]/[Os_2_]^+^), −0.26 ([Os_2_]^+^/[Os_2_]^2+^), 0.01 ([Os_2_]^2+^/[Os_2_]^3+^), and 0.27 ([Os_2_]^3+^/[Os_2_]^4+^) V versus Fc/Fc^+^ were observed (Figure S39). The first oxidation is reversible, whereas the second and third oxidations are quasi-reversible and the fourth oxidation is irreversible. Reduction peaks were not observed in the range from −1.5 to +1.5 V. The consecutive separations between the three first oxidation peaks (Δ*E*) yield large values for the equilibrium constant *K*_c_ (*K*_c_ = *e*^–*nF*Δ*E*/*RT*^)^35^ of the comproportionation reactions summarized by eq 2, of 5.6 × 10^–5^ and 3.7 × 10^–4^. These values point out the formation of class III radicals, with the odd electron being fully delocalized, according to the Robin–Day classification.^36^
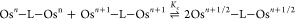
2

The formation of mixed valence species was confirmed by an UV–vis–NIR spectroelectrochemical investigation on a 1 × 10^–3^ M dichloromethane solution of **7**, in the presence of 0.1 M [Bu_4_N]PF_6_, under argon (Figures S50–S54). In agreement with the formation of species of this class, the spectra of [**7**]^+^ and [**7**]^3+^ contain broad absorptions centered at 927 and 1556 nm, respectively, which are ascribed to the respective intervalence charge transfer transitions (IVCTs). In accordance with the bandwidth at the half-height (Δν_1/2_ = 12500 cm^–1^ for [**7**]^+^ and 6436 cm^–1^ for [**7**]^3+^) and the maximum absorption (Δν_max_ = 657 cm^–1^ for [**7**]^+^ and 641 cm^–1^ for [**7**]^3+^) of the Gaussian-shaped ICTV band, the delocalization parameters Γ calculated according to eq 3(36b) are 0.87 for [**7**]^+^ and 0.83 for [**7**]^3+^. These values are characteristic of class III radicals.^37^
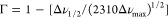
3

### Catalytic Dehydrogenation of 1,2,3,4-Tetrahydroisoquinoline and Alcohols

**Scheme 4:**
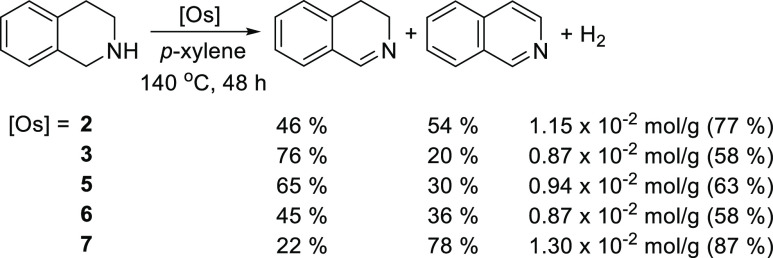
Dehydrogenation of 1,2,3,4-Tetrahydroisoquinoline

The mononuclear complexes **2**, **3**, **5**, and **6** and the bimetallic derivative **7** promote the dehydrogenation of 1,2,3,4-tetrahydroisoquinoline (Scheme 4). The reactions were carried out under argon, in *p*-xylene, at 140 °C, using a heterocycle concentration of 0.12 M and an osmium/heterocycle molar ratio of 1/14.6. Under these conditions, between 58% and 87% of all H_2_ capacity of the heterocycle, 1.50 × 10^–2^ mol g^–1^, is released after 48 h. The dehydrogenation is sequential, the release of the first hydrogen molecule being faster than the liberation of the second molecule. The behavior the bimetallic complex **7** should be pointed out, which reveals a nice example of catalytic synergism.^38^ This compound is significantly more active than complexes **5** and **6**, the mononuclear units forming it. Although the metal centers are separated by the bridging ligand, the latter allows their electronic coupling, as was previously demonstrated. The catalysis can take place in an independent manner in each metal center, but the events in one metal center affect those in the other. The catalytic synergism is a consequence of the gain in the efficiency of each metal center by the action of its colleague.

**Scheme 5:**
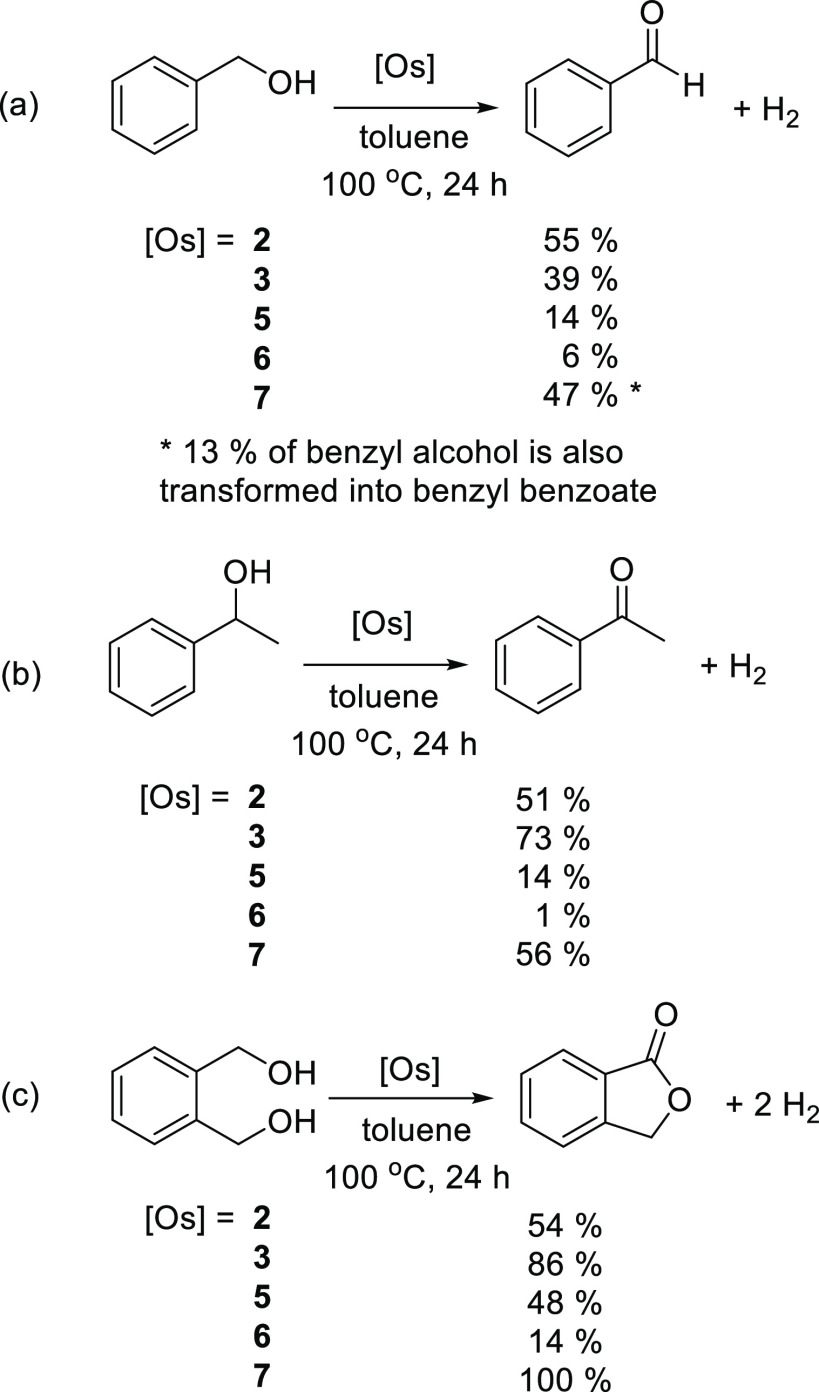
Dehydrogenation of Benzyl Alcohol, 1-Phenylethanol, and 1,2-Phenylenedimethanol

Complexes **2**, **3**, and **5**–**7** also catalyze the dehydrogenation of primary and secondary alcohols, such as benzyl alcohol, 1-phenylethanol, and 1,2-phenylenedimethanol (Scheme 5). The reactions were performed under argon, in toluene, at 100 °C, using an alcohol concentration of 0.12 M and an osmium/substrate molar ratio of 1/14.6. The addition of a base to the catalytic solutions was not necessary. This finding is particularly notable in the case of **2**, **3**, and **5**, given their cationic character.^39^ The hydride ligands of these compounds appear to have enough basic character to deprotonate the OH group of the substrates. The abstraction should release H_2_, generating the key metal-alkoxide intermediates. The organic product obtained and the amount of generated molecular hydrogen depend upon both the nature of the alcohol and the catalyst. The dehydrogenation reactions of benzyl alcohol (Scheme 5a) catalyzed by the mononuclear complexes **2**, **3**, **5**, and **6** afford benzaldehyde in low to moderate yields, 6–55%, after 24 h. However, the alcohol is transformed in a mixture of benzylbenzoate (13%) and benzaldehyde (47%) in the presence of the bimetallic compound **7**. The generation of esters in these reactions is not surprising. They are a consequence of a competitive dehydrogenative homocoupling and seem to result from the transitory formation of hemiacetals.^40^ The dehydrogenation of 1-phenylethanol (Scheme 5b) leads to the expected acetophenone also in moderate yields, with the exception of the pincer salt **3**. The latter generates 73% of the ketone after 24 h. Complex **3** is more efficient than the bimetallic compound **7**. Although the bimetallic species displays catalytic synergism, increasing its efficiency with regard to **5** and **6**, its catalytic activity is still moderate. Thus, only 56% of the ketone is obtained with this catalyst, after 24 h. The dehydrogenation of 1,2-phenylenedimethanol (Scheme 5c) in the presence of **7** affords 1-isobenzofuranone and molecular hydrogen in a quantitative yield after 24 h, whereas 70% of the lactone is formed after 12 h. The reaction yield in the presence of the pincer complex **3** is also good, 86%.

## Concluding Remarks

This study shows that the hexahydride complex OsH_6_(P^i^Pr_3_)_2_ promotes the C–H bond activation of aryl compounds asymmetrically 1,3-disubstituted, with two coordinating groups. The rationalization of the products formed in the reactions with the [BF_4_]^−^ and [BPh_4_]^−^ salts of the cations 1-(3-(isoquinolin-1-yl)phenyl)-3-methylimidazolium and 1-(3-(isoquinolin-1-yl)phenyl)-3-methylbenzimidazolium reveals that the azolium substituent establishes the position of the C–H bond activation. This is due to the main role of the azolium group during the activation, which can be governed through the election of the anion of the salt, and to the existence of significant differences in behavior between the azolium groups depending upon their imidazolium or benzimidazolium nature. The [BF_4_]^−^ anion inhibits the reactions of the azolium groups. Consistently, both [BF_4_]^−^ salts undergo the rupture of the aryl-CH bond at the 4-position, as a consequence of an isoquinolinyl-assisted C–H bond activation reaction. In contrast, the [BPh_4_]^−^ anion disinhibits the azolium reactions. Then, the imidazolium substituent affords an imidazolylidene group. This moiety preferentially coordinates to the metal center in an abnormal fashion, to direct the C–H bond activation of the aryl at the 2-position and finally to yield a pincer ligand by the coordination of the isoquinolyl substituent. In contrast, the benzimidazolylidene, resulting from the deprotonation of the benzimidazolium substituent, assists the C–H bond activation at the 6-position.

The pincer complex resulting from the C–H activation of the central aryl group at the 2-position and the coordination of both substituents possesses interesting features. Its hydride ligands show an intense quantum mechanical exchange coupling, and it is a red phosphorescent emitter upon photoexcitation and displays a noticeable catalytic activity for the dehydrogenation of 1-phenylethanol to acetophenone and 1,2-phenylenedimethanol to 1-isobenzofuranone.

The sequential C–H bond activation of the 6- and 4-positions of the central aryl group of the 1-(3-(isoquinolin-1-yl)phenyl)-3-methylbenzimidazolium tetraphenylborate affords a bimetallic compound, which displays catalytic synergism between the metals, for the dehydrogenation of 1,2,3,4-tetrahydroisoquinoline and alcohols. The synergism seems to result from the electronic coupling between the metal centers and gives rise to a noticeable catalytic activity of this compound in the dehydrogenation of 1,2-phenylenedimethanol to 1-isobenzofuranone.

In summary, the introduction of an azolium substituent into a phenyl group previously bearing a coordinating group, to form an asymmetrically 1,3-disubstituted aryl salt, allows an efficient governing of the C–H bond activation of the aromatic ring. As a result, compounds with interesting physical properties and new catalysts can be prepared.

## Experimental Section

### General Information

All reactions were carried out with exclusion of air using Schlenk-tube techniques or in a drybox. Instrumental methods and X-ray details are given in the Supporting Information. The chemical shifts (in ppm) in the NMR spectra (Figures S1–S36) are referenced to residual solvent peaks (^1^H, ^13^C{^1^H}) or external 85% H_3_PO_4_ (^31^P{^1^H}) or CFCl_3_ (^19^F{^1^H}), while the coupling constants *J* and *N* (*N* = *J*_P–H_ + *J*_P′–H_ for ^1^H and *N* = *J*_P–C_ + *J*_P′–C_ for ^13^C{^1^H}) are given in hertz.

### Reaction of OsH_6_(P^i^Pr_3_)_2_ (**1**) with 1-(3-(Isoquinolin-1-yl)phenyl)-3-methylimidazolium Tetrafluoroborate: Preparation of **2**

A mixture of **1** (200 mg, 0.387 mmol), 1-(3-(isoquinolin-1-yl)phenyl)-3-methylimidazolium tetrafluoroborate (145 mg, 0.387 mmol), and triethylamine (809 μL, 5.805 mmol) in toluene (8 mL) was refluxed for 24 h, giving a red suspension. After the mixture was cooled to room temperature, the solvent was removed *in vacuo*, affording a red residue. A small portion of the residue was dissolved in dichloromethane-*d*_2_, and its ^1^H and ^31^P{^1^H} NMR spectra showed the formation of **2** in 82% yield. Addition of pentane (3 mL) caused the precipitation of a pale red solid, which was washed with pentane (3 × 3 mL) and dried *in vacuo*. Yield: 250 mg (73%). Anal. Calcd for C_37_H_60_BF_4_N_3_OsP_2_: C, 50.16; H, 6.83; N, 4.74. Found: C, 49.78; H, 6.73; N, 4.97. HRMS (electrospray, *m*/*z*): calculated for C_37_H_60_N_3_OsP_2_ [M]^+^, 800.3951; found, 800.3874. IR (cm^–1^): ν(Os–H) 1975 (w), ν(BF_4_) 1016 (vs). ^1^H NMR (300.13 MHz, CD_2_Cl_2_, 298 K): δ 9.49–6.87 (12H, C_19_H_15_N_3_), 4.11 (s, 3H, CH_3_), 1.78 (m, 6H, PC*H*(CH_3_)_2_), 0.89 (dvt, ^3^*J*_H–H_ = 7.0, *N* = 12.4, 36H, PCH(C*H*_3_)_2_), −8.25 (br, 2H, Os–H), −11.97 (br, 1H, Os–H). ^1^H NMR (300 MHz, CD_2_Cl_2_, high-field region, 193 K): δ −6.19 (br, 1H Os–H), −10.71 (br, 1H, Os–H), −12.10 (br, 1H, Os–H). ^13^C{^1^H}-apt NMR (75.48 MHz, CD_2_Cl_2_, 298 K): δ 199.1 (t, ^3^*J*_C–P_ = 5.8, Os–C Ph), 164.6 (s, C), 152.9, 148.1 (both s, CH), 146.1 (s, C), 135.4 (s, CH), 134.6 (s, C), 129.6, 128.5 (both s, CH), 127.3 (s, C), 127.2, 126.7 (both s, CH), 125.6 (s, C), 124.3, 123.2, 122.1, 119.6, 119.4 (all s, CH), 37.0 (s, CH_3_), 26.6 (vt, *N* = 24.1, P*C*H(CH_3_)_2_), 20.0, 19.7 (both s, PCH(*C*H_3_)_2_). ^31^P{^1^H} NMR (121.50 MHz, CD_2_Cl_2_, 298 K): δ 22.1 (s). ^19^F{^1^H} NMR (282.38 MHz, CD_2_Cl_2_, 298 K): δ −153.7 (s). *T*_1_(min) (ms, OsH, 300 MHz, CD_2_Cl_2_, 213 K): 32 ± 3 (−6.19 ppm); 32 ± 3 (−10.71 ppm); 85 ± 8 (−12.10 ppm).

### Reaction of OsH_6_(P^i^Pr_3_)_2_ (**1**) with 1-(3-(Isoquinolin-1-yl)phenyl)-3-methylimidazolium Tetraphenylborate: Preparation of **3** and **4**

A mixture of **1** (200 mg, 0.387 mmol), 1-(3-(isoquinolin-1-yl)phenyl)-3-methylimidazolium tetraphenylborate (330 mg, 0.503 mmol), and triethylamine (809 μL, 5.805 mmol) in toluene (8 mL) was refluxed for 24 h, giving a dark brown suspension. After the mixture was cooled to room temperature, the solvent was removed *in vacuo*, affording a brown residue. A small portion of the residue was dissolved in dichloromethane-*d*_2_, and its ^1^H and ^31^P{^1^H} NMR spectra showed the formation of **3** and **4** in a 73/27 molar ratio. The brown residue was purified by flash column chromatography (silica gel, toluene/CH_2_Cl_2_ 100/0 to 0/100). Yellow and red bands were eluted, and after the solvents were removed, yellow and red residues were obtained. Addition of pentane (3 mL) to each of them afforded yellow and red solids, respectively, that were washed with pentane (2 × 4 mL) and dried *in vacuo*. Yield of **3** (red compound): 246 mg (57%). Yield of **4** (yellow compound): 28 mg (9%).

#### Data for **3**

Anal. Calcd for C_61_H_78_BN_3_OsP_2_: C, 65.63; H, 7.04; N, 3.76. Found: C, 65.22; H, 6.92; N, 4.01. HRMS (electrospray, *m*/*z*): calculated for C_37_H_58_N_3_OsP_2_ [M]^+^, 798.3701; found, 798.3717. IR (cm^–1^): ν(Os–H) 2034 (w). ^1^H NMR (300.13 MHz, CD_2_Cl_2_, 298 K): δ 9.13–6.30 (31 H, 11H C_19_H_14_N_3_ plus 20H BPh_4_), 3.50 (s, 3H, CH_3_), 1.70 (m, 6H, PC*H*(CH_3_)_2_), 0.80 (dvt, ^3^*J*_H–H_ = 6.7, *N* = 13.0, 18H, PCH(C*H*_3_)_2_), 0.69 (dvt, ^3^*J*_H–H_ = 6.5, *N* = 13.0, 18H, PCH(C*H*_3_)_2_), −4.54 (br, 2H, Os–H). ^1^H{^31^P} NMR (300 MHz, CD_2_Cl_2_, high-field region, 193 K): δ −5.72 (AB spin system, Δν = 2678, *J*_A-B_ = 135, 2H, OsH). ^13^C{^1^H}-apt NMR (75.48 MHz, CD_2_Cl_2_, 298 K): δ 192.4 (t, ^2^*J*_C–P_ = 5.9, Os–C Ph), 167.2 (s, C), 164.5 (q, *J*_C–B_ = 49.1, C BPh_4_), 153.2 (s, CH), 144.9 (s, C), 143.3 (s, C), 140.2 (t, ^2^*J*_C–P_ = 7.3, Os–C im), 136.3 (s, CH BPh_4_), 135.6 (s, C), 135.5, 131.0, 128.7, 127.5, 127.3, 126.9 (all s, CH), 126.2 (q, *J*_C–B_ = 2.1, CH BPh_4_), 124.1 (s, CH), 122.2 (s, CH BPh_4_), 121.3, 121.2, 111.8 (all s, CH), 35.8 (s, CH_3_), 26.2 (vt, *N* = 25.1, P*C*H(CH_3_)_2_), 19.0, 18.9 (both s, PCH(*C*H_3_)_2_). ^31^P{^1^H} NMR (121.50 MHz, CD_2_Cl_2_, 298 K): δ 3.4 (s). *T*_1_ (ms, OsH, 300 MHz, CD_2_Cl_2_, 183 K): 279 ± 28 (−5.72 ppm).

#### Data for **4**

Anal. Calcd for C_37_H_59_N_3_OsP_2_: C, 55.68; H, 7.45; N, 5.27. Found: C, 55.47; H, 7.18; N, 5.32. HRMS (electrospray, *m*/*z*) calculated for C_37_H_59_N_3_OsP_2_ [M]^+^, 796.3604; found, 796.3561. IR (cm^–1^): ν(Os–H) 2033 (w). ^1^H NMR (300.13 MHz, CD_2_Cl_2_, 298 K): δ 8.54–6.94 (11H, C_19_H_14_N_3_), 3.96 (s, 3H, CH_3_), 1.82 (m, 6H, PC*H*(CH_3_)_2_), 0.97 (dvt, ^3^*J*_H–H_ = 6.3, *N* = 12.4, 18H, PCH(C*H*_3_)_2_), 0.88 (dvt, ^3^*J*_H–H_ = 6.4, *N* = 12.6, 18H, PCH(C*H*_3_)_2_), −8.77 (br, 1H, Os–H), −10.41 (br, 2H, Os–H). ^1^H NMR (300 MHz, CD_2_Cl_2_, high-field region, 183 K): δ −8.90 (t, ^2^*J*_H–P_ = 15.7, 1H, Os–H), −10.38 (br, 1H, Os–H), −10.78 (br, 1H, Os–H). ^13^C{^1^H}-apt NMR (75.48 MHz, CD_2_Cl_2_, 298 K): δ 190.1 (t, ^2^*J*_C–P_ = 5.9, Os–C im), 162.8 (t, ^2^*J*_C–P_ = 7.6, Os–C Ph), 162.7, 147.7 (both s, C), 147.3, 142.6 (both s, CH), 137.5, 130.2 (both s, C), 129.9, 128.7 (both s, CH), 127.3 (s, C), 127.1, 126.9, 125.8, 120.9, 118.8, 114.7, 111.3 (all s, CH), 39.8 (s, CH_3_), 28.2 (vt, *N* = 24.4, P*C*H(CH_3_)_2_), 19.9, 19.8 (both s, PCH(*C*H_3_)_2_). ^31^P{^1^H} NMR (121.50 MHz, CD_2_Cl_2_, 298 K): δ 24.7 (s). *T*_1_(min) (ms, OsH, 300 MHz, CD_2_Cl_2_, 223 K): 70 ± 7 (−8.79 ppm); 75 ± 7 (−10.47 ppm).

### Reaction of OsH_6_(P^i^Pr_3_)_2_ (**1**) with 1-(3-(Isoquinolin-1-yl)phenyl)-3-methylbenzimidazolium Tetrafluoroborate: Preparation of **5**

A mixture of **1** (200 mg, 0.387 mmol), 1-(3-(isoquinolin-1-yl)phenyl)-3-methylbenzimidazolium tetrafluoroborate (164 mg, 0.387 mmol), and triethylamine (809 μL, 5.805 mmol) in toluene (8 mL) was refluxed for 24 h, giving a burgundy suspension. After the mixture was cooled to room temperature, the solvent was removed *in vacuo*, affording a burgundy residue. A small portion of the residue was dissolved in dichloromethane*-d*_2_, and its ^1^H and ^31^P{^1^H} NMR spectra showed the formation of **5** in 82% yield. Addition of diethyl ether (3 mL) to the residue caused the precipitation of a dark red solid, which was washed with diethyl ether (3 × 3 mL) and dried *in vacuo*. Yield: 265 mg (73%). Anal. Calcd for C_41_H_62_BF_4_N_3_OsP_2_: C, 52.61; H, 6.68; N, 4.49. Found: C, 52.72; H, 7.03; N, 4.72. HRMS (electrospray, *m*/*z*): calculated for C_41_H_60_N_3_OsP_2_ [M – 2H], 848.3865; found, 848.3874. IR (cm^–1^): ν(BF_4_) 1019 (vs). ^1^H NMR (300.13 MHz, CD_2_Cl_2_, 298 K): δ 9.54–6.94 (14H, C_23_H_17_N_3_), 4.32 (s, 3H, CH_3_), 1.83 (m, 6H, PCH(C*H*_3_)_2_), 0.93 (dvt, ^3^*J*_H–H_ = 6.8, *N* = 12.3, 36H, PCH(C*H*_3_)_2_), −8.23 (br, 2H, Os–H), −11.90 (br, 1H, Os–H). ^1^H NMR (300 MHz, CD_2_Cl_2_, high-field region, 203 K): δ −6.19 (br, 1H, Os–H), −10.54 (br, 1H, Os–H), −11.99 (t, ^2^*J*_H–P_ = 9.9, 1H, Os–H). ^13^C{^1^H}-apt NMR (75.48 MHz, CD_2_Cl_2_, 298 K): δ 199.7 (t, ^3^*J*_C–P_ = 5.5,Os–C), 164.9 (s, C), 152.9 (s, CH), 148.2 (s, CH), 146.5 (s, C), 141.5 (s, CH), 135.4, 132.9, 132.5 (all s, C), 129.8, 128.5, 127.9, 127.8 (all s, CH), 127.4 (s, C), 127.1 (s, CH), 126.4 (s, CH), 122.9 (s, C), 122.2, 119.5, 114.2, 113.3 (all s, CH), 34.0 (s, CH_3_), 27.8 (vt, *N* = 24.3, PCH(C*H*_3_)_2_), 20.1, 19.8 (both s, PCH(C*H*_3_)_2_). ^31^P{^1^H} NMR (121.50 MHz, CD_2_Cl_2_, 298 K): δ 21.9 (s). ^19^F{^1^H} NMR (282.38 MHz, CD_2_Cl_2_, 298 K): δ −151.4 (s). *T*_1_(min) (ms, OsH, 300 MHz, CD_2_Cl_2_, 253 K): 61 ± 6 (−8.24 ppm); 33 ± 3 (−11.90 ppm).

### Reaction of OsH_6_(P^i^Pr_3_)_2_ (**1**) with 1-(3-(Isoquinolin-1-yl)phenyl)-3-methylbenzimidazolium Tetraphenylborate: Preparation of **6**

A mixture of **1** (200 mg, 0.387 mmol), 1-(3-(isoquinolin-1-yl)phenyl)-3-methylbenzimidazolium tetraphenylborate (330 mg, 0.503 mmol), and triethylamine (809 μL, 5.805 mmol) in toluene (8 mL) was refluxed for 24 h, giving a dark brown suspension. After the mixture was cooled to room temperature, the solvent was removed *in vacuo*, affording a brown residue. A small portion of the residue was dissolved in dichloromethane*-d*_2_, and its ^1^H and ^31^P{^1^H} NMR spectra showed the formation of **6** in 78% yield. The brown residue was purified by flash column chromatography (silica gel, toluene), eluting a yellow band. Upon evaporation of toluene and addition of pentane (3 mL) a yellow solid was obtained, which was washed with pentane (2 × 4 mL) and dried *in vacuo*. Yield: 141 mg (43%). Anal. Calcd for C_41_H_61_N_3_OsP_2_: C, 58.06; H, 7.25; N, 4.95. Found: C, 58.31; H, 7.16; N, 5.35. HRMS (electrospray, *m*/*z*): calculated for C_41_H_60_N_3_OsP_2_ [M – H]^+^, 848.3879; found, 848.3874. IR (cm^–1^): ν(Os–H) 1904 (w). ^1^H NMR (300.13 MHz, CD_2_Cl_2_, 298 K): δ 8.61–7.05 (13H, C_23_H_16_N_3_), 4.24 (s, 3H, CH_3_), 1.90 (m, 6H, PCH(C*H*_3_)_2_), 0.99 (dvt, ^3^*J*_H–H_ = 7.0, *N* = 12.6, 18H, PCH(C*H*_3_)_2_), 0.90 (dvt, ^3^*J*_H–H_ = 7.0, *N* = 12.6, 18H, PCH(C*H*_3_)_2_), −8.40 (br, 1H, Os–H), −9.97 (br, 2H, Os–H). ^1^H NMR (300 MHz, CD_2_Cl_2_, high-field region, 233 K): δ −8.43 (tt, ^2^*J*_H–H_ = 7.0, ^2^*J*_H–P_ = 17.4,1H, Os–H), −10.02 (dt, ^2^*J*_H–H_ = 7.0, ^2^*J*_H–P_ = 14.0, 2H, Os–H). ^13^C{^1^H}-apt NMR (75.48 MHz, CD_2_Cl_2_, 298 K): δ 206.1 (t, ^2^*J*_C–P_ = 5.8, Os–C), 162.5 (s, C), 161.9 (t, ^2^*J*_C–P_ = 5.6, Os–C), 148.6 (s, C), 147.0 (s, CH), 142.3 (s, CH), 137.3, 137.1, 133.1, 130.2 (all s, C), 129.5, 128.2 (both s, CH), 126.9 (s, C), 126.7, 126.6, 125.1, 121.6, 121.1, 118.4, 113.1, 110.0, 109.1 (all s, CH), 36.7 (s, CH_3_), 27.9 (vt, *N* = 24.8, P*C*H(CH_3_)_2_), 19.4, 19.3 (both s, PCH(*C*H_3_)_2_). ^31^P{^1^H} NMR (121.50 MHz, CD_2_Cl_2_, 298 K): δ 26.1 (s). *T*_1_(min) (ms, OsH, 300 MHz, CD_2_Cl_2_, 223 K): 59 ± 6 (−8.45 ppm); 66 ± 7 (−10.02 ppm).

### Reaction of Complex 6 with OsH_6_(P^i^Pr_3_)_2_ (**1**): Preparation of **7**

A solution of complexes **1** (79 mg, 0.153 mmol) and **6** (130 mg, 0.153 mmol) in toluene (5 mL) was refluxed for 24 h, giving a burgundy suspension. After the mixture was cooled to room temperature, the solvent was removed *in vacuo*, affording a burgundy residue. A small portion of the residue was dissolved in dichloromethane*-d*_2_, and its ^1^H and ^31^P{^1^H} NMR spectra showed the formation of **7** in 90% yield. Addition of methanol (3 mL) caused the precipitation of a garnet solid that was washed with methanol (3 × 4 mL) and dried *in vacuo*. Yield: 166 mg (80%). Anal. Calcd for C_59_H_105_N_3_Os_2_P_4_: C, 52.07; H, 7.78; N, 3.09. Found: C, 52.13; H, 7.56; N, 3.03. HRMS (electrospray, *m*/*z*): calculated for C_59_H_105_N_3_Os_2_P_4_ [M]^+^, 1360.5830; found, 1360.5691. IR (cm^–1^): ν(Os–H) 1989 (w), 1880 (m). ^1^H NMR (300.13 MHz, C_6_D_6_, 298 K): δ 9.57–6.67 (12H, C_23_H_15_N_3_), 3.98 (s, 3H, CH_3_), 1.99 (m, 12H, PC*H*(CH_3_)_2_), 1.14 (dvt, ^3^*J*_H–H_ = 7.0, *N* = 12.6, 36H, PCH(C*H*_3_)_2_), 1.04 (dvt, ^3^*J*_H–H_ = 7.0, *N* = 12.6, 18H, PCH(C*H*_3_)_2_), 0.96 (dvt, ^3^*J*_H–H_ = 7.0, *N* = 12.6, 18H, PCH(C*H*_3_)_2_), −8.02 (br, 1H, Os–H), −10.13 (br, 3H, Os–H), −11.24 (br, 2H, Os–H). ^1^H NMR (300 MHz, toluene-*d*_8_, high-field region, 203 K): δ −6.23 (br, 1H, Os–H), −8.05 (br, 1H, Os–H), −10.05 (br, 2H, Os–H), −11.04 (br, 2H, Os–H). ^13^C{^1^H}-apt NMR (75.48 MHz, C_6_D_6_, 298 K): δ 204.9 (t, ^2^*J*_C–P_ = 5.7, Os–C BzIm), 186.5 (t, ^2^*J*_C–P_ = 6.1, Os–C Ph), 168.5 (s, C), 166.6 (t, ^2^*J*_C–P_ = 5.9, Os–C Ph), 164.4 (s, CH), 153.2 (s, CH), 143.4 (s, C), 137.7 (s, C), 137.7 (s, C), 137.2 (s, CH), 136.2 (s, C), 135.9 (s, CH), 133.4 (s, C), 126.8, 125.9, 122.1, 120.9, 115.4, 115.1, 110.3, 109.2 (all s, CH), 36.8 (s, CH_3_), 28.1 (vt, *N* = 24.1, P*C*H(CH_3_)_2_), 26.4 (vt, *N* = 23.4, P*C*H(CH_3_)_2_), 21.0, 20.2, 20.0, 19.9 (all s, PCH(*C*H_3_)_2_). ^31^P{^1^H} NMR (121.50 MHz, C_6_D_6_, 298 K): δ 26.7 (s), 22.6 (s). *T*_1_(min) (ms, OsH, 300 MHz, toluene-*d*_8_, 248 K): 88 ± 8 (−6.23 ppm); 115 ± 11 (−8.05 ppm), 96 ± 10 (−10.05 ppm), 88 ± 8 (−11.04 ppm).

### General Procedure for the Catalytic Dehydrogenation of 1,2,3,4-Tetrahydroisoquinoline

Under an argon atmosphere a solution of the catalyst (**2**, **3**, **5**, or **6**, 0.0082 mmol; **7**, 0.0041 mmol) and 1,2,3,4-tetrahydroisoquinoline (0.12 mmol) in *p*-xylene (1 mL) was placed in a Schlenk flask equipped with a condenser. The reaction mixture was heated at 140 °C for 48 h, and then it was cooled to room temperature. Gas chromatography was used to determine the extent of the reactions (Agilent Technologies 4890D gas chromatograph with a flame ionization detector, HP-5 column (30 m × 0.32 mm, with 0.25 μm film thickness), oven conditions 80 °C (hold 1 min) to 220 °C at 10 °C/min (hold 2 min)). The reactions were run in duplicate. The identity of the products was confirmed by comparison of their retention times with those of pure samples, as well as by GC-MS analyses.

### General Procedure for the Catalytic Dehydrogenation of Alcohols

A toluene solution (1 mL) of the catalyst (**2**, **3**, **5**, or **6**, 0.0082 mmol; **7**, 0.0041 mmol) and the corresponding alcohol (0.12 mmol) was placed in a Schlenk flask equipped with a condenser. The reaction mixture was heated at 100 °C for 24 h. After this time the solution was cooled to room temperature, and the yield of the reaction was determined by different methods depending on the nature of the alcohol. In the case of 1-phenylethanol, the extent of the conversion to acetophenone was determined by GC on an Agilent Technologies 6890N gas chromatograph equipped with a flame ionization detector, using a HP-Innowax column (30 m × 0.25 mm, with 0.25 μm film thickness; oven conditions 80 °C (hold 5 min) to 200 °C at 15 °C/min (hold 7 min). The identity of the ketone was confirmed by a GC-MS analysis and by a comparison of its retention time with that of a pure sample. For benzyl alcohol and 1,2-phenylenedimethanol, the crude solution was concentrated under reduced pressure to obtain an oil. Then, 1,1,2,2-tetrachloroethane was added as an internal standard, and the mixture was dissolved in CDCl_3_ and analyzed by ^1^H NMR spectroscopy. Benzaldehyde and 1-isobenzofuranone were characterized by ^1^H NMR spectroscopy. The reactions were run in duplicate.

## Supporting Information Available

General information for the Experimental Section, structural analysis, NMR spectra, computational details and energies of optimized structures, experimental and computed UV–vis and UV–vis–NIR spectra, cyclic voltammogram of complex **7**, and frontier orbitals (PDF)
Cartesian coordinates of the optimized structures (XYZ)

## Supplementary Material

om1c00565_si_001.pdf

om1c00565_si_002.xyz

## Accession Codes

CCDC 2112116–2112119 contain the supplementary crystallographic data for this paper. These data can be obtained free of charge via www.ccdc.cam.ac.uk/data_request/cif, or by emailing data_request@ccdc.cam.ac.uk, or by contacting The Cambridge Crystallographic Data Centre, 12 Union Road, Cambridge CB2 1EZ, UK; fax: +44 1223 336033.
