# Supplementary material for: Azolium Control of the Osmium-Promoted Aromatic C–H Bond Activation in 1,3-Disubstituted Substrates
Source: Organometallics. 2021 Nov 18;40(23):3979–91. doi: 10.1021/acs.organomet.1c00565 (PMC8672810; doi:10.1021/acs.organomet.1c00565)
Supplement: Supplementary file 1 — om1c00565_si_001.pdf [file om1c00565_si_001.pdf]

SUPPORTING INFORMATION

Azolium Control of the Osmium-Promoted Aromatic C-H Bond Activation in 1,3-Disubstituted Substrates

Lara Cancela, Miguel A. Esteruelas,* Montserrat Oliván, and Enrique Oñate

Departamento de Química Inorgánica – Instituto de Síntesis Química y Catálisis Homogénea (ISQCH) – Centro de Innovación en Química Avanzada (ORFEO-CINQA), Universidad de Zaragoza – CSIC, 50009 Zaragoza, Spain

* e-mail: maester@unizar.es

Contents:

- Experimental Details S2
- Structural Analysis of Complexes **2**, **3**, **4** and **7** S3
- NMR Spectra S5
- Computational Details S41
- Energies of the Optimized Structures S42
- UV-vis Spectrum of Complex **7** (Observed and Calculated) S46
- Theoretical Analysis of the Molecular Orbitals of Complex **7** S47
- Cyclic Voltammogram of Complex **7** S52
- Optimized Structures of Complexes **2-7**, [**7**]$^{+}$, [**7**]$^{2+}$, [**7**]$^{3+}$, and [**7**]$^{4+}$ S53
- UV-vis-NIR Spectrum of Complex **7** S58
- UV-vis-NIR Spectra of [**7**]$^{+}$ and [**7**]$^{3+}$ (Observed and Calculated) S61
- Calculated HOMO, SOMO, and LUMO of Complexes **7**, [**7**]$^{+}$, [**7**]$^{2+}$, [**7**]$^{3+}$, and [**7**]$^{4+}$ S62
- References S65

● **Experimental Details**

General Information. All reactions were carried out with exclusion of air using Schlenk-tube techniques or in a drybox. Pentane, dichloromethane and toluene were obtained oxygen- and water-free from an MBraun solvent purification apparatus, while methanol, *p*-xylene and 2-MeTHF were dried and distilled under argon prior to use. ^{1}H, $^{13}C\{^{1}H\}$, $^{31}P\{^{1}H\}$ and $^{19}F\{^{1}H\}$ NMR spectra were recorded on Bruker 300 ARX or Bruker Avance 300 MHz. Chemical shifts (expressed in ppm) are referenced to residual solvent peaks (^{1}H, $^{13}C\{^{1}H\}$), external 85% H_3PO_4 ($^{31}P\{^{1}H\}$) or $CFCl_3$ (^{19}F). Coupling constant J and N ($N = J_{P\text{-}H} + J_{P'\text{-}H}$ for ^{1}H and $N = J_{P\text{-}C} + J_{P'\text{-}C}$ for $^{13}C\{^{1}H\}$) are given in hertz. Attenuated total reflection infrared spectra (ATR-IR) of solid samples were run on a PerkinElmer Spectrum 100 FT-IR spectrometer. Elemental analyses were carried out in a PerkinElmer 2400 CHNS/O analyzer. High-resolution electrospray mass spectra were acquired using a MicroTOF-Q hybrid quadrupole time-of-flight spectrometer (Bruker Daltonics, Bremen, Germany). UV-visible spectra were recorded on an Evolution 600 spectrophotomer. Steady-state photoluminescence spectra were recorded on a Jobin-Yvon Horiba Fluorolog FL-3-11 spectrofluorimeter. Lifetimes were measured using an IBH 5000F coaxial nanosecond flash lamp. Quantum yields were measured using the Hamamatsu Absolute PL Quantum Yield Measurement System C11347-11. Spectroelectrochemical studies were performed with a micro-Autolab FRA2 Type III (Methrom, Utrecht, Netherlands) potentiostat controlled by NOVA (v.2.1.4) software, and connected to a JASCO V670 spectrophotomer using a DRP-PTGRID-TRANSCELL (DropSens). Cyclic voltammetry measurements were performed using a Voltalab PST050 potentiostat with Pt wire as working electrode, Pt wire as counter electrode, and saturated calomel (SCE) as reference electrode. The experiments were carried out under argon in dichloromethane solutions (10^{-3} M), with $[Bu_4N]PF_6$ as supporting electrolyte (0.1 M). Scan rate was 100 mV s^{-1}. The potentials were referenced to the ferrocene/ferrocenium (Fc/Fc^{+}) couple. $OsH_6(P^{i}Pr_3)_2$ was prepared as reported previously.[1]

● **Structural Analysis of Complexes 2, 3, 4 and 7**

X-ray data were collected for the complex on a Bruker D8 Venture (**2**, **3**), and APEX CCD (**4**, **7**) diffractometers (Mo radiation, λ = 0.71073 Å). The crystals were cooled with a nitrogen flow from Oxford Cryosystems systems. Data were corrected for absorption by using a multiscan method applied with the SADABS program.[2] The structures were solved by Patterson or direct methods and refined by full-matrix least squares on F^2 with SHELXL2016,[3] including isotropic and subsequently anisotropic displacement parameters. The hydrogen atoms were observed in the last Fourier Maps or calculated, and refined freely or using a restricted riding model.

Crystal data for **2** (CCDC 2112116): $C_{37}H_{60}N_3OsP_2$ x BF_4 x CH_3OH, M_W 917.87, orange, irregular block (0.280 x 0.190 x 0.040 mm^3), monoclinic, space group $P2_1/c$, *a*: 16.0610(14) Å, *b*: 44.558(4) Å, *c*: 11.6417(10) Å, *β*: 104.673(2)°, *V* = 8059.6(12) $Å^3$, *Z* = 8, *Z'* = 2, D_{calc}: 1.513 g cm^{-3}, F(000): 3744, T = 100(2) K, μ 3.296 mm^{-1}. 268753 measured reflections (2θ: 3-57°, ω scans 0.3°), 20157 unique (R_{int} = 0.0499); min./max. transm. factors 0.669/0.862. Final agreement factors were R^1 = 0.0424 (19703 observed reflections, I > 2σ(I)) and wR^2 = 0.0873; data/restraints/parameters 20157/8/953; GoF = 1.316. Largest peak and hole 1.782 (close to osmium atoms) and -2.782 e/$Å^3$.

Crystal data for **3** (CCDC 2112117): $C_{37}H_{58}N_3OsP_2$, $C_{24}H_{20}B$, M_W 1116.21, orange, irregular block (0.115 x 0.100 x 0.048 mm^3), triclinic, space group P-1, *a*: 10.9170(5) Å, *b*: 13.9285(6) Å, *c*: 19.5915(9) Å, *α*: 105.7057(17)°, *β*: 99.2931(17)°, *γ*: 106.3914(17)°, *V* = 2658.4(2) $Å^3$, *Z* = 2, *Z'* = 1, D_{calc}: 1.394 g cm^{-3}, F(000): 1152, T = 100(2) K, μ 2.500 mm^{-1}. 156313 measured reflections (2θ: 3-57°, ω scans 0.3°), 13205 unique (R_{int} = 0.1440); min./max. transm. factors 0.784/0.862. Final agreement factors were R^1 = 0.0254 (12897 observed reflections, I > 2σ(I)) and wR^2 = 0.0863; data/restraints/parameters 13205/2/ 635; GoF = 0.735. Largest peak and hole 1.813 (close to osmium atoms) and -2.047e/$Å^3$.

Crystal data for **4** (CCDC 2112118): $C_{38}H_{63}N_3OOsP_2$, M_W 830.05, yellow, irregular block (0.221 x 0.158 x 0.070 mm^3), triclinic, space group P-1, *a*: 9.9901(6) Å, *b*: 10.9693(7) Å, *c*: 18.3523(11) Å, *α*: 75.4550(10)°, *β*: 74.8160(10)°, *γ*: 87.2370(10)°, *V* = 1878.4(2) $Å^3$, *Z* = 2, *Z'* = 1, D_{calc}: 1.468 g cm^{-3}, F(000): 852, T = 100(2) K, μ 3.512 mm^{-1}. 17204 measured reflections (2θ: 3-57°, ω scans 0.3°), 8714 unique (R_{int} = 0.0269);

min./max. transm. factors 0.677/0.862. Final agreement factors were R^1 = 0.0291 (7892 observed reflections, I > 2σ(I)) and wR^2 = 0.0668; data/restraints/parameters 8714/3/430; GoF = 1.051. Largest peak and hole 2.176 (close to osmiun atoms) and -2.176 e/Å^3.

Crystal data for **7** (CCDC 2112119): $C_{59}H_{105}N_3Os_2P_4$, M_W 1360.73, red, irregular block (0.203 x 0.135 x 0.120 mm^3), triclinic, space group P-1, *a*: 11.8207(6) Å, *b*: 14.5470(8) Å, *c*: 18.3298(10) Å, *α*: 86.3900(10)°, *β*: 82.9850(10)°, *γ*: 71.6630(10)°, *V* = 2968.5(3) Å^3, *Z* = 2, *Z'* = 1, D_{calc}: 1.522 g cm^{-3}, F(000): 1384, T = 150(2) K, μ 4.423 mm^{-1}. 53113 measured reflections (2θ: 3-57°, ω scans 0.3^o), 14290 unique (R_{int} = 0.0296); min./max. transm. factors 0.718/0.862. Final agreement factors were R^1 = 0.0292 (12702 observed reflections, I > 2σ(I)) and wR^2 = 0.0699; data/restraints/parameters 14290/14/ 652; GoF = 1.036. Largest peak and hole 2.039 (close to osmium atoms) and -1.509 e/Å^3.

• **NMR Spectra**

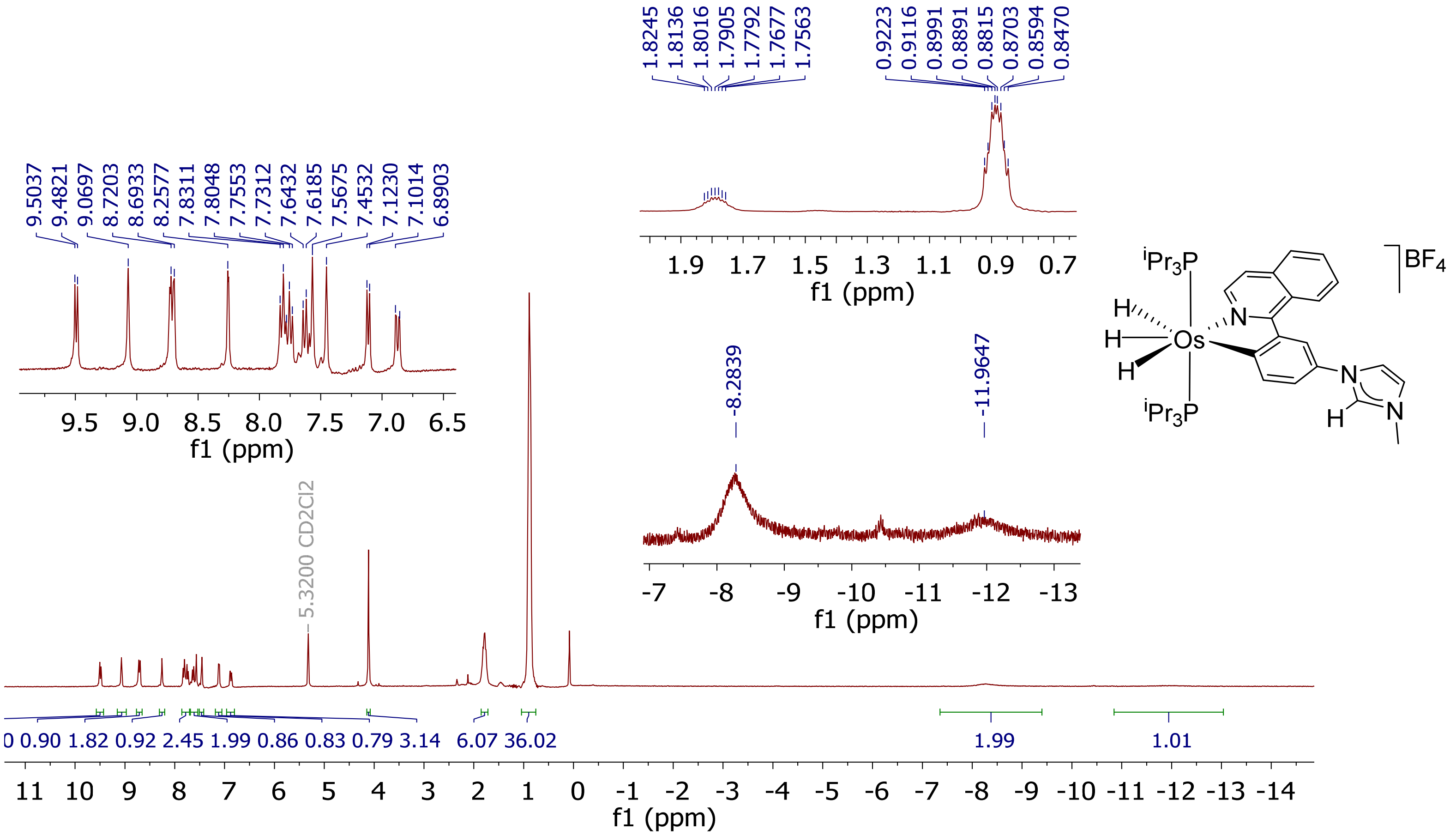

Figure S1. ^{1}H NMR spectrum (300.13 MHz, CD_2Cl_2, 298 K) of compound **2**

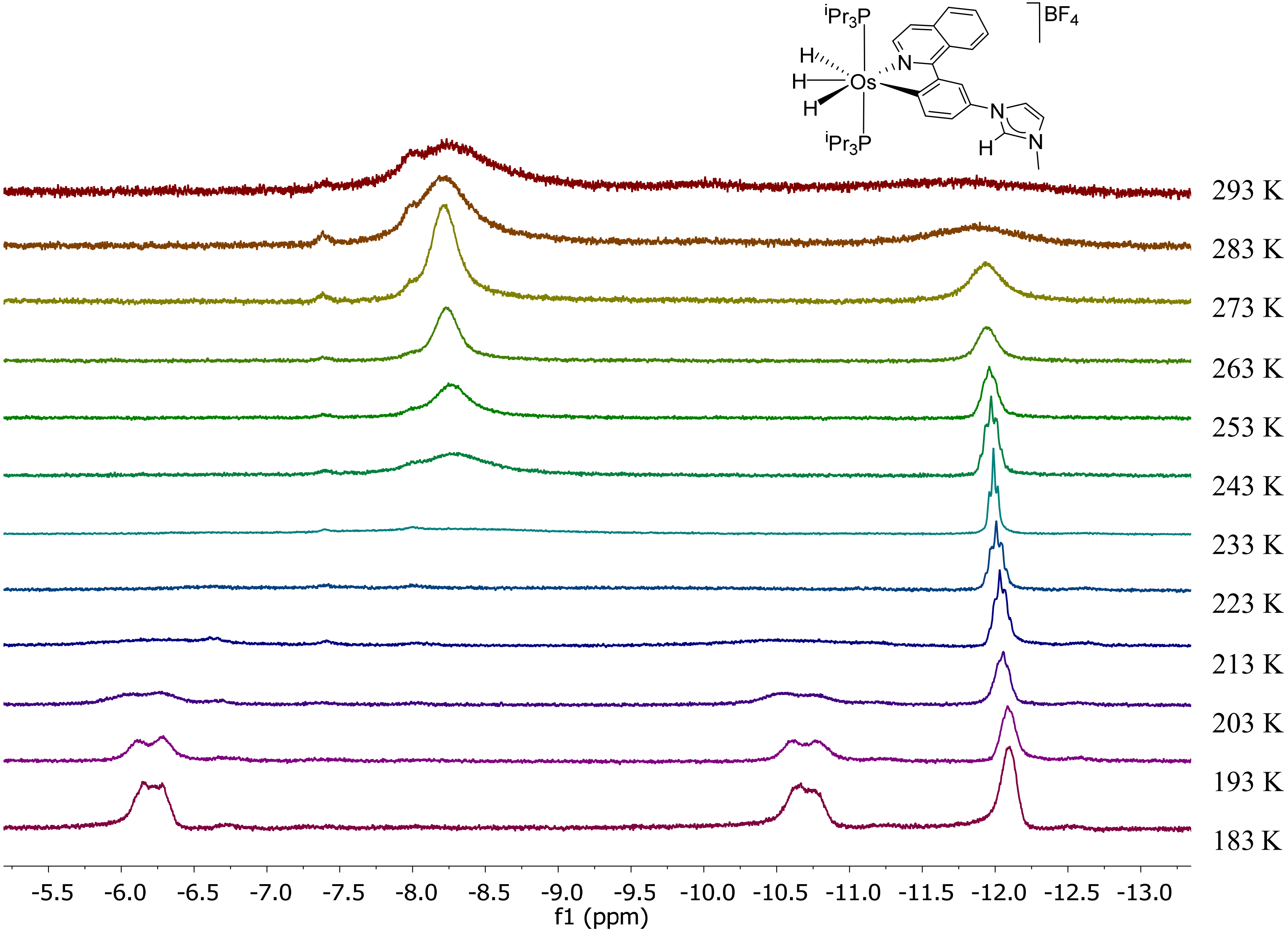

Figure S2. High field region of the ^1H NMR spectra (300.13 MHz, CD_2Cl_2) of compound **2** as a function of the temperature.

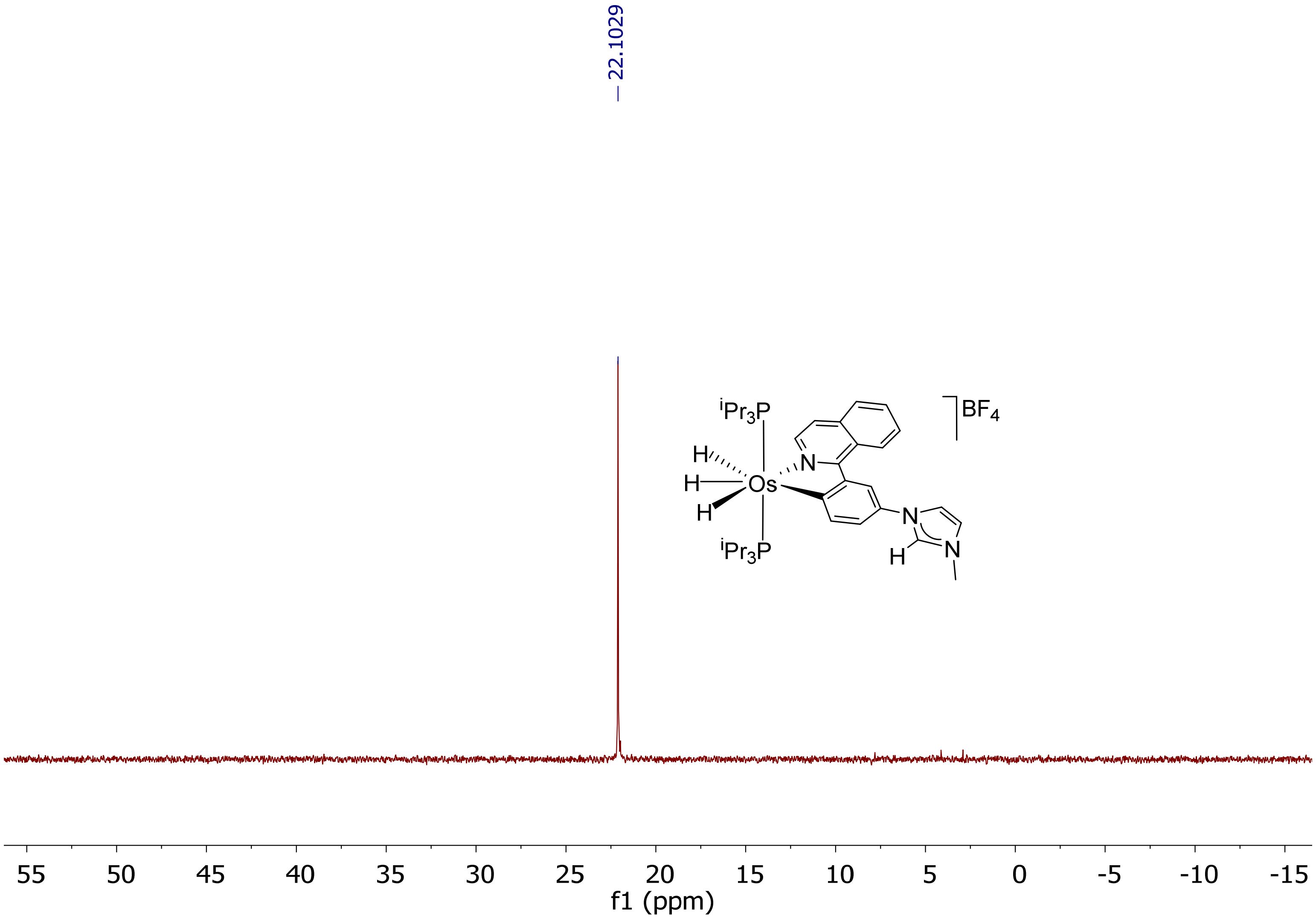

Figure S3. $^{31}P\{^1H\}$ NMR spectrum (121.49 MHz, CD_2Cl_2, 298 K) of compound **2**.

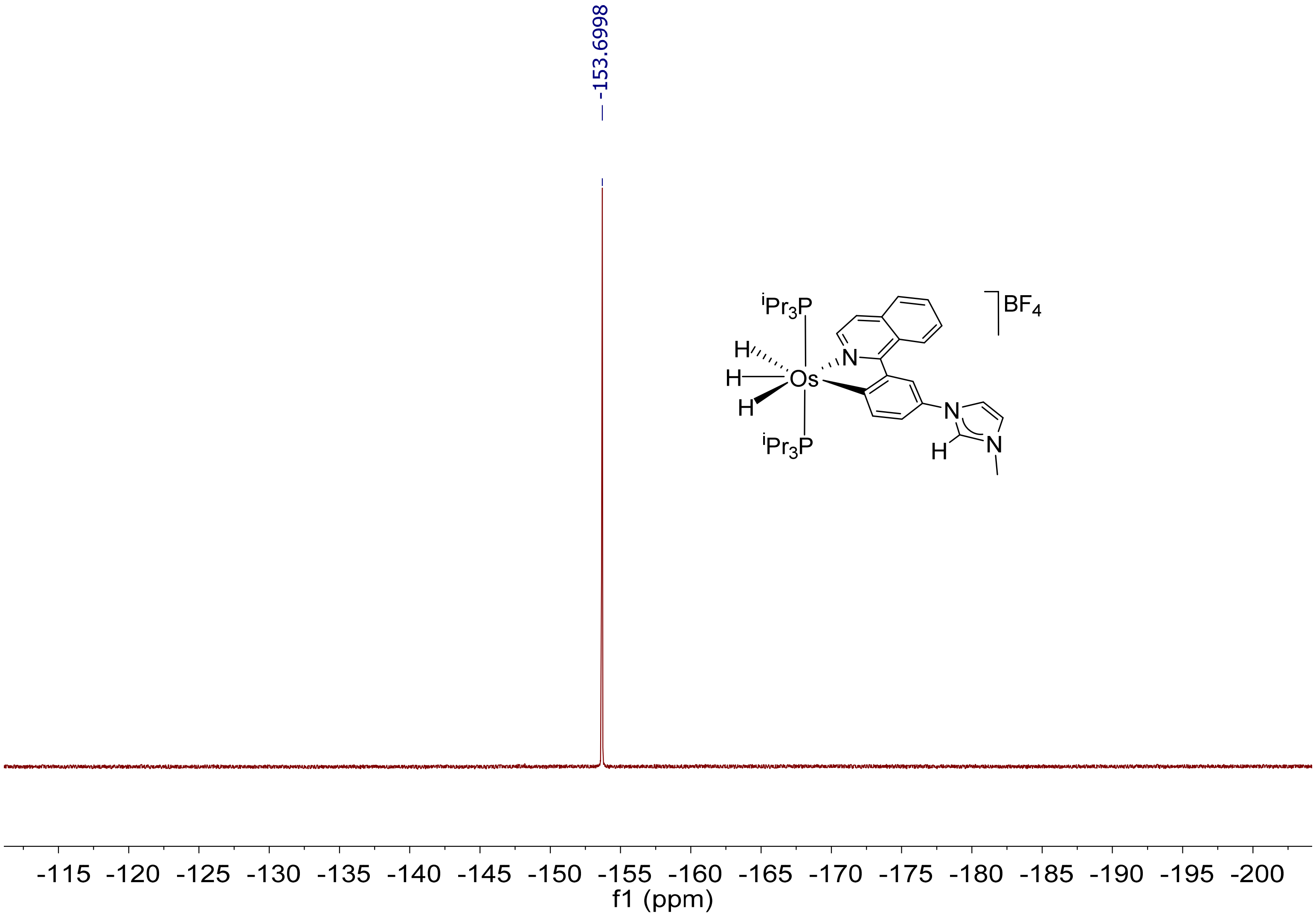

Figure S4. $^{19}F\{^{1}H\}$ NMR spectrum (282.38 MHz, CD_2Cl_2, 298 K) of compound **2**.

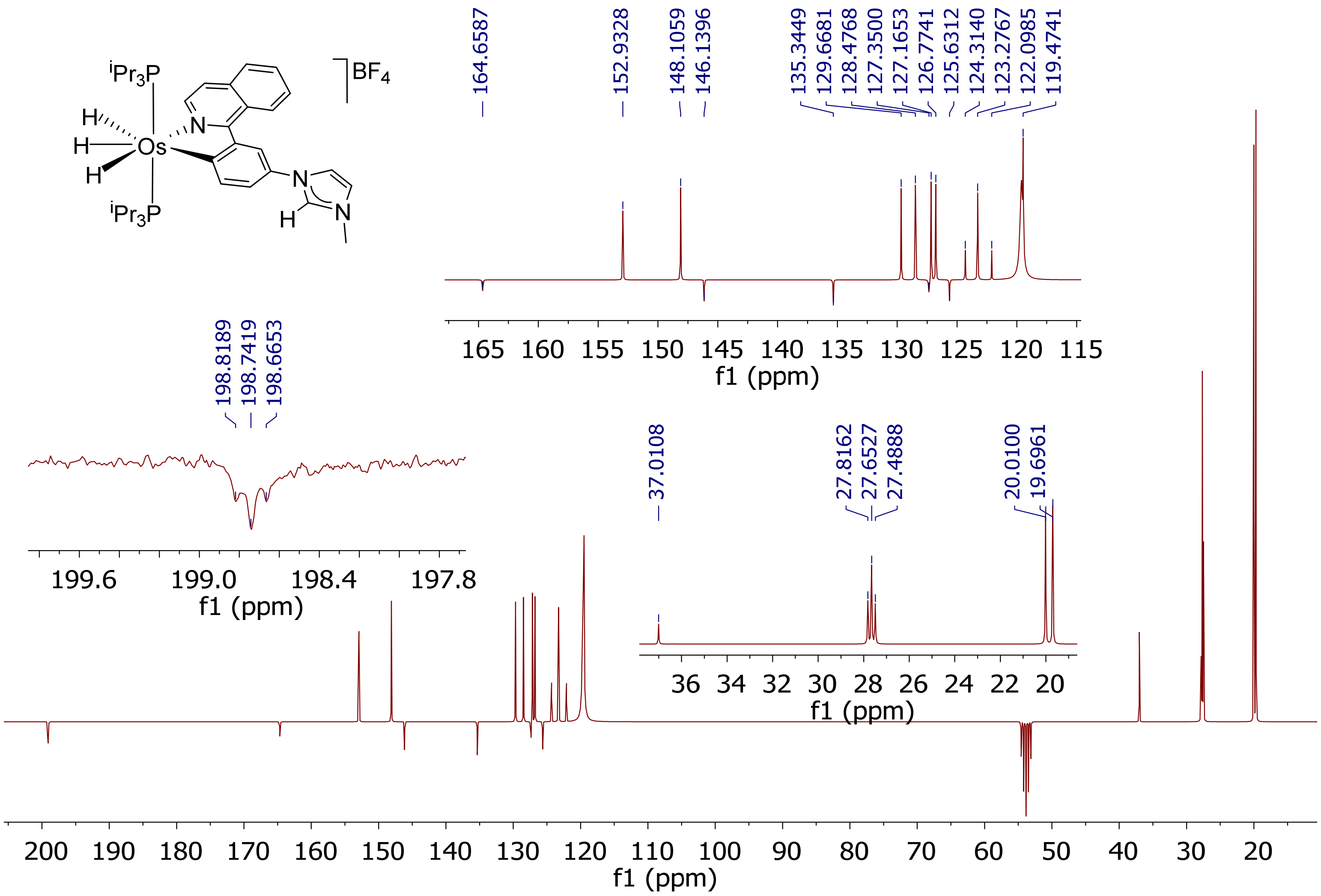

Figure S5. $^{13}C\{^{1}H\}$-apt NMR spectrum (75.48 MHz, CD_2Cl_2, 298 K) of compound **2**.

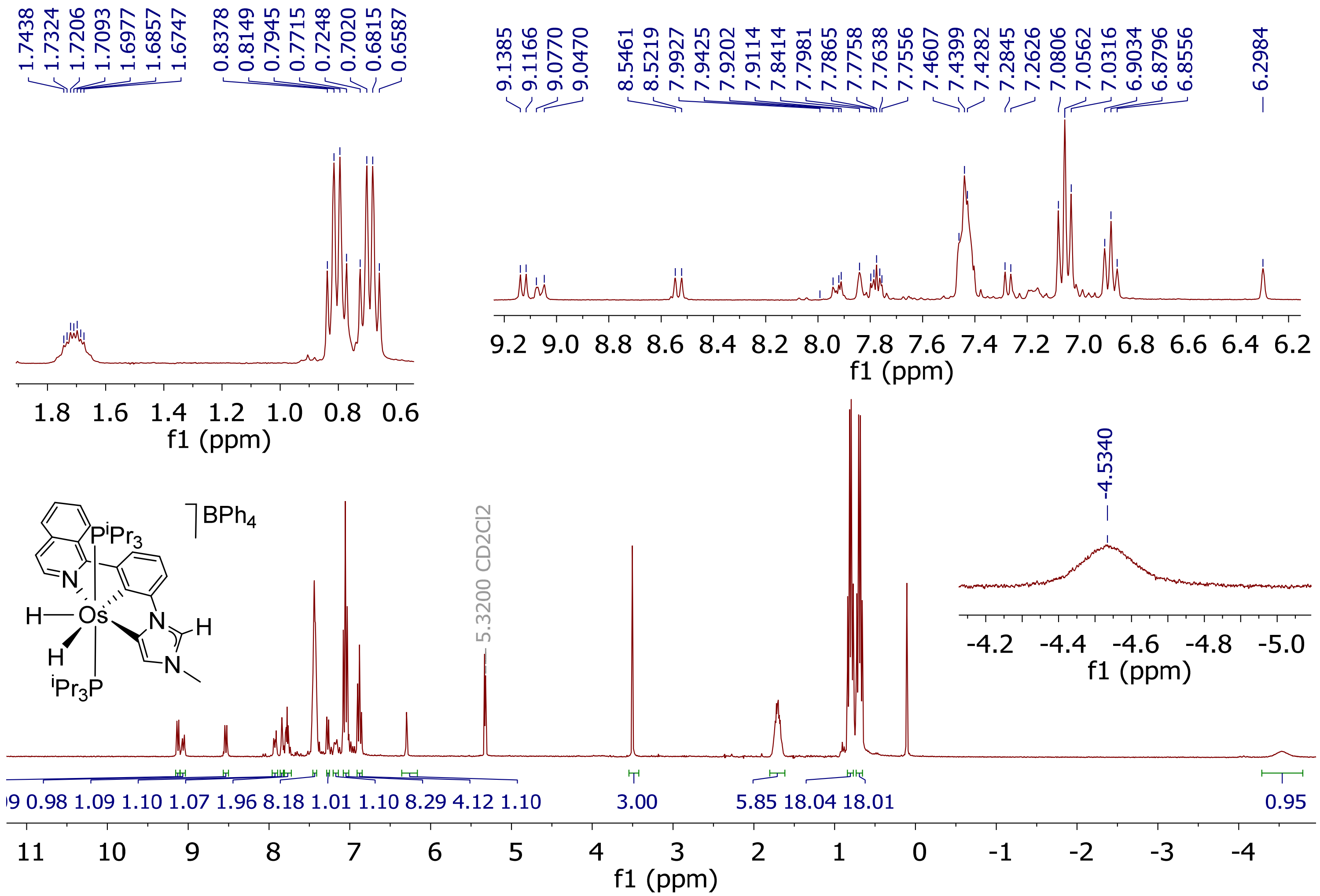

Figure S6. ^{1}H NMR spectrum (300.13 MHz, CD_2Cl_2, 298 K) of compound **3**.

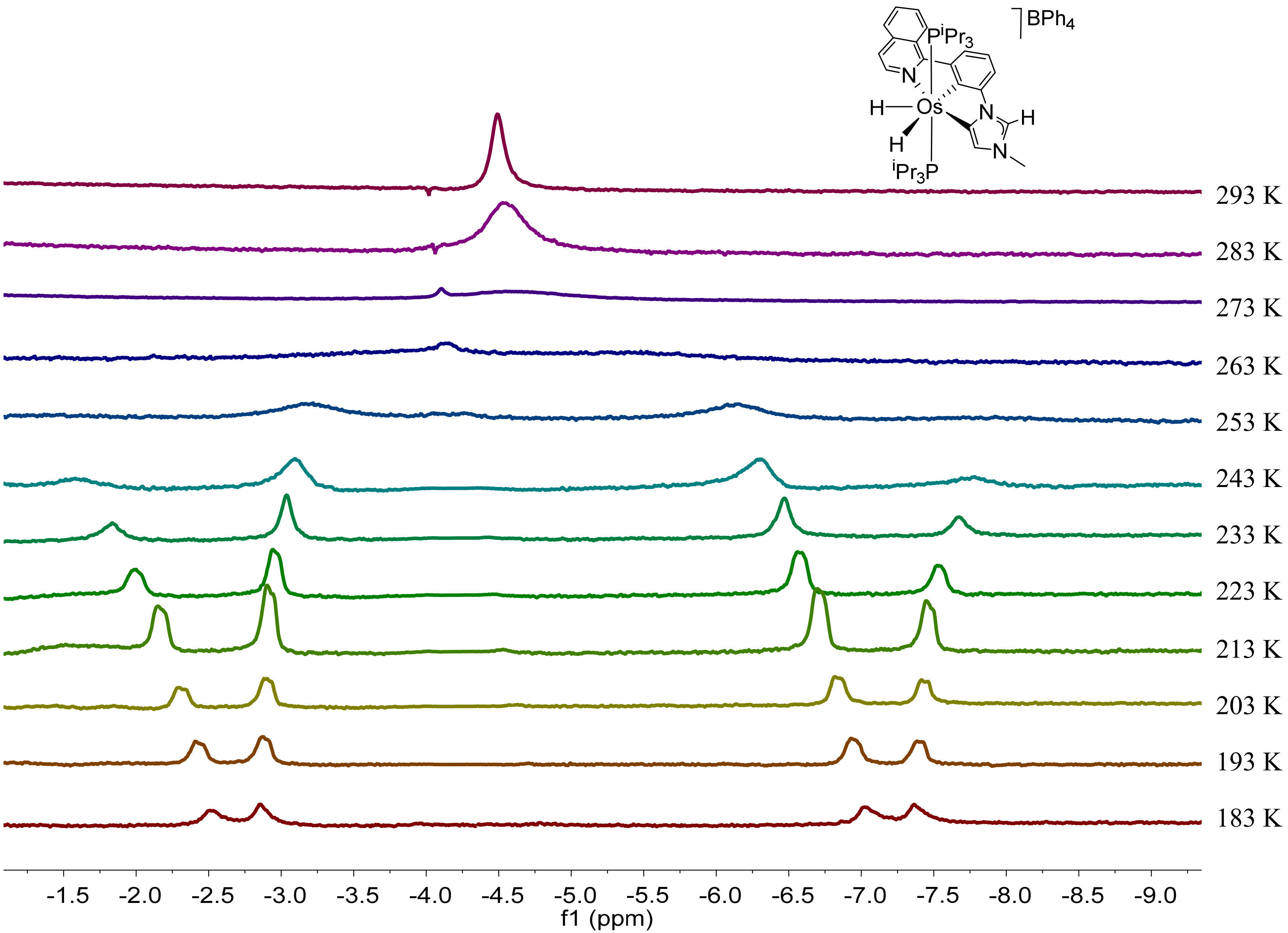

Figure S7. High field region of the $^{1}H\{^{31}P\}$ NMR spectra (300.13 MHz, CD_2Cl_2) of compound **3** as a function of the temperature.

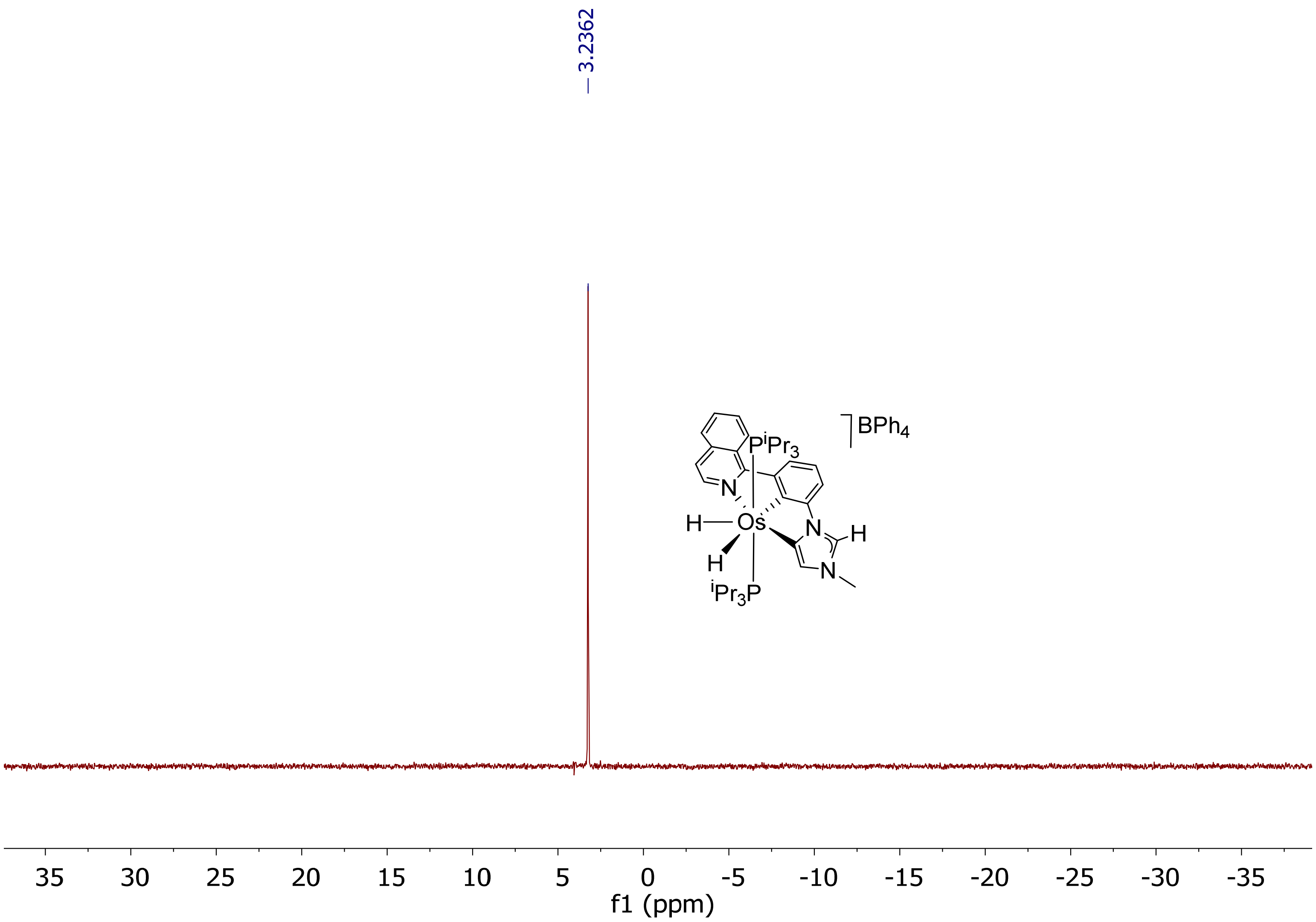

Figure S8. $^{31}P\{^{1}H\}$ NMR spectrum (121.49 MHz, CD_2Cl_2, 298 K) of compound **3**.

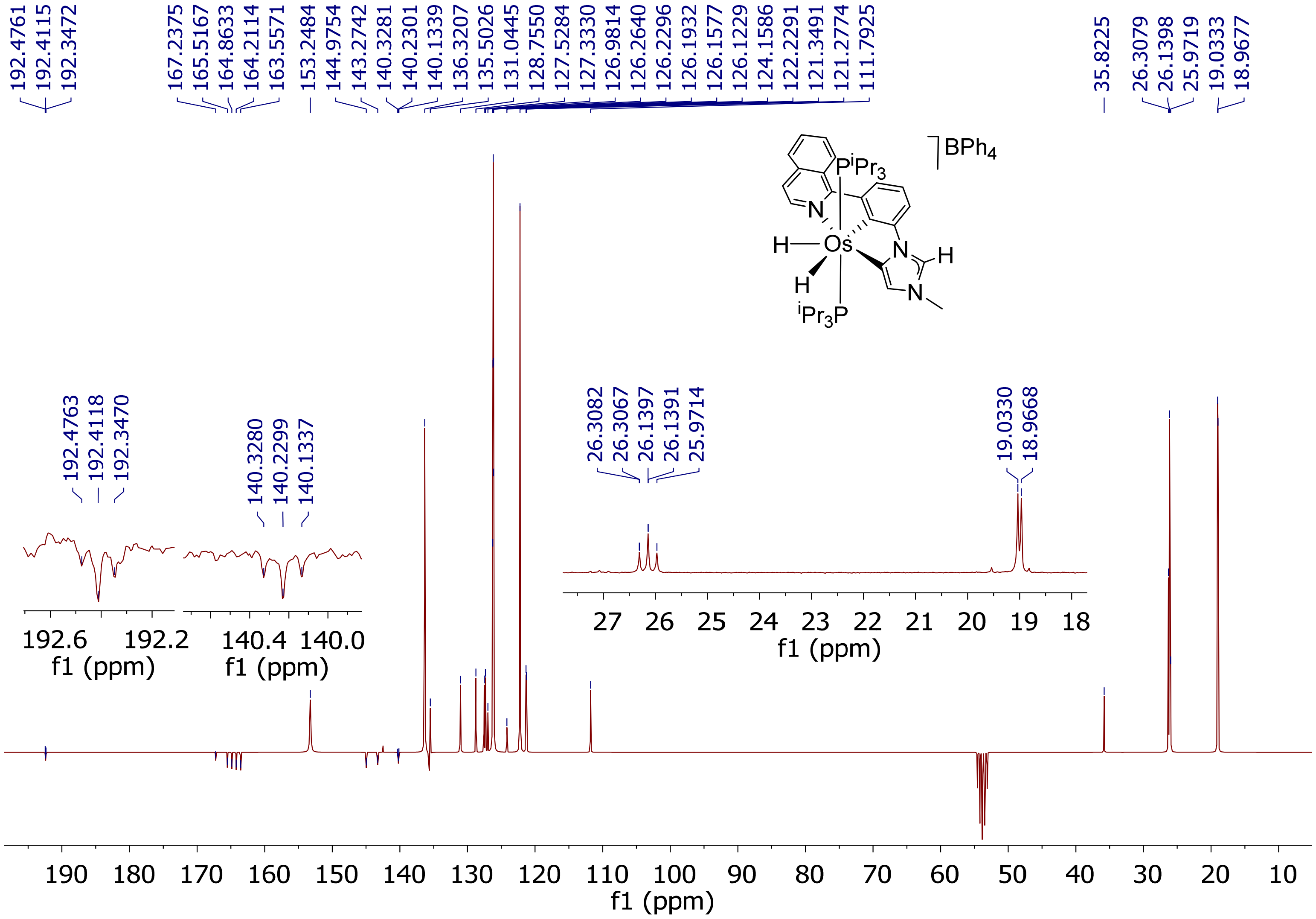

Figure S9. $^{13}C\{^{1}H\}$-apt NMR spectrum (75.48 MHz, CD_2Cl_2, 298 K) of compound **3**.

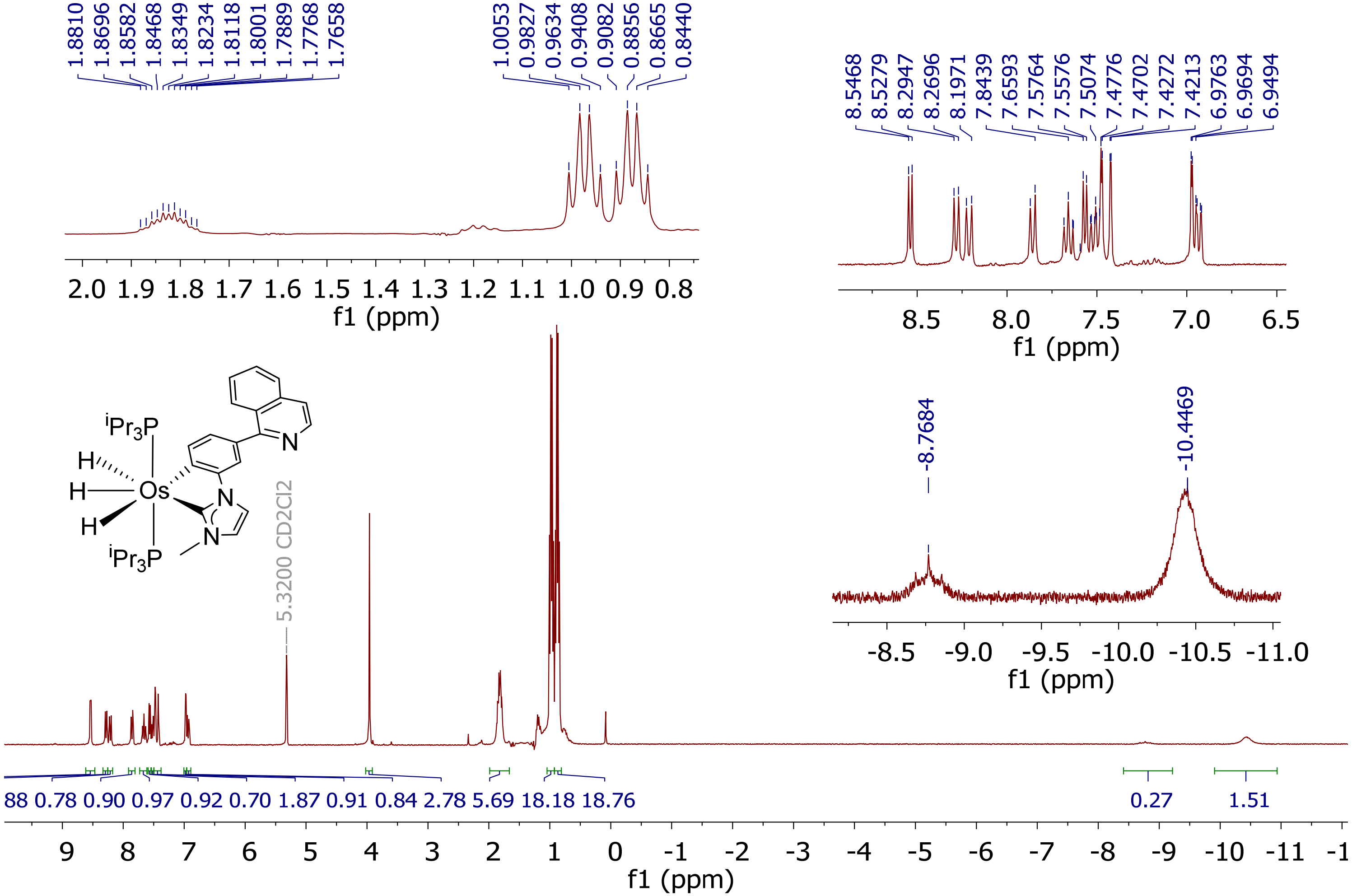

Figure S10. ^{1}H NMR spectrum (300.13 MHz, CD_2Cl_2, 298 K) of compound **4**.

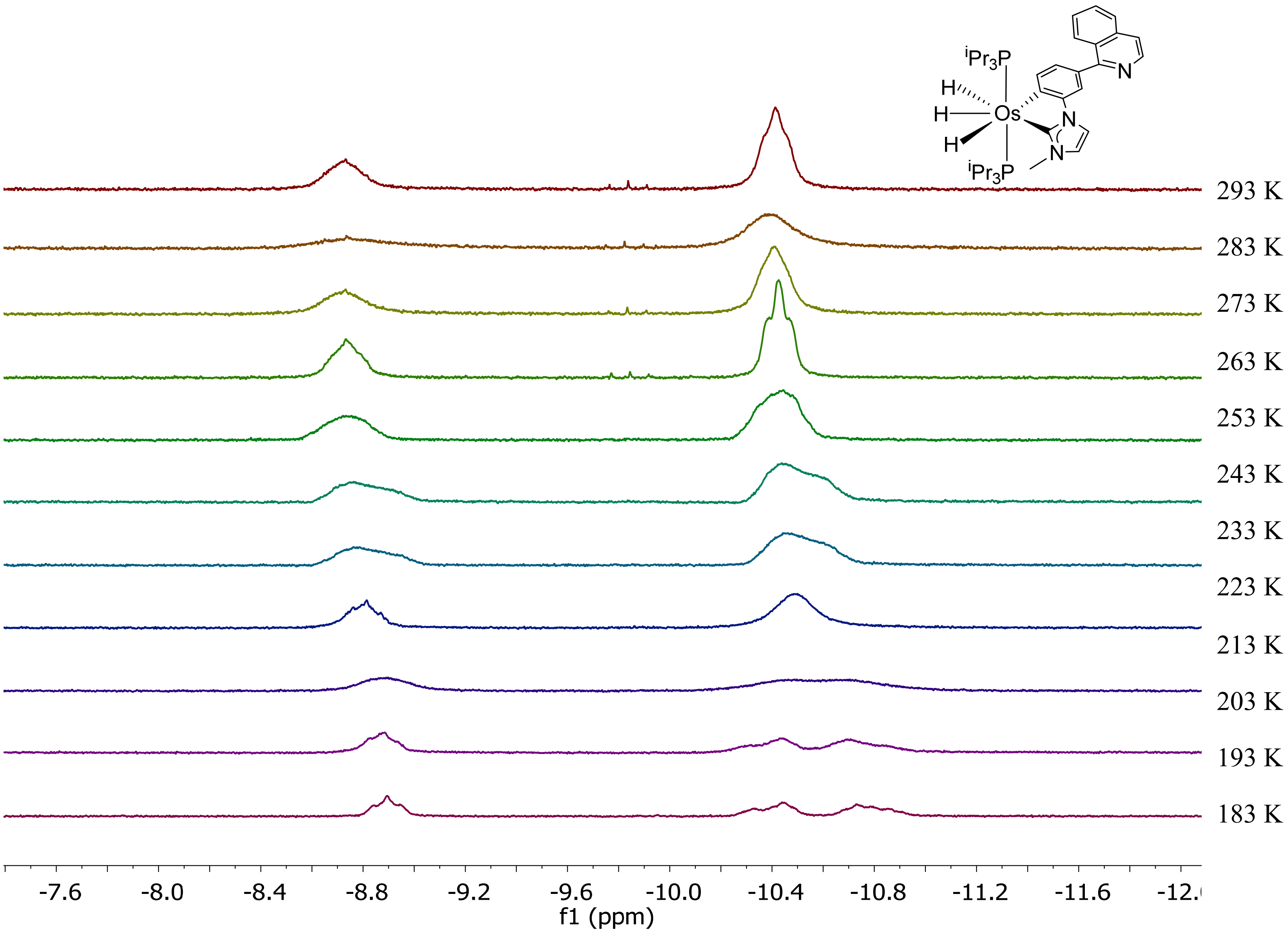

Figure S11. High field region of the ^{1}H NMR spectra (300.13 MHz, CD_2Cl_2) of compound **4** as a function of the temperature.

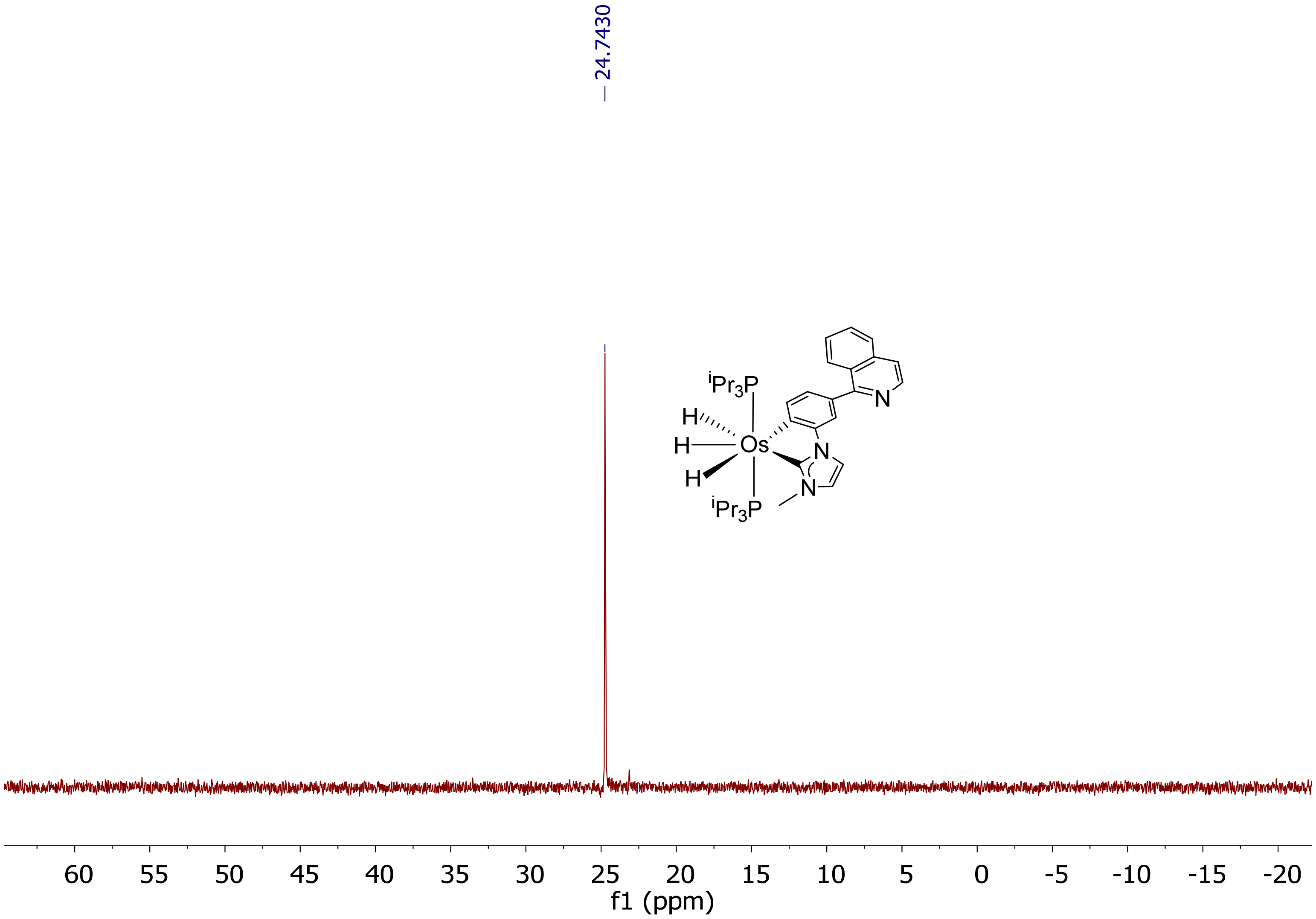

Figure S12. $^{31}P\{^{1}H\}$ NMR spectrum (121.49 MHz, CD_2Cl_2, 298 K) of compound **4**.

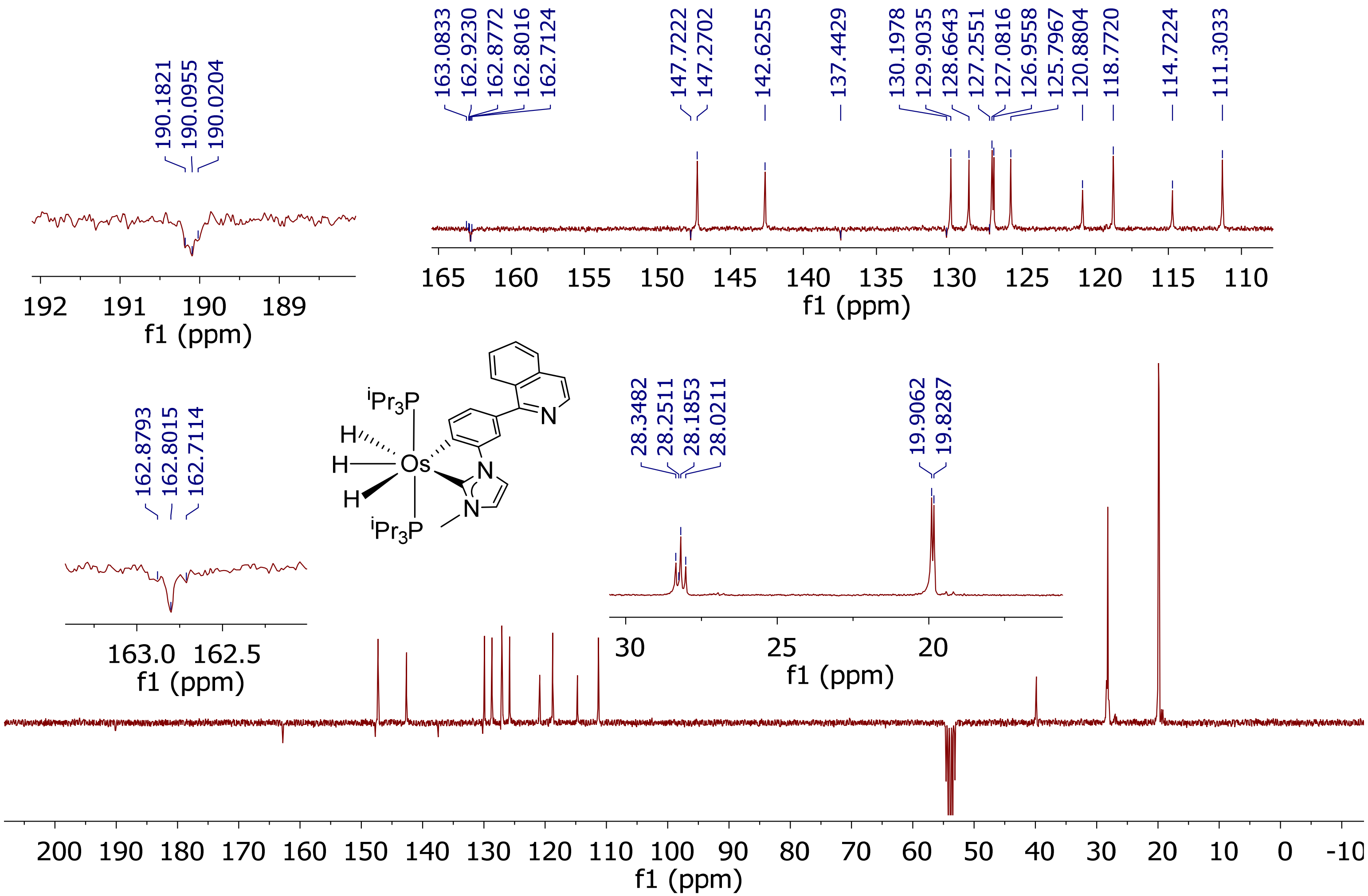

Figure S13. $^{13}C\{^{1}H\}$-apt NMR spectrum (75.48 MHz, CD_2Cl_2, 298 K) of compound **4**.

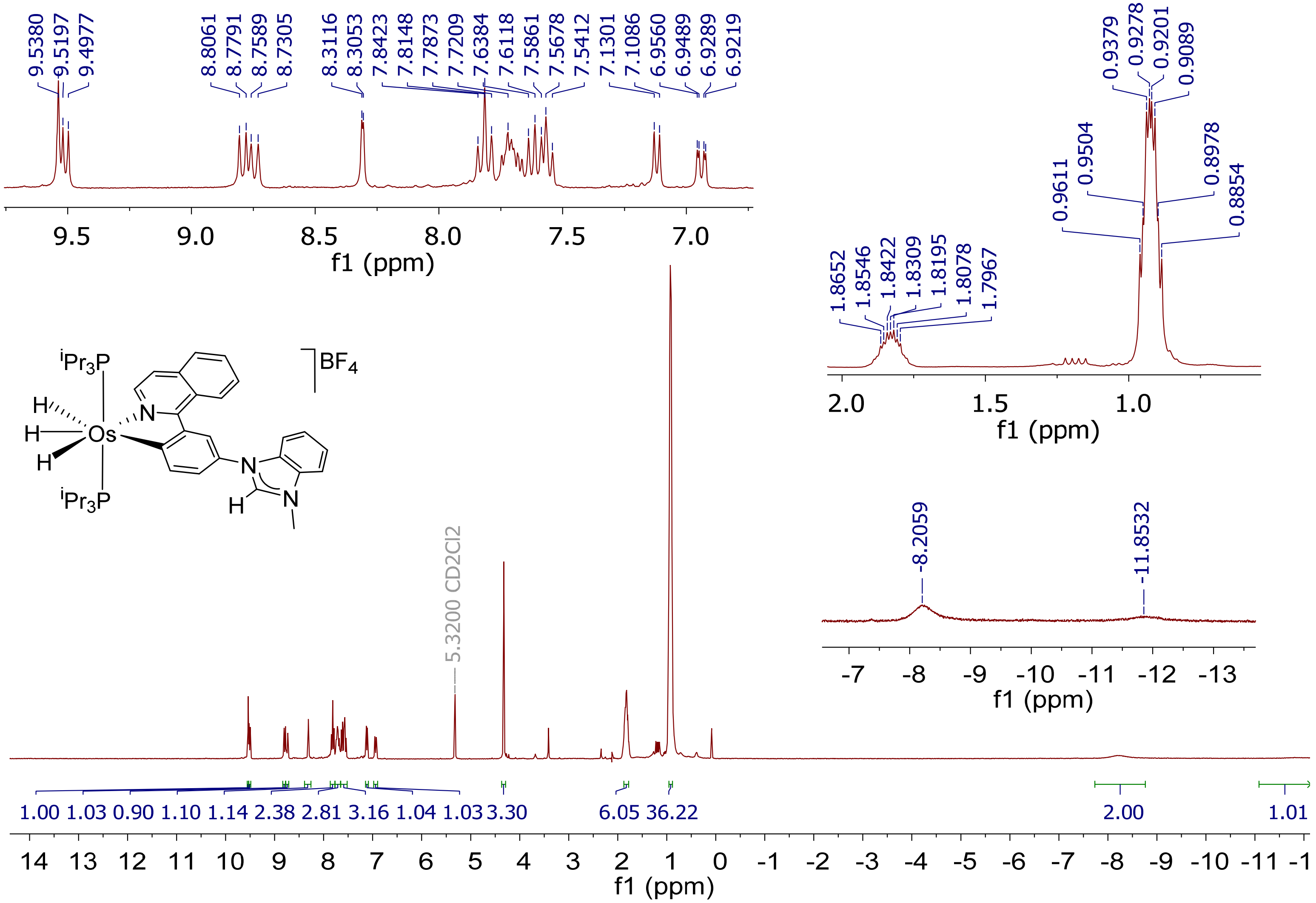

Figure S14. ^{1}H NMR spectrum (300.13 MHz, CD_2Cl_2, 298 K) of compound **5**.

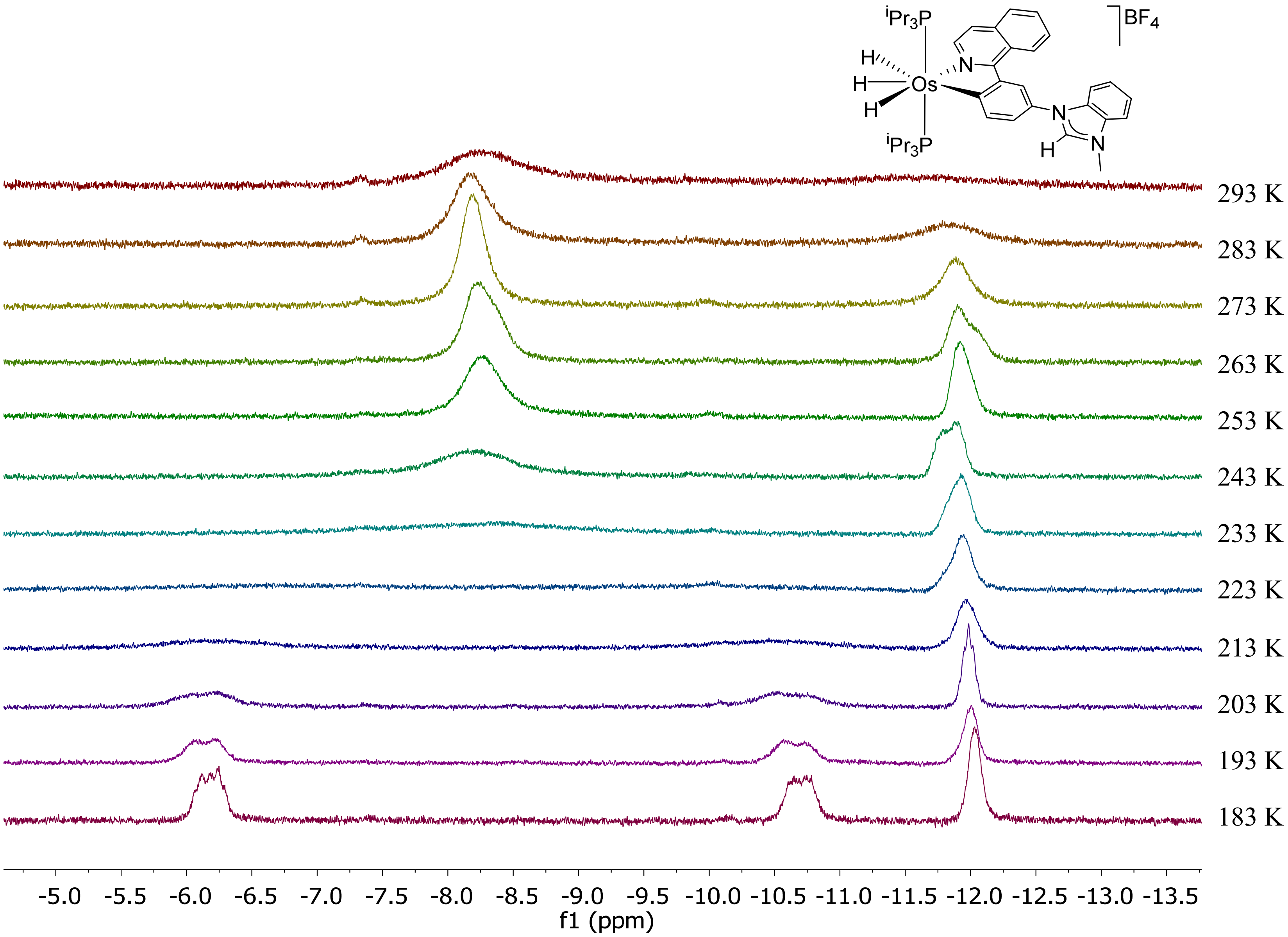

Figure S15. High field region of the ^{1}H NMR spectra (300.13 MHz, CD_2Cl_2) of compound **5** as a function of the temperature.

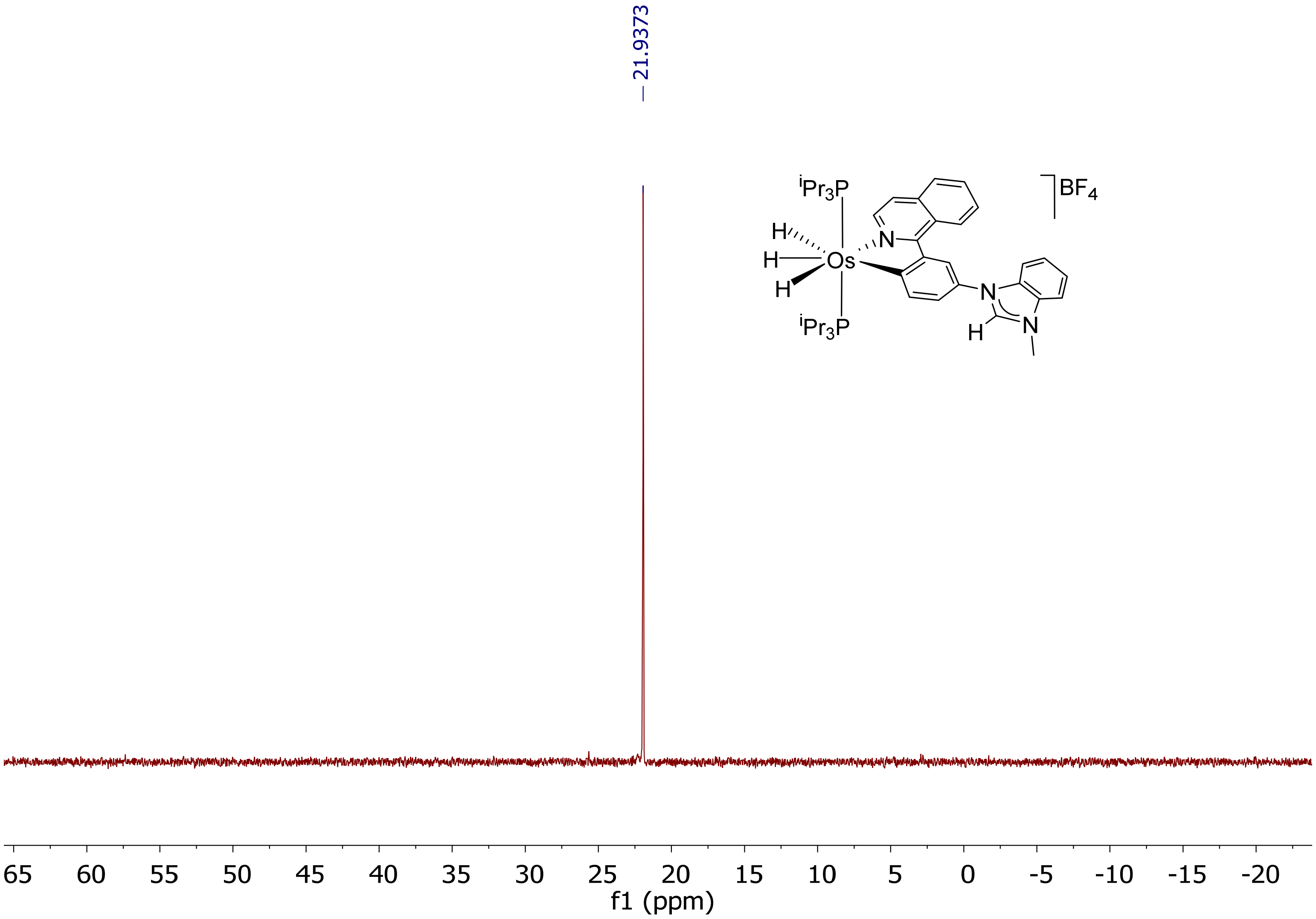

Figure S16. $^{31}P\{^{1}H\}$ NMR spectrum (121.49 MHz, CD_2Cl_2, 298 K) of compound **5**.

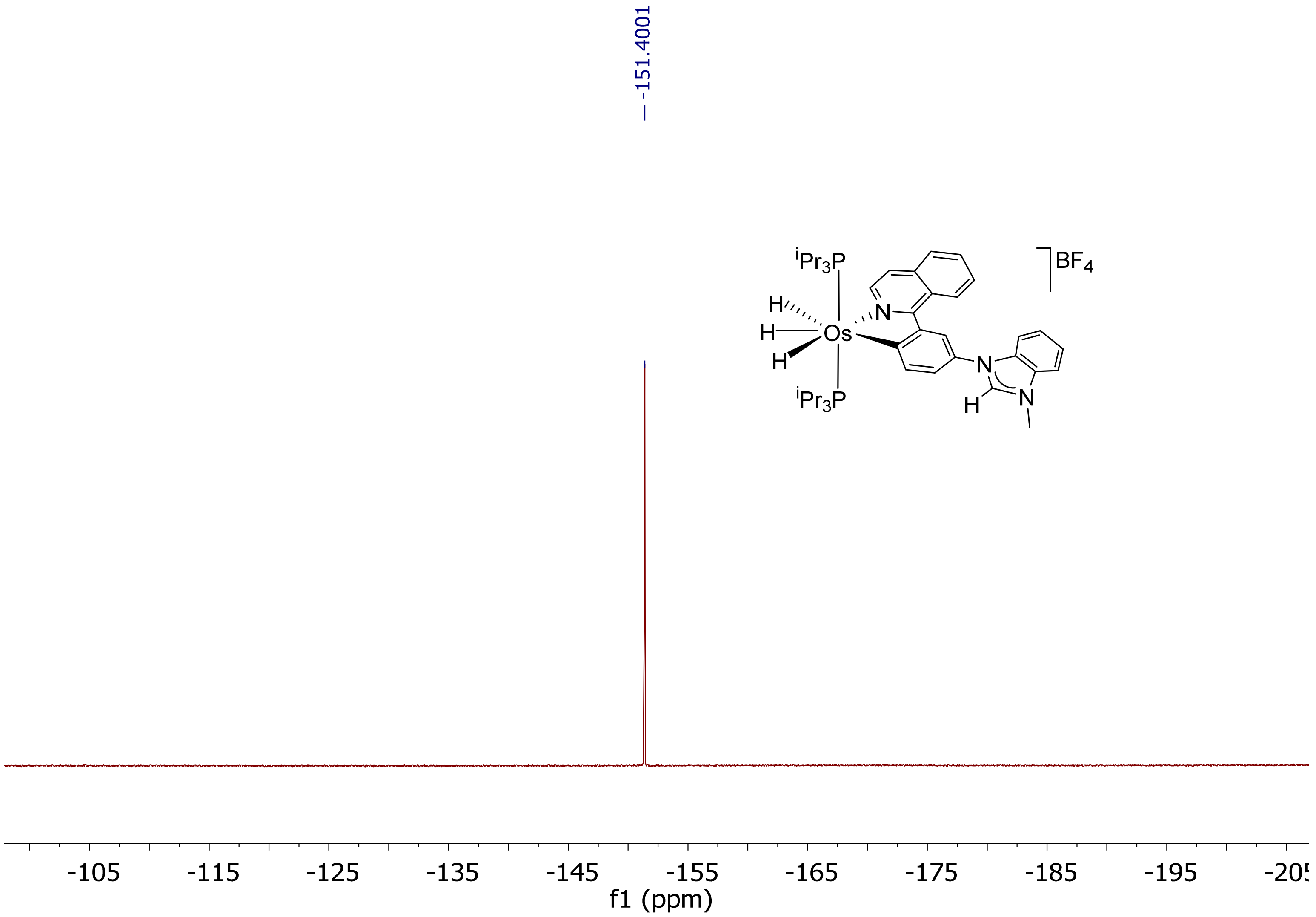

Figure S17. $^{19}F\{^{1}H\}$ NMR spectrum (282.38 MHz, CD_2Cl_2, 298 K) of compound **5**.

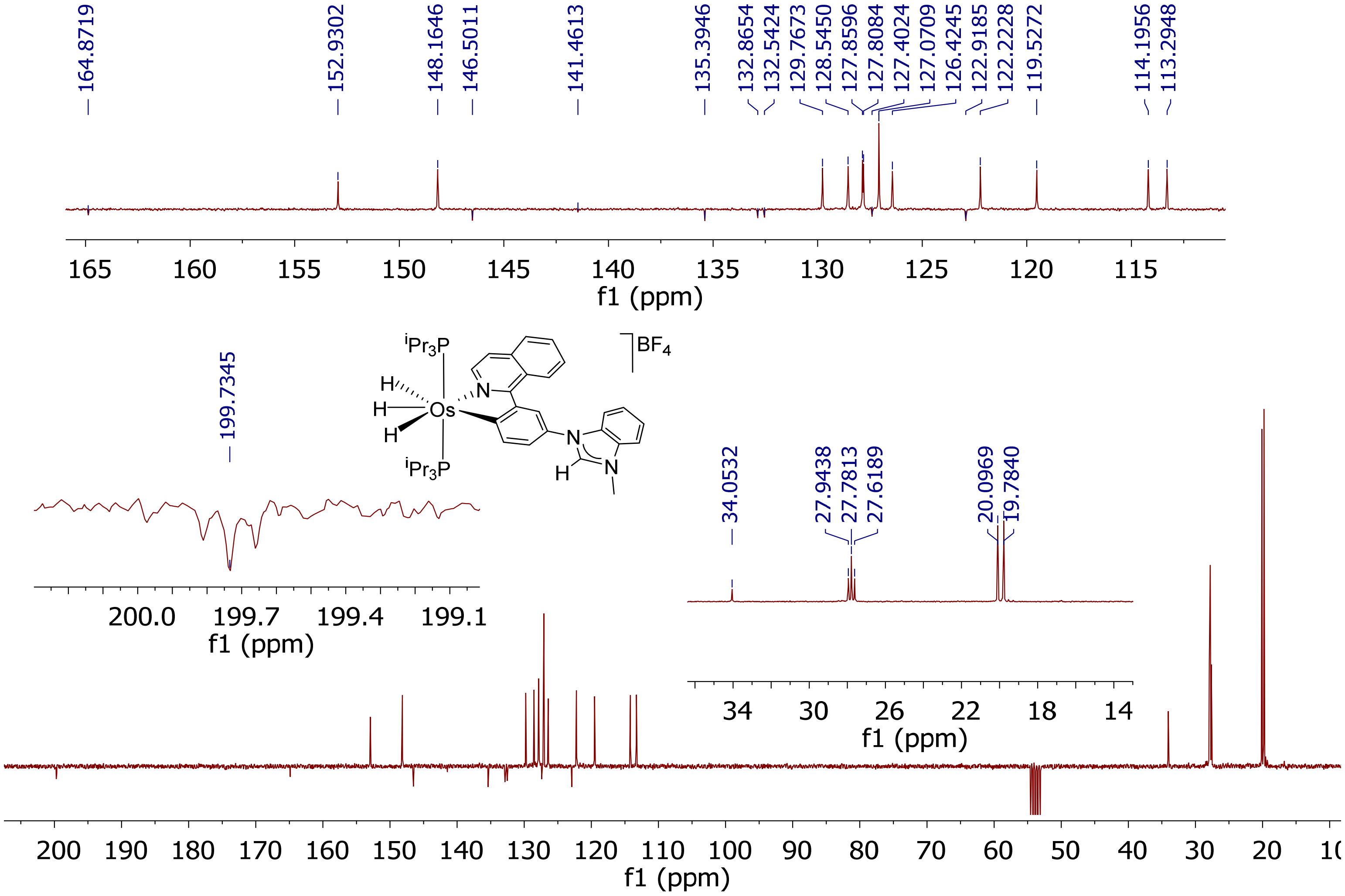

Figure S18. $^{13}C\{^{1}H\}$-apt NMR spectrum (75.48 MHz, CD_2Cl_2, 298 K) of compound **5**.

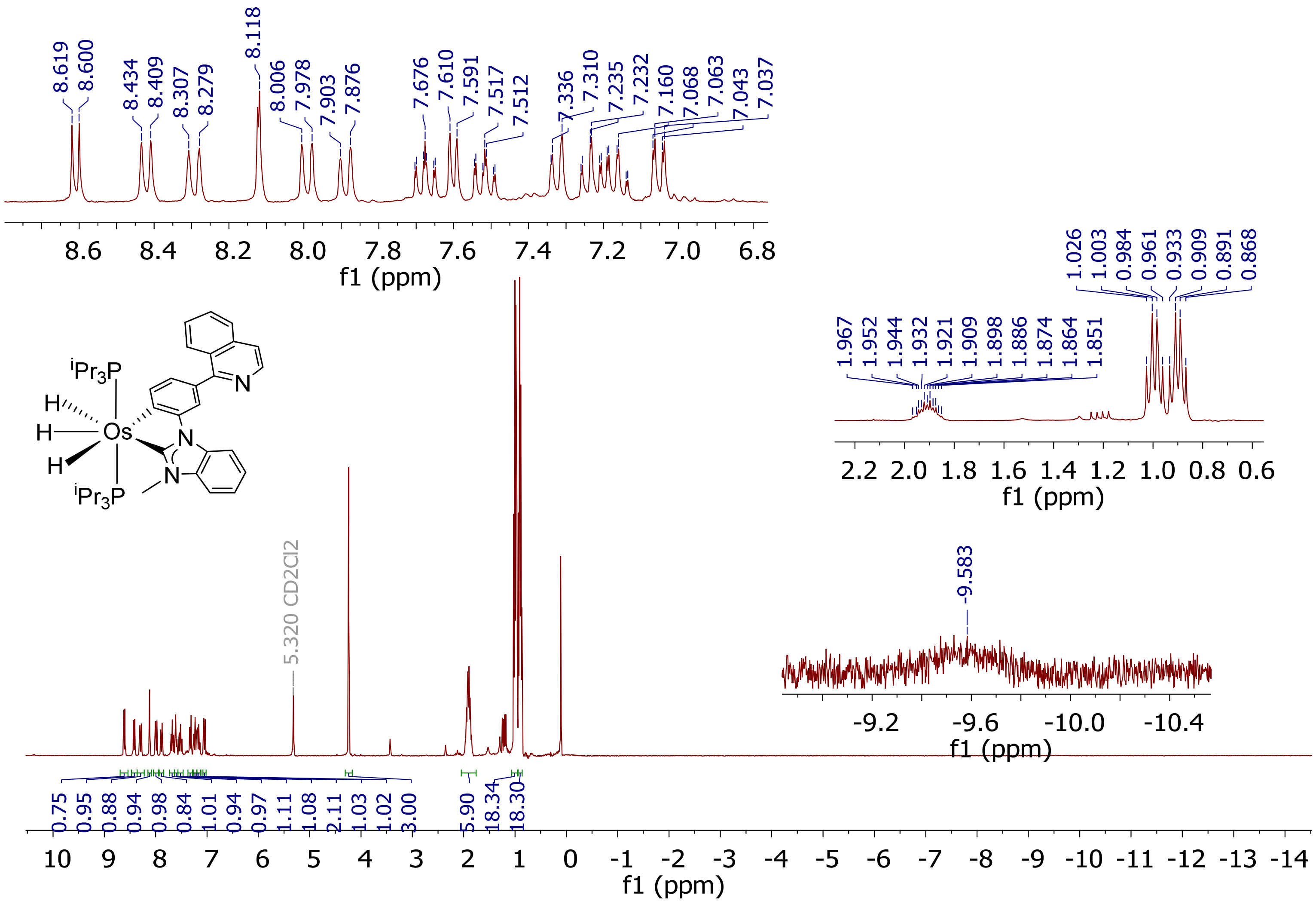

Figure S19. ^{1}H NMR spectrum (300.13 MHz, CD_2Cl_2, 298 K) of compound **6**.

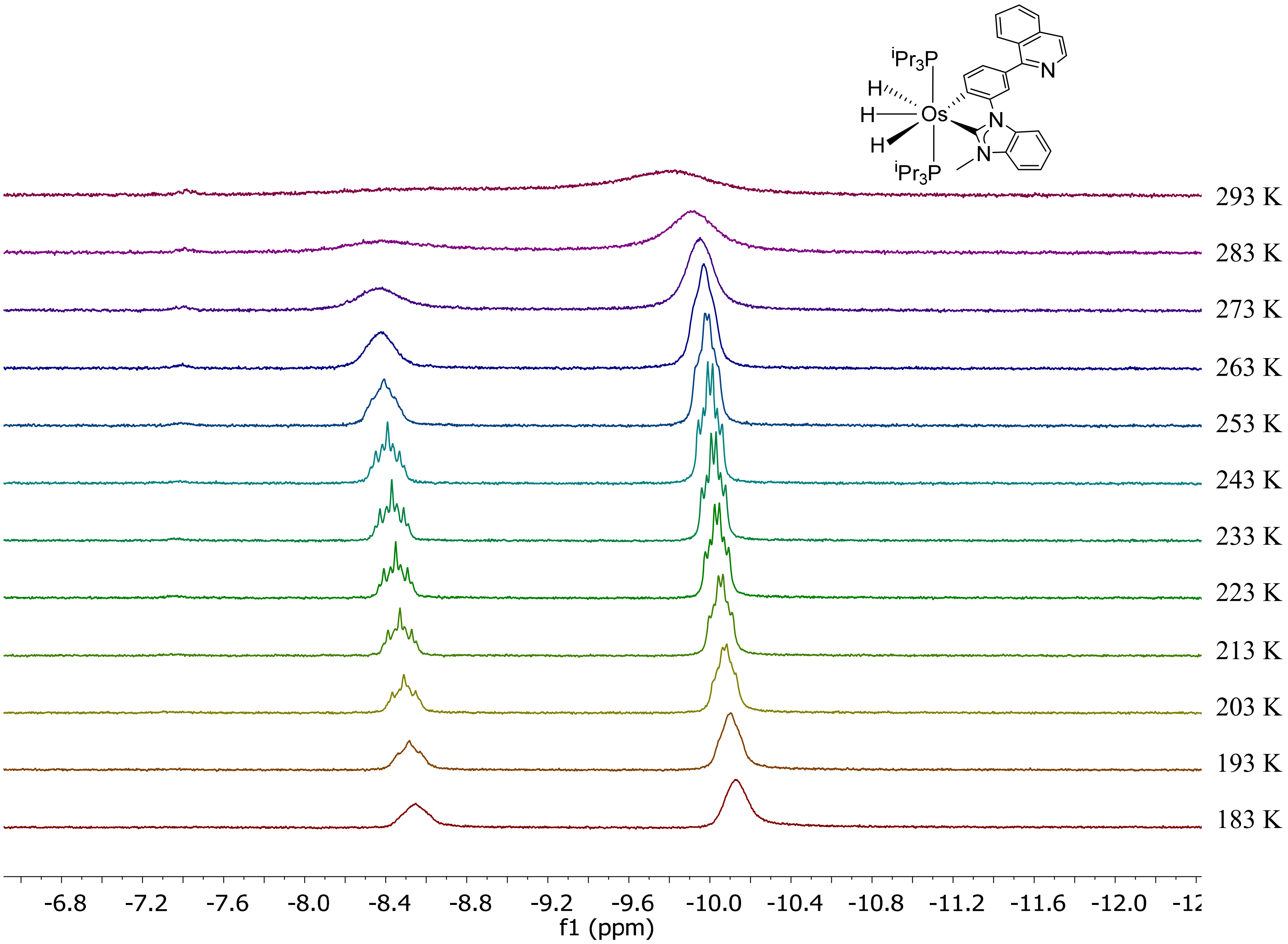

Figure S20. High field region of the ^{1}H NMR spectra (300.13 MHz, CD_2Cl_2) of compound **6** as a function of the temperature.

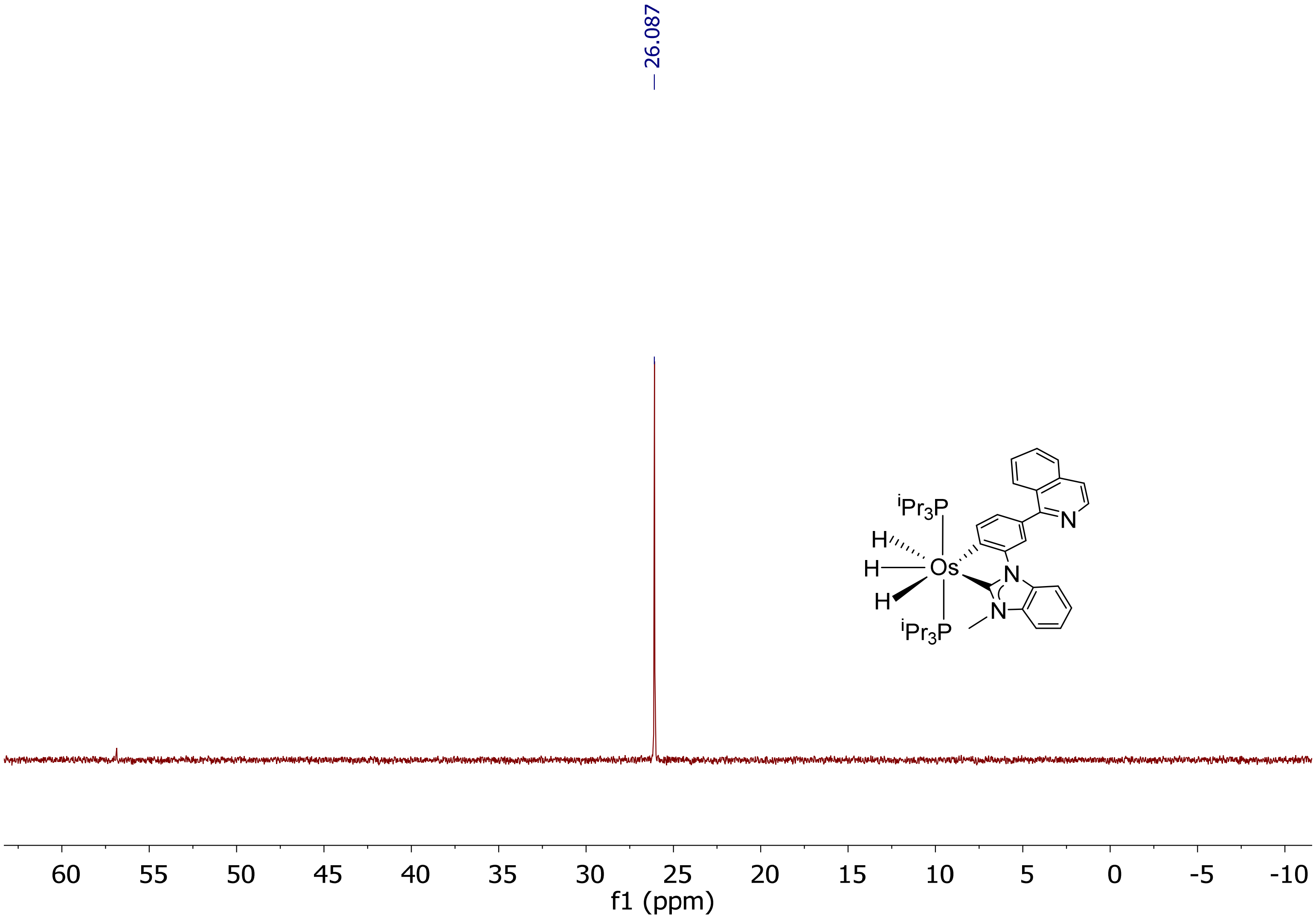

Figure S21. $^{31}P\{^{1}H\}$ NMR spectrum (121.49 MHz, CD_2Cl_2, 298 K) of compound **6**.

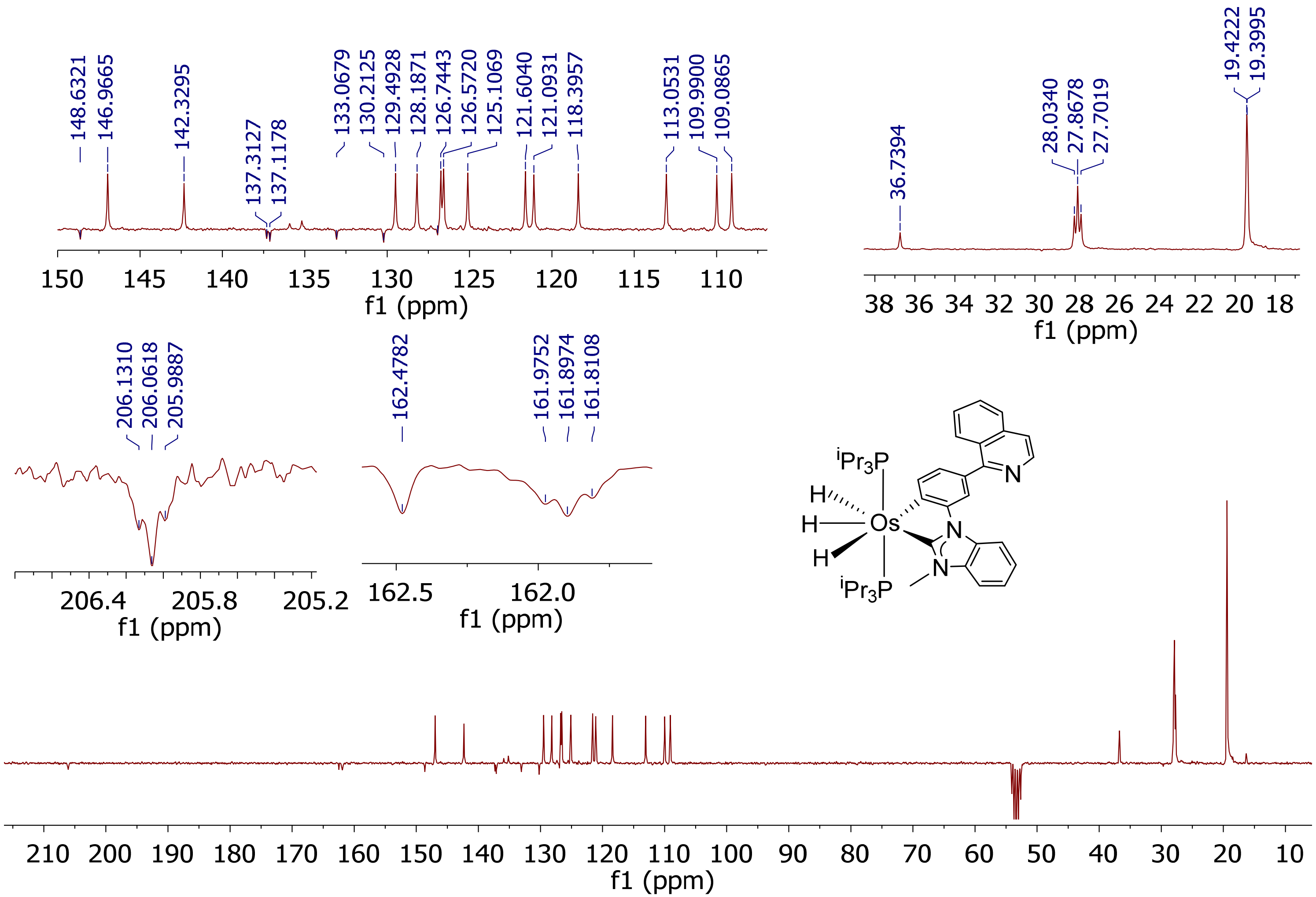

Figure S22. $^{13}C\{^{1}H\}$-apt NMR spectrum (75.48 MHz, CD_2Cl_2, 298 K) of compound **6**.

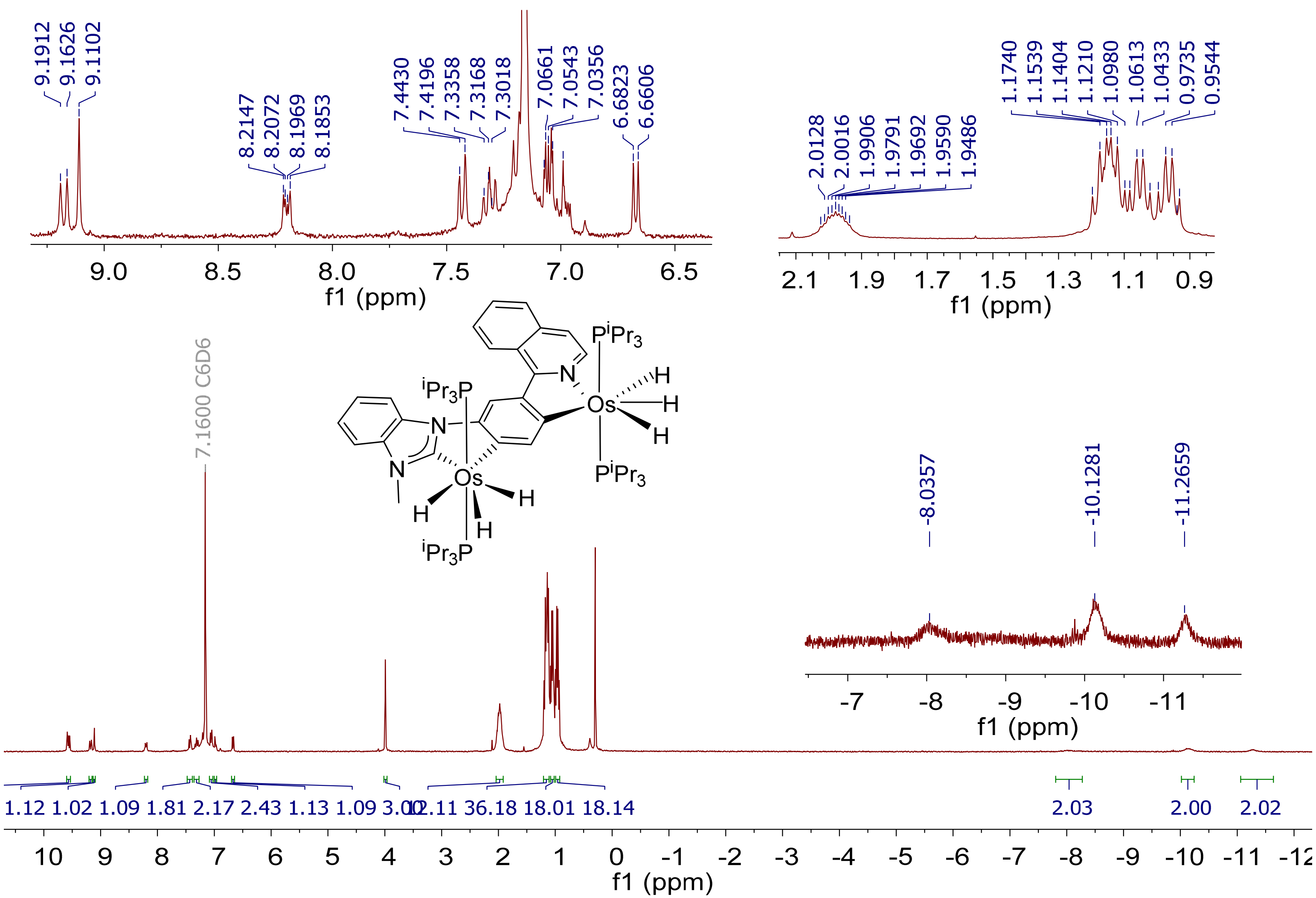

Figure S23. ^{1}H NMR spectrum (300.13 MHz, C_6D_6, 298 K) of compound **7**.

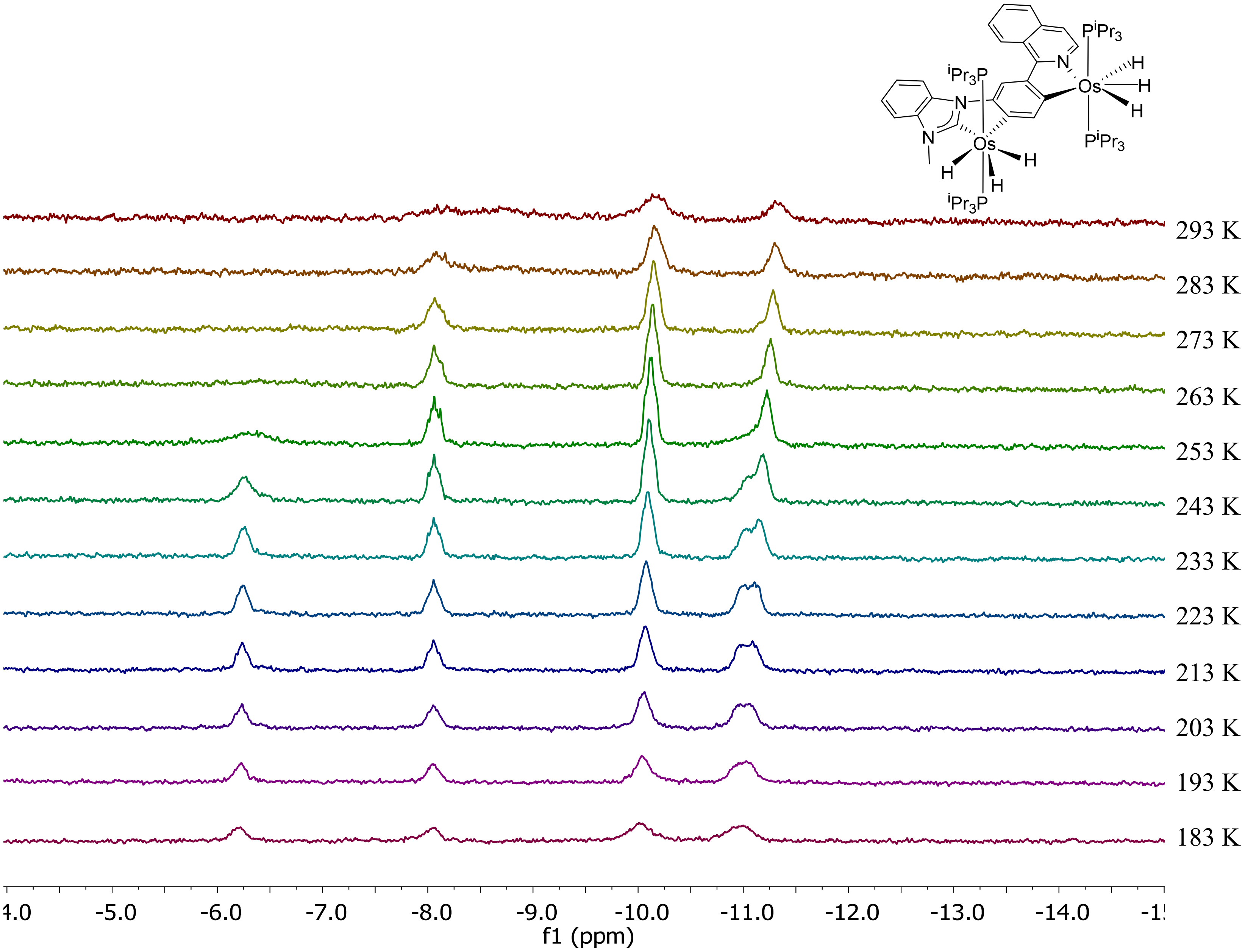

Figure S24. High field region of the ^{1}H NMR spectra (300.13 MHz, toluene-d_8) of compound **7** as a function of the temperature.

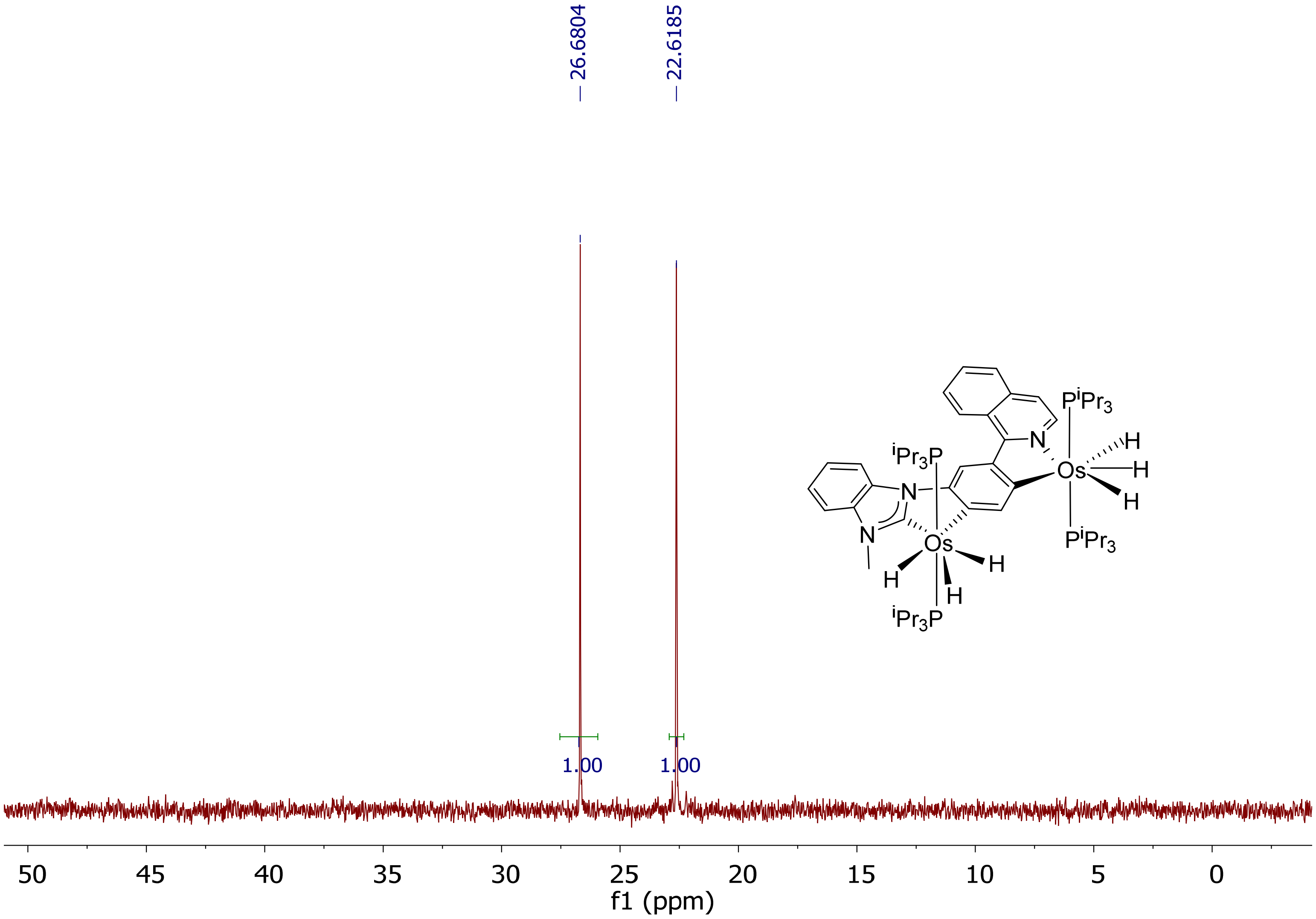

Figure S25. $^{31}P\{^{1}H\}$ NMR spectrum (121.49 MHz, C_6D_6, 298 K) of compound **7**.

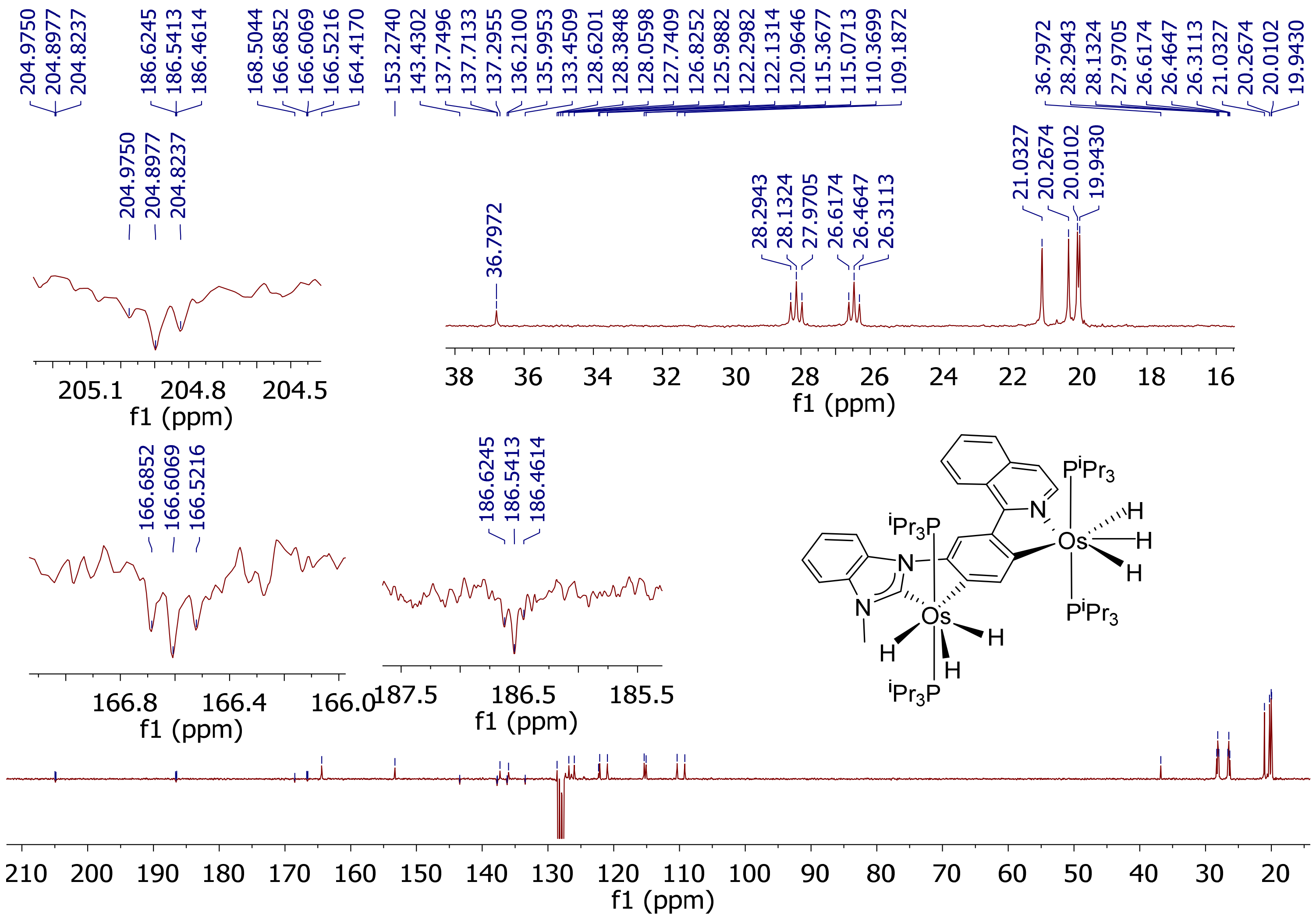

Figure S26. $^{13}C\{^{1}H\}$-apt NMR spectrum (75.48 MHz, C_6D_6, 298 K) of compound **7.**

^{1}H NMR Spectra of the reaction mixtures of the dehydrogenation of benzyl alcohol.

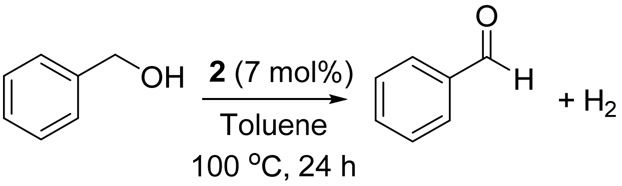

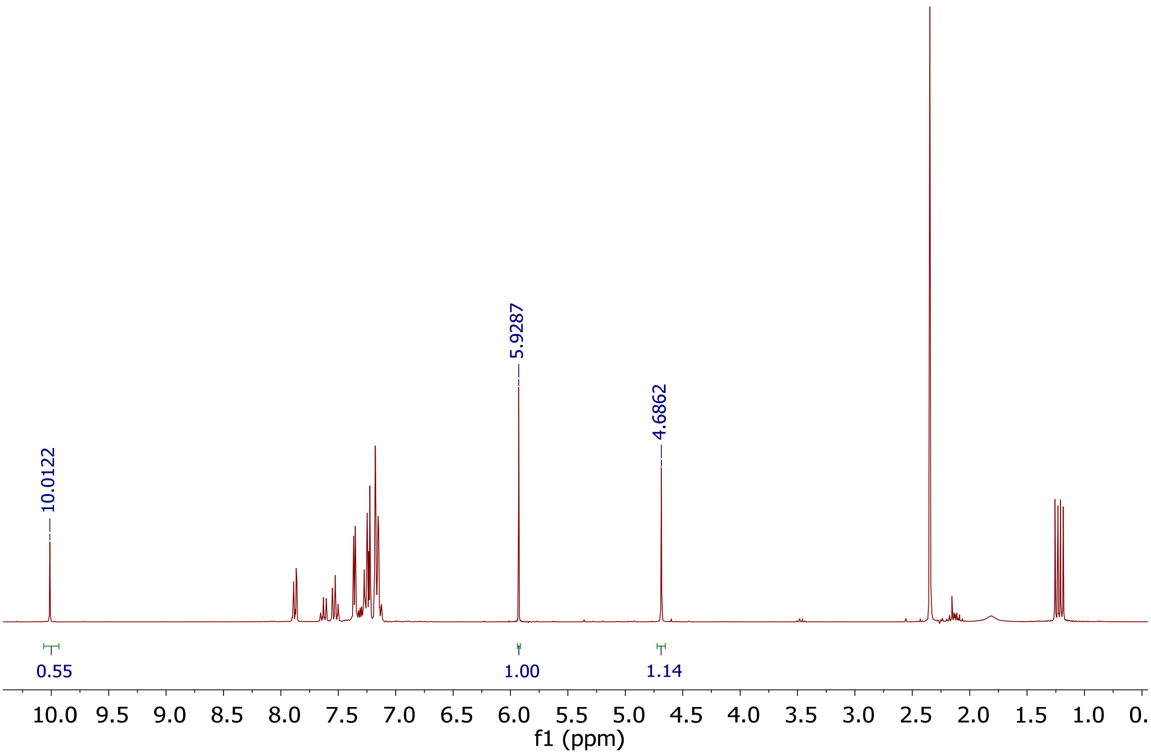

Figure S27. ^{1}H NMR spectrum (300 MHz, $CDCl_3$, 298 K) of the reaction mixture of the dehydrogenation of benzyl alcohol catalyzed by complex **2**. 1,1,2,2-Tetrachloroethane was used as internal standard. The conversion was determined by integration of characteristic NMR resonances of benzyl alcohol (δ 4.69, CH_2) and benzaldehyde (δ 10.01, CHO). A delay (d1) of 5 seconds was used in order to assure the correct integration of the resonances.

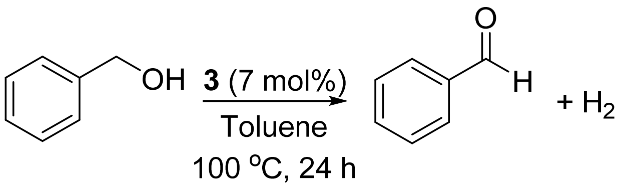

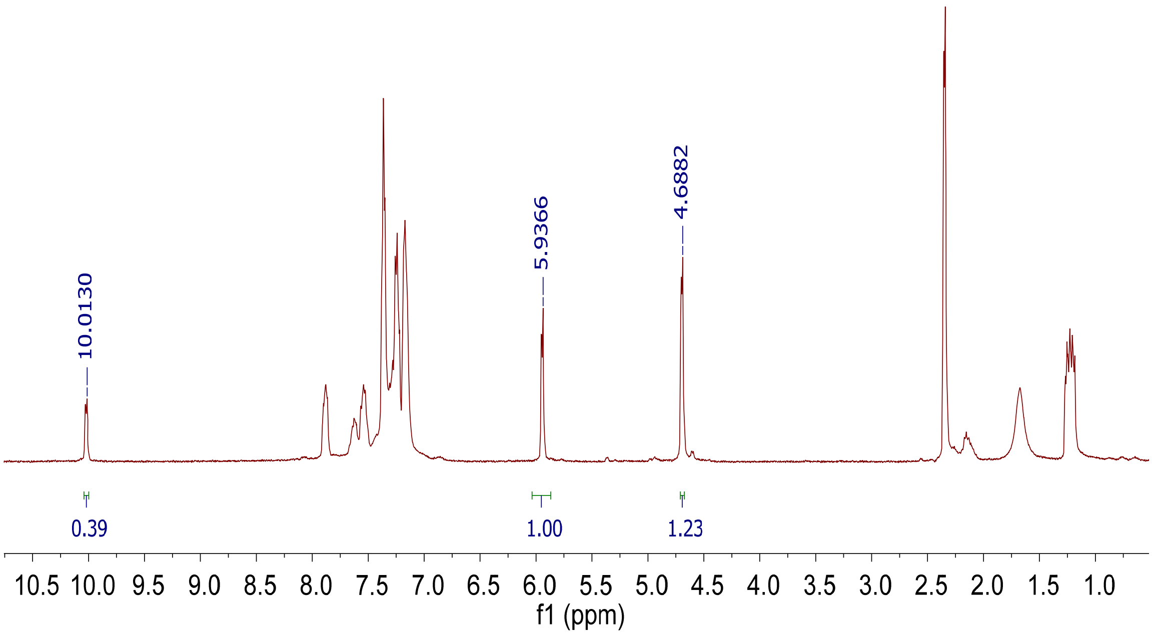

Figure S28. ^{1}H NMR spectrum (300 MHz, $CDCl_3$, 298 K) of the reaction mixture of the dehydrogenation of benzyl alcohol catalyzed by complex **3**. 1,1,2,2-Tetrachloroethane was used as internal standard. The conversion was determined by integration of characteristic NMR resonances of benzyl alcohol (δ 4.69, CH_2) and benzaldehyde (δ 10.01, CHO). A delay (d1) of 5 seconds was used in order to assure the correct integration of the resonances.

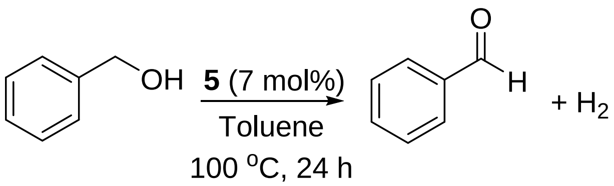

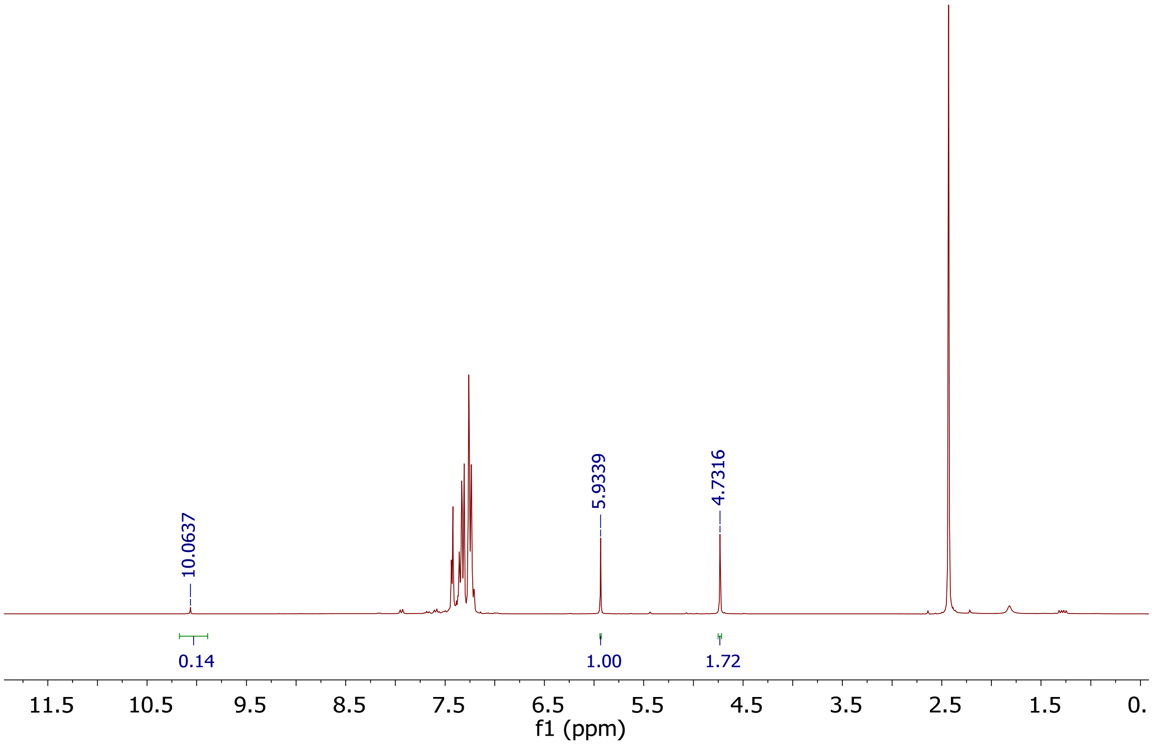

Figure S29. ^{1}H NMR spectrum (300 MHz, $CDCl_3$, 298 K) of the reaction mixture of the dehydrogenation of benzyl alcohol catalyzed by complex **5**. 1,1,2,2-Tetrachloroethane was used as internal standard. The conversion was determined by integration of characteristic NMR resonances of benzyl alcohol (δ 4.69, CH_2) and benzaldehyde (δ 10.01, CHO). A delay (d1) of 5 seconds was used in order to assure the correct integration of the resonances.

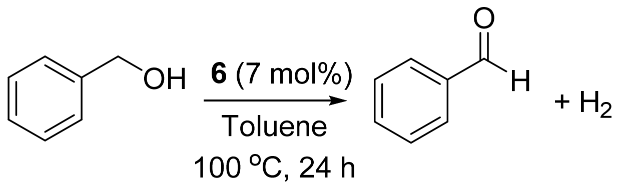

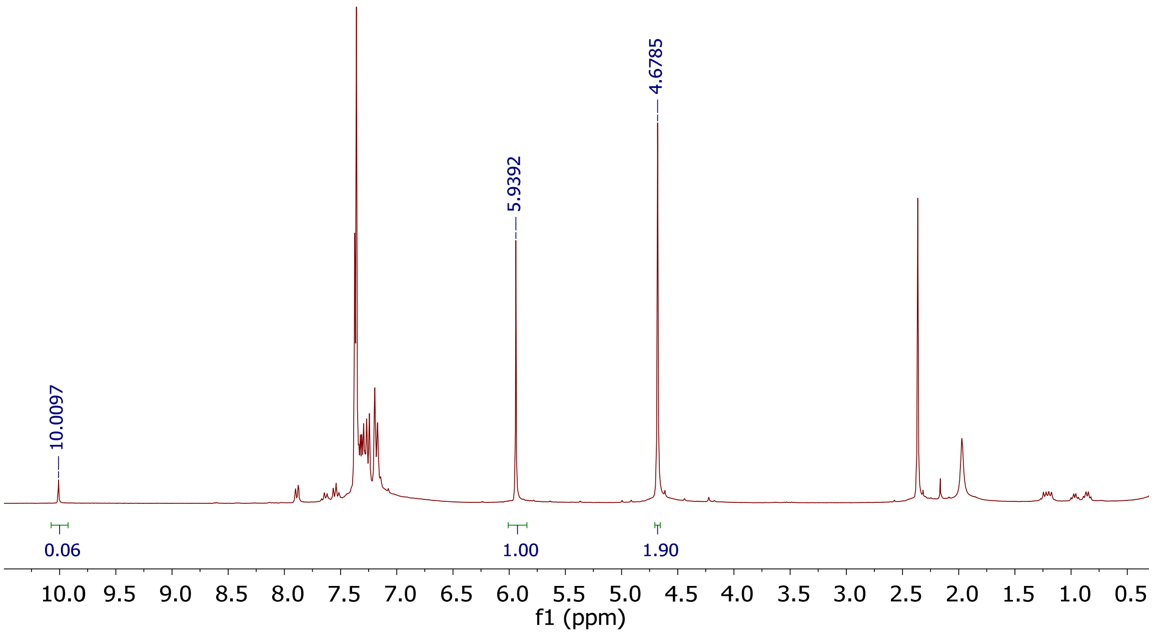

Figure S30. ^{1}H NMR spectrum (300 MHz, $CDCl_3$, 298 K) of the reaction mixture of the dehydrogenation of benzyl alcohol catalyzed by complex **6**. 1,1,2,2-Tetrachloroethane was used as internal standard. The conversion was determined by integration of characteristic NMR resonances of benzyl alcohol (δ 4.69, CH_2) and benzaldehyde (δ 10.01, CHO). A delay (d1) of 5 seconds was used in order to assure the correct integration of the resonances.

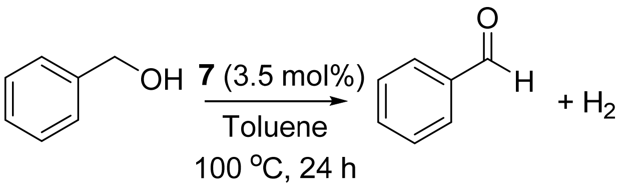

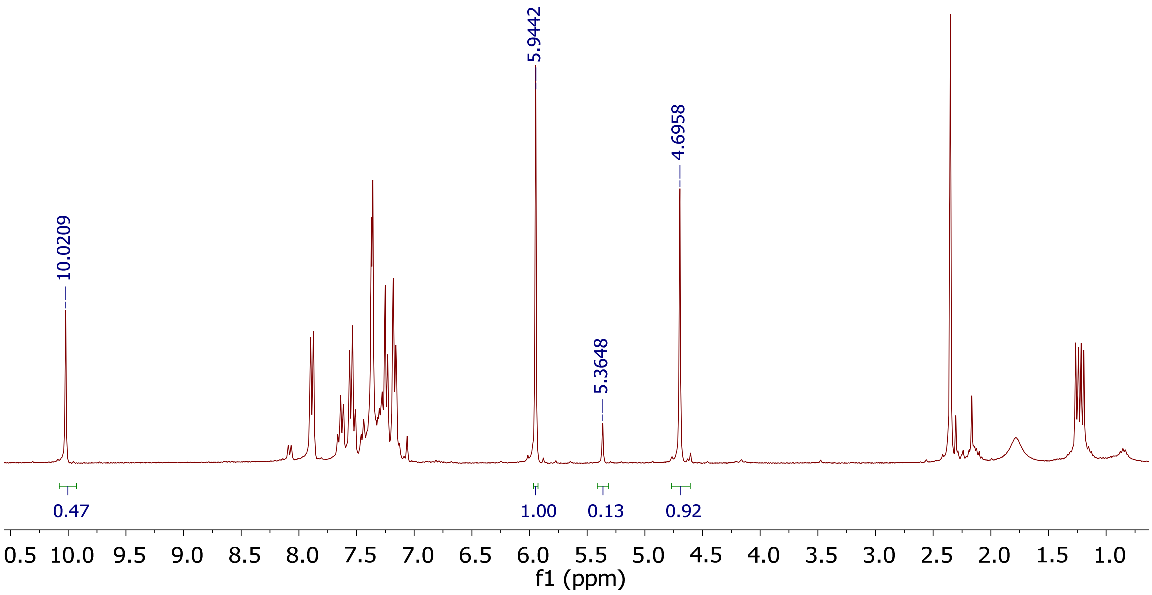

Figure S31. ^{1}H NMR spectrum (300 MHz, $CDCl_3$, 298 K) of the reaction mixture of the dehydrogenation of benzyl alcohol catalyzed by complex **7**. 1,1,2,2-Tetrachloroethane was used as internal standard. The conversion was determined by integration of characteristic NMR resonances of benzyl alcohol (δ 4.69, CH_2), benzaldehyde (δ 10.01, CHO) and benzyl benzoate (δ 5.36, OCH_2). A delay (d1) of 5 seconds was used in order to assure the correct integration of the resonances.

^{1}H NMR Spectra of the Reaction Mixtures of the Dehydrogenation of 1,2-Phenylenedimethanol

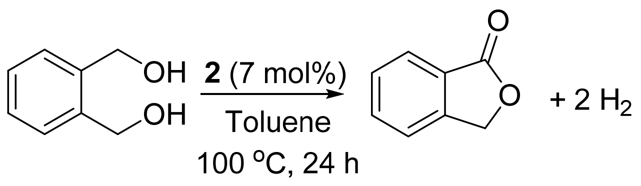

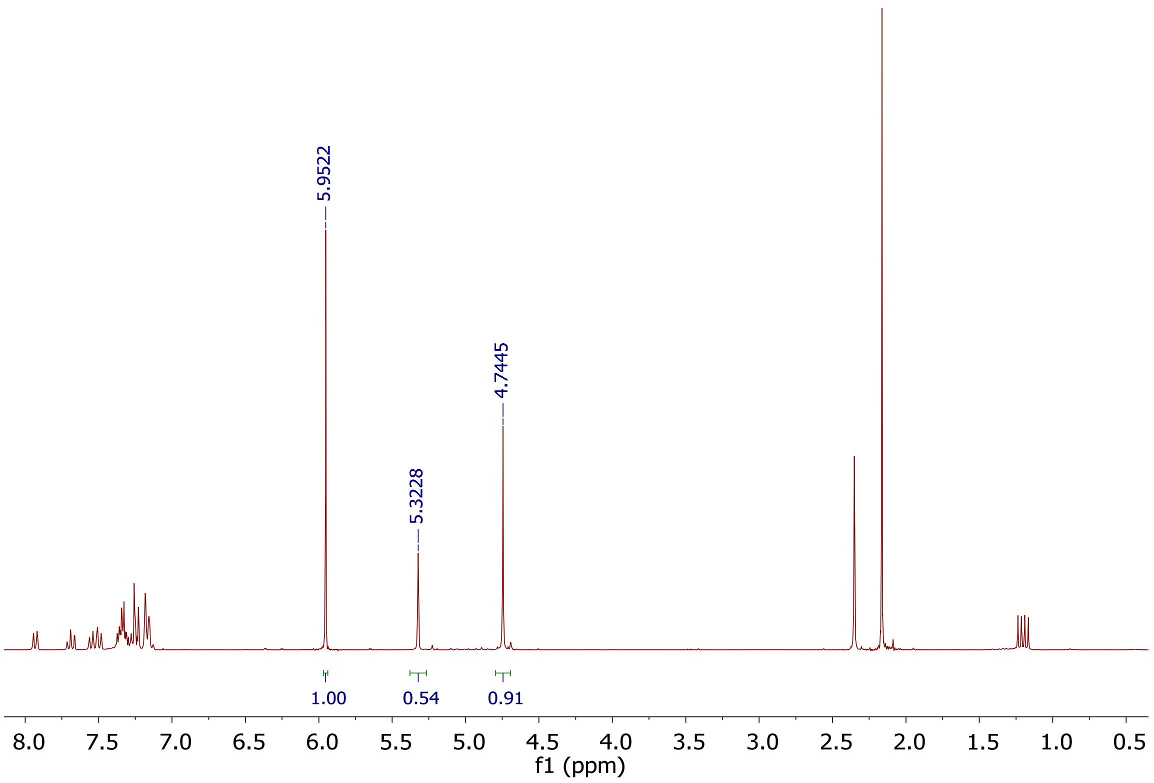

Figure S32. ^{1}H NMR spectrum (300 MHz, $CDCl_3$, 298 K) of the reaction mixture of the dehydrogenation of 1,2-phenylenedimethanol catalyzed by complex **2**. 1,1,2,2-tetrachloroethane used as internal standard. The conversion was determined by integration of characteristic NMR resonances of 1-isobenzofuranone (δ 5.32, OCH_2), and 1,2-phenylenedimethanol (δ 4.74, CH_2). A delay (d1) of 5 seconds was used in order to assure the correct integration of the resonances.

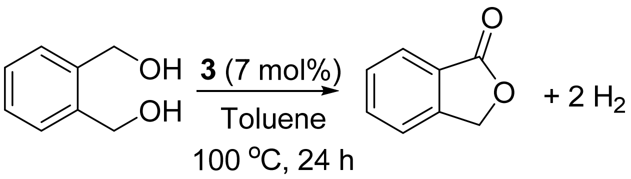

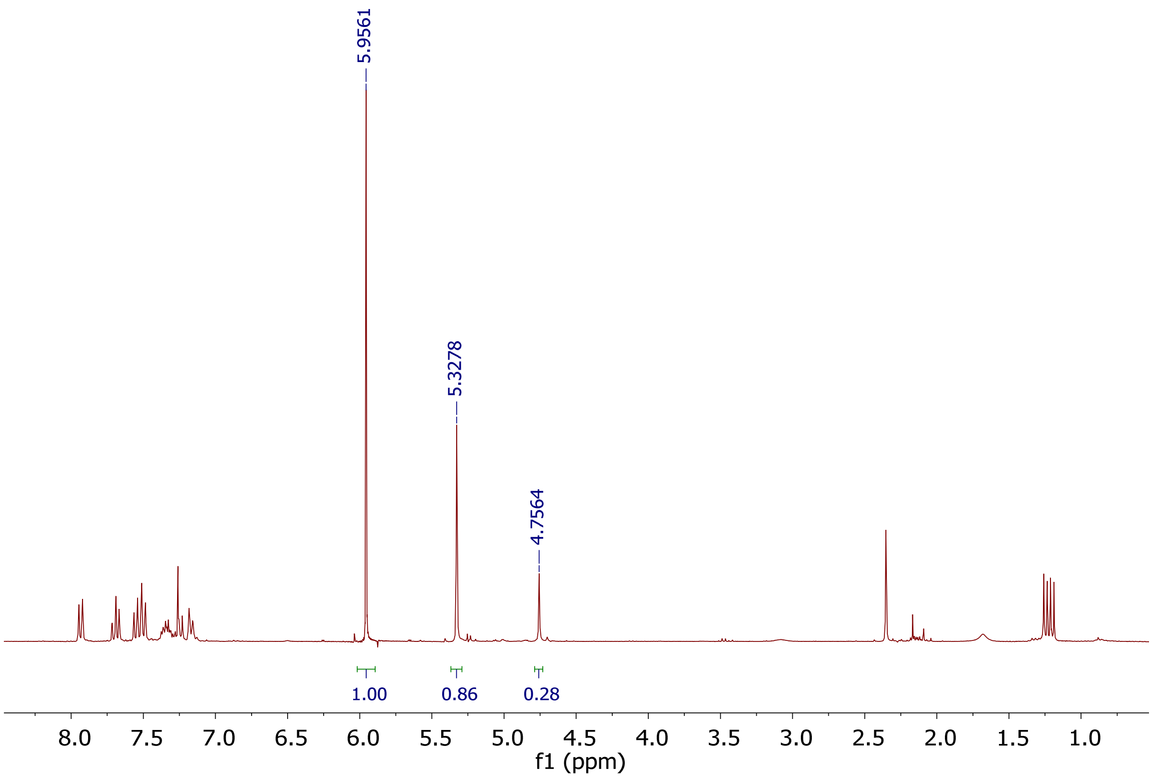

Figure S33. ^{1}H NMR spectrum (300 MHz, $CDCl_3$, 298 K) of the reaction mixture of the dehydrogenation of 1,2-phenylenedimethanol catalyzed by complex **3**. 1,1,2,2-Tetrachloroethane used as internal standard. The conversion was determined by integration of characteristic NMR resonances of 1-isobenzofuranone (δ 5.32, OCH_2), and 1,2-phenylenedimethanol (δ 4.74, CH_2). A delay (d1) of 5 seconds was used in order to assure the correct integration of the resonances.

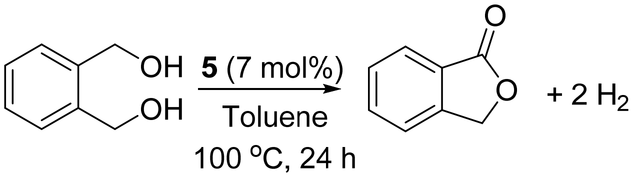

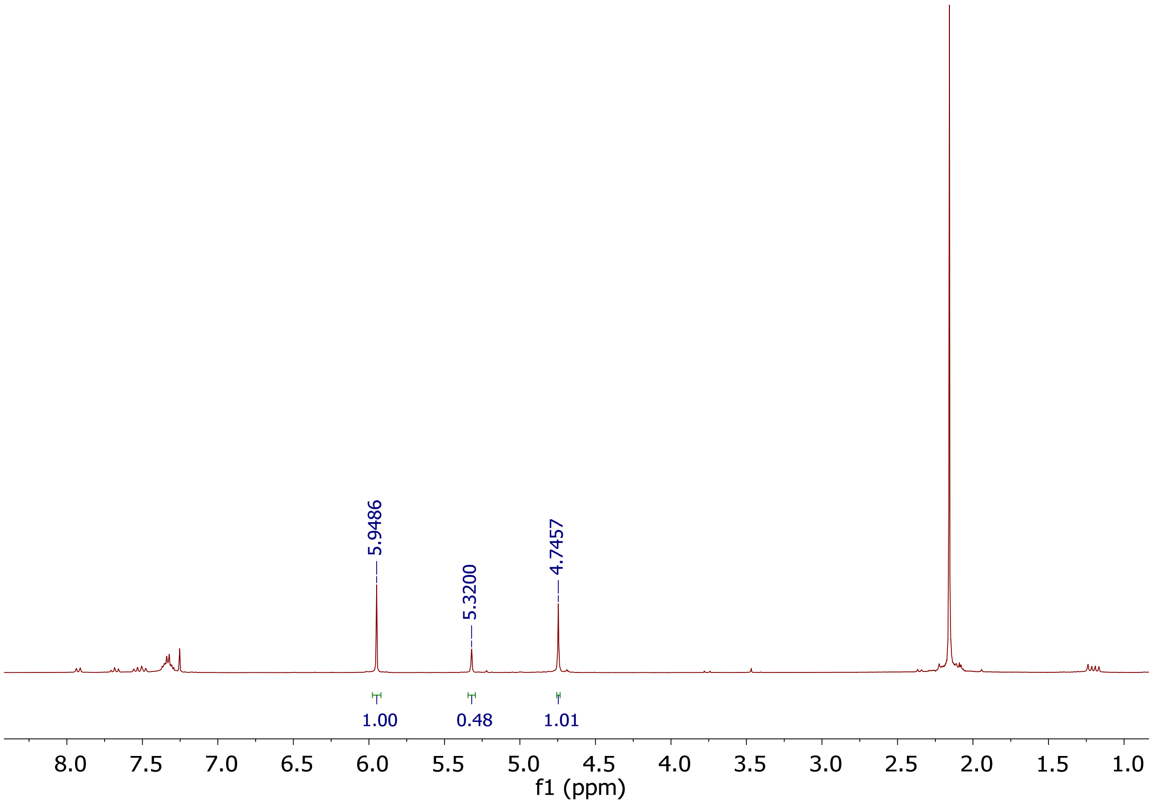

Figure S34. ^1H NMR spectrum (300 MHz, $CDCl_3$, 298 K) of the reaction mixture of the dehydrogenation of 1,2-phenylenedimethanol catalyzed by complex **5**. 1,1,2,2-Tetrachloroethane used as internal standard. The conversion was determined by integration of characteristic NMR resonances of 1-isobenzofuranone (δ 5.32, OCH_2), and 1,2-phenylenedimethanol (δ 4.74, CH_2). A delay (d1) of 5 seconds was used in order to assure the correct integration of the resonances.

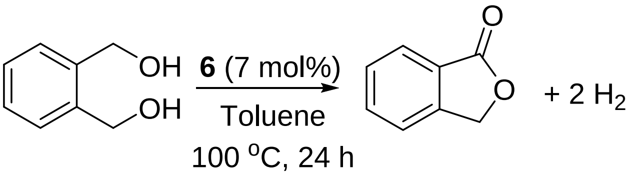

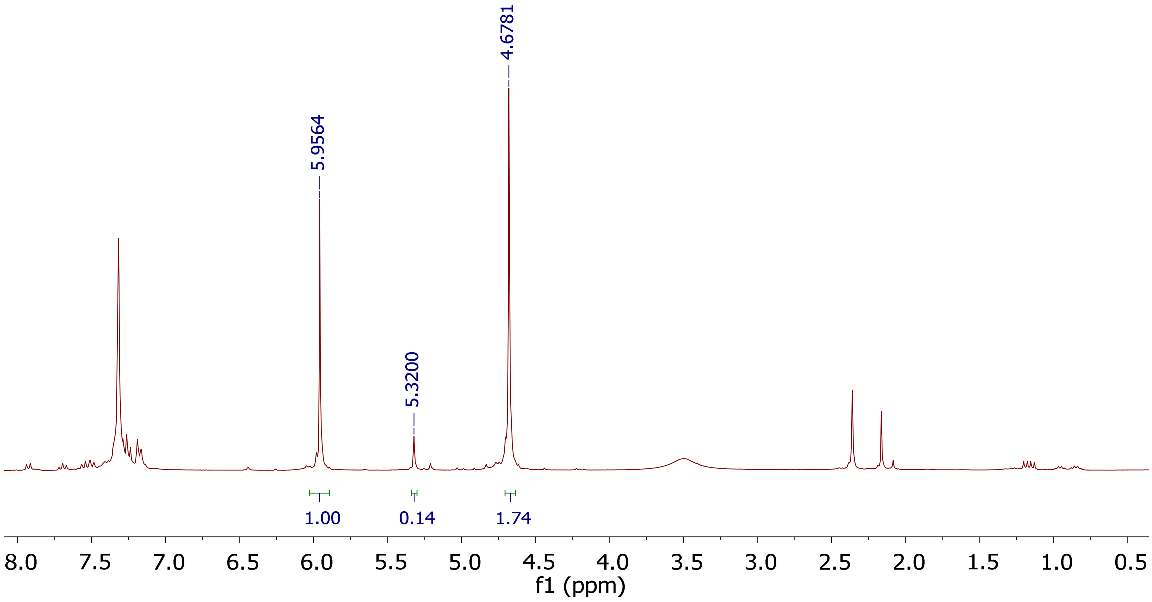

Figure S35. ^1H NMR spectrum (300 MHz, $CDCl_3$, 298 K) of the reaction mixture of the dehydrogenation of 1,2-phenylenedimethanol catalyzed by complex **6**. 1,1,2,2-Tetrachloroethane used as internal standard. The conversion was determined by integration of characteristic NMR resonances of 1-isobenzofuranone (δ 5.32, OCH_2), and 1,2-phenylenedimethanol (δ 4.74, CH_2). A delay (d1) of 5 seconds was used in order to assure the correct integration of the resonances.

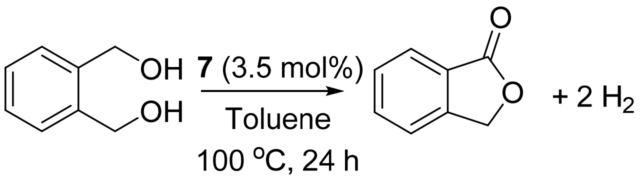

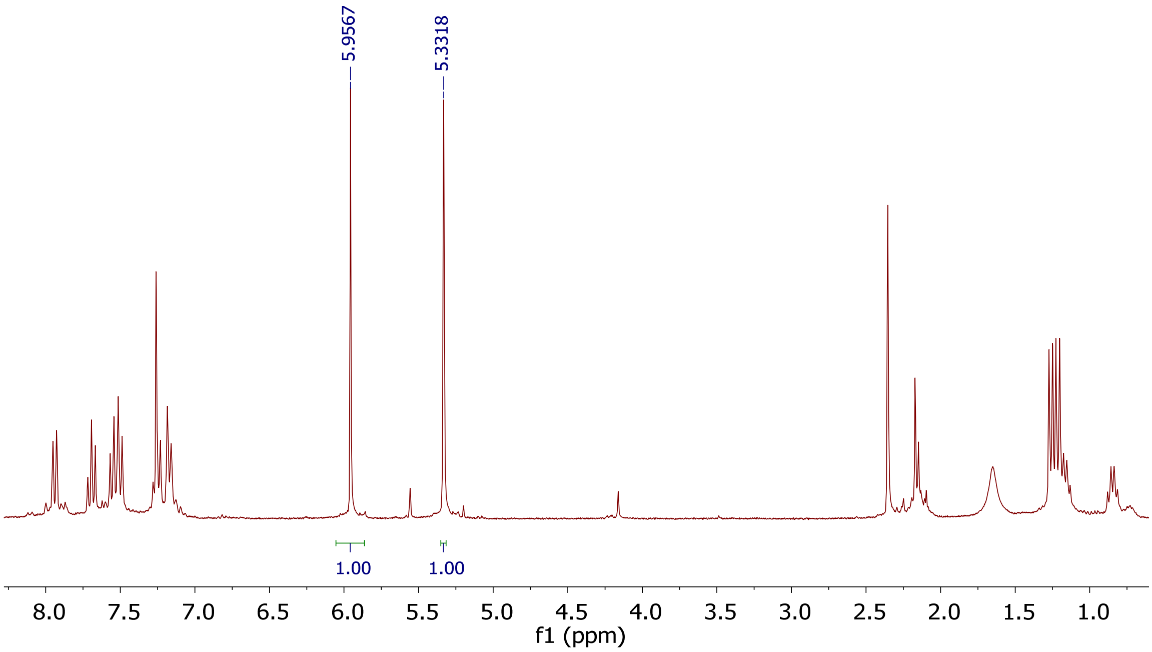

Figure S36. ^{1}H NMR spectrum (300 MHz, $CDCl_3$, 298 K) of the reaction mixture of the dehydrogenation of 1,2-phenylenedimethanol catalyzed by complex **7**. 1,1,2,2-Tetrachloroethane used as internal standard. The conversion was determined by integration of characteristic NMR resonance of 1-isobenzofuranone (δ 5.32, OCH_2). A delay (d1) of 5 seconds was used in order to assure the correct integration of the resonances.

● **Computational Details**

All calculations were performed at the DFT level using the B3LYP functional[4] supplemented with the Grimme's dispersion correction D3[5] as implemented in Gaussian09.[6] Os atoms were described by means of an effective core potential SDD for the inner electron[7] and its associated double-ζ basis set for the outer ones, complemented with a set of f-polarization functions for osmium.[8] The 6-31G** basis set was used for the H, C, N, and P.[9] All minima were verified to have no negative frequencies. The geometries were fully optimized in in THF (ε = 7.4257) and dichloromethane (ε = 8.93) solvents using the continuum SMD model.[10] We performed TD-DFT calculations at the same level of theory in THF or CH_2Cl_2 calculating the lowest 50 excitations. The UV/vis absorption spectra were obtained by using the GaussSum 3 software.[11] The phosphorescence emission compares well with the 0-0 transition calculated taking into account the zero point energies (zpe) of the geometries of both the optimized S_0 and T_1 states in THF.

● **Energies of the Optimized Structures**

Complex 2 (THF)

Zero-point correction=	0.900819 (Hartree/Particle)
Thermal correction to Energy=	0.950501
Thermal correction to Enthalpy=	0.951445
Thermal correction to Gibbs Free Energy=	0.819614
Sum of electronic and zero-point Energies=	-2383.091837
Sum of electronic and thermal Energies=	-2383.042155
Sum of electronic and thermal Enthalpies=	-2383.041211
Sum of electronic and thermal Free Energies=	-2383.173041

Complex 2 (CH_2Cl_2)

Zero-point correction=	0.900522 (Hartree/Particle)
Thermal correction to Energy=	0.950250
Thermal correction to Enthalpy=	0.951195
Thermal correction to Gibbs Free Energy=	0.819207
Sum of electronic and zero-point Energies=	-2383.100895
Sum of electronic and thermal Energies=	-2383.051166
Sum of electronic and thermal Enthalpies=	-2383.050222
Sum of electronic and thermal Free Energies=	-2383.182210

Complex 3 (THF)

Zero-point correction=	0.882109 (Hartree/Particle)
Thermal correction to Energy=	0.930484
Thermal correction to Enthalpy=	0.931428
Thermal correction to Gibbs Free Energy=	0.804543
Sum of electronic and zero-point Energies=	-2381.935276
Sum of electronic and thermal Energies=	-2381.886902
Sum of electronic and thermal Enthalpies=	-2381.885958
Sum of electronic and thermal Free Energies=	-2382.012843

Complex 3 (CH_2Cl_2)

Zero-point correction=	0.881790 (Hartree/Particle)
Thermal correction to Energy=	0.930230
Thermal correction to Enthalpy=	0.931174
Thermal correction to Gibbs Free Energy=	0.804041
Sum of electronic and zero-point Energies=	-2381.943496
Sum of electronic and thermal Energies=	-2381.895056
Sum of electronic and thermal Enthalpies=	-2381.894112
Sum of electronic and thermal Free Energies=	-2382.021245

Complex 4 (THF)

Zero-point correction= 0.887221 (Hartree/Particle)
Thermal correction to Energy= 0.936538
Thermal correction to Enthalpy= 0.937482
Thermal correction to Gibbs Free Energy= 0.806897
Sum of electronic and zero-point Energies= -2382.633973
Sum of electronic and thermal Energies= -2382.584656
Sum of electronic and thermal Enthalpies= -2382.583711
Sum of electronic and thermal Free Energies= -2382.714296

Complex 4 (CH_2Cl_2)

Zero-point correction= 0.886906 (Hartree/Particle)
Thermal correction to Energy= 0.936294
Thermal correction to Enthalpy= 0.937238
Thermal correction to Gibbs Free Energy= 0.806314
Sum of electronic and zero-point Energies= -2382.642232
Sum of electronic and thermal Energies= -2382.592844
Sum of electronic and thermal Enthalpies= -2382.591900
Sum of electronic and thermal Free Energies= -2382.722824

Complex 5 (THF)

Zero-point correction= 0.947773 (Hartree/Particle)
Thermal correction to Energy= 1.000061
Thermal correction to Enthalpy= 1.001005
Thermal correction to Gibbs Free Energy= 0.864158
Sum of electronic and zero-point Energies= -2536.706861
Sum of electronic and thermal Energies= -2536.654574
Sum of electronic and thermal Enthalpies= -2536.653629
Sum of electronic and thermal Free Energies= -2536.790476

Complex 5 (CH_2Cl_2)

Zero-point correction= 0.947407 (Hartree/Particle)
Thermal correction to Energy= 0.999770
Thermal correction to Enthalpy= 1.000714
Thermal correction to Gibbs Free Energy= 0.863541
Sum of electronic and zero-point Energies= -2536.716769
Sum of electronic and thermal Energies= -2536.664406
Sum of electronic and thermal Enthalpies= -2536.663461
Sum of electronic and thermal Free Energies= -2536.800634

Complex 6 (THF)

Zero-point correction= 0.934413 (Hartree/Particle)
Thermal correction to Energy= 0.986462
Thermal correction to Enthalpy= 0.987407
Thermal correction to Gibbs Free Energy= 0.850563
Sum of electronic and zero-point Energies= -2536.252666
Sum of electronic and thermal Energies= -2536.200616
Sum of electronic and thermal Enthalpies= -2536.199672
Sum of electronic and thermal Free Energies= -2536.336516

Complex 6 (CH_2Cl_2)

Zero-point correction= 0.934055 (Hartree/Particle)
Thermal correction to Energy= 0.986166
Thermal correction to Enthalpy= 0.987110
Thermal correction to Gibbs Free Energy= 0.850003
Sum of electronic and zero-point Energies= -2536.261835
Sum of electronic and thermal Energies= -2536.209724
Sum of electronic and thermal Enthalpies= -2536.208780
Sum of electronic and thermal Free Energies= -2536.345888

Complex 7 (THF)

Zero-point correction= 1.527321 (Hartree/Particle)
Thermal correction to Energy= 1.611562
Thermal correction to Enthalpy= 1.612506
Thermal correction to Gibbs Free Energy= 1.414036
Sum of electronic and zero-point Energies= -4021.798559
Sum of electronic and thermal Energies= -4021.714317
Sum of electronic and thermal Enthalpies= -4021.713373
Sum of electronic and thermal Free Energies= -4021.911844

Complex 7 (CH_2Cl_2)

Zero-point correction= 1.526783 (Hartree/Particle)
Thermal correction to Energy= 1.611115
Thermal correction to Enthalpy= 1.612059
Thermal correction to Gibbs Free Energy= 1.413281
Sum of electronic and zero-point Energies= -4021.809981
Sum of electronic and thermal Energies= -4021.725649
Sum of electronic and thermal Enthalpies= -4021.724705
Sum of electronic and thermal Free Energies= -4021.923483

Complex $[7]^+$ cationic (CH_2Cl_2)

Zero-point correction= 1.527906 (Hartree/Particle)
Thermal correction to Energy= 1.612255
Thermal correction to Enthalpy= 1.613199
Thermal correction to Gibbs Free Energy= 1.413573
Sum of electronic and zero-point Energies= -4021.662882
Sum of electronic and thermal Energies= -4021.578533
Sum of electronic and thermal Enthalpies= -4021.577589
Sum of electronic and thermal Free Energies= -4021.777215

Complex $[7]^{2+}$ dicationic (CH_2Cl_2)

Zero-point correction= 1.534799 (Hartree/Particle)
Thermal correction to Energy= 1.617535
Thermal correction to Enthalpy= 1.618479
Thermal correction to Gibbs Free Energy= 1.425465
Sum of electronic and zero-point Energies= -4021.496809
Sum of electronic and thermal Energies= -4021.414073
Sum of electronic and thermal Enthalpies= -4021.413129
Sum of electronic and thermal Free Energies= -4021.606143

Complex $[7]^{3+}$ tricationic (CH_2Cl_2)

Zero-point correction= 1.539808 (Hartree/Particle)
Thermal correction to Energy= 1.621787
Thermal correction to Enthalpy= 1.622731
Thermal correction to Gibbs Free Energy= 1.429940
Sum of electronic and zero-point Energies= -4021.309350
Sum of electronic and thermal Energies= -4021.227371
Sum of electronic and thermal Enthalpies= -4021.226427
Sum of electronic and thermal Free Energies= -4021.419218

Complex $[7]^{4+}$ tetracationic (CH_2Cl_2)

Zero-point correction= 1.552206 (Hartree/Particle)
Thermal correction to Energy= 1.631382
Thermal correction to Enthalpy= 1.632326
Thermal correction to Gibbs Free Energy= 1.448720
Sum of electronic and zero-point Energies= -4021.109195
Sum of electronic and thermal Energies= -4021.030019
Sum of electronic and thermal Enthalpies= -4021.029075
Sum of electronic and thermal Free Energies= -4021.212680

● UV-vis Spectrum of Complex 7 (Observed and Calculated)

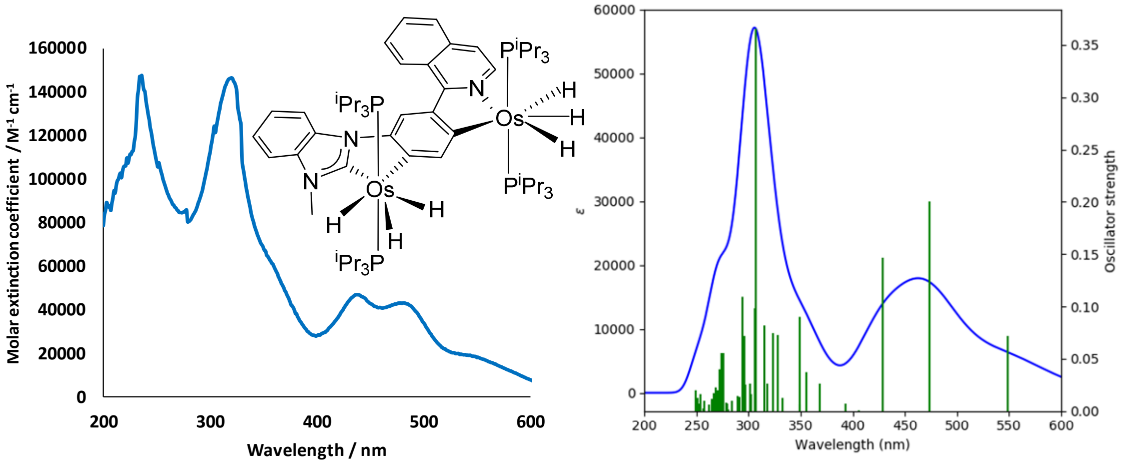

Figure S37. Observed UV-vis spectrum of complex **7** in 2-MeTHF (1.0 x 10^{-4} M) and calculated spectrum (B3LYP-D3(SMD)/6-31G**(SDD)) in THF.

● **Theoretical Analysis of the Molecular Orbitals of Complex 7.**

Table S1. Composition of the frontier orbitals of 7.

MO	eV	Os BzIm	Os iq	Hydrides Os-BzIm	Hydrides Os-iq	Phosphine Os-BzIm	Phosphine Os-iq	carbene	Ph	iq
**L+9**	1.33	78	7	0	2	3	4	0	2	4
**L+8**	0.94	7	72	0	4	1	7	1	4	4
**L+7**	0.75	5	1	0	0	2	1	69	21	1
**L+6**	0.31	6	87	0	0	1	5	1	0	1
**L+5**	0.25	34	3	0	0	3	1	18	17	25
**L+4**	0.21	52	3	0	0	4	1	24	11	4
**L+3**	0.1	4	5	0	0	1	0	30	26	34
**L+2**	-0.4	6	0	0	0	2	0	88	2	2
**L+1**	-0.54	0	3	0	0	0	1	1	1	94
**LUMO**	-1.42	1	4	0	0	0	2	1	16	75
**HOMO**	-4.31	6	34	0	0	1	7	8	35	9
**H-1**	-4.53	36	16	0	0	9	5	2	25	7
**H-2**	-4.92	8	64	0	0	2	12	0	3	10
**H-3**	-4.96	55	5	0	0	14	1	23	2	0
**H-4**	-5.67	12	16	5	7	3	5	3	30	19
**H-5**	-5.77	14	12	3	11	3	3	4	32	18
**H-6**	-5.93	1	14	1	0	1	4	29	32	19
**H-7**	-6.25	11	6	16	8	4	8	2	44	2
**H-8**	-6.27	1	17	1	1	1	67	6	4	2
**H-9**	-6.4	14	1	0	0	26	4	10	25	21

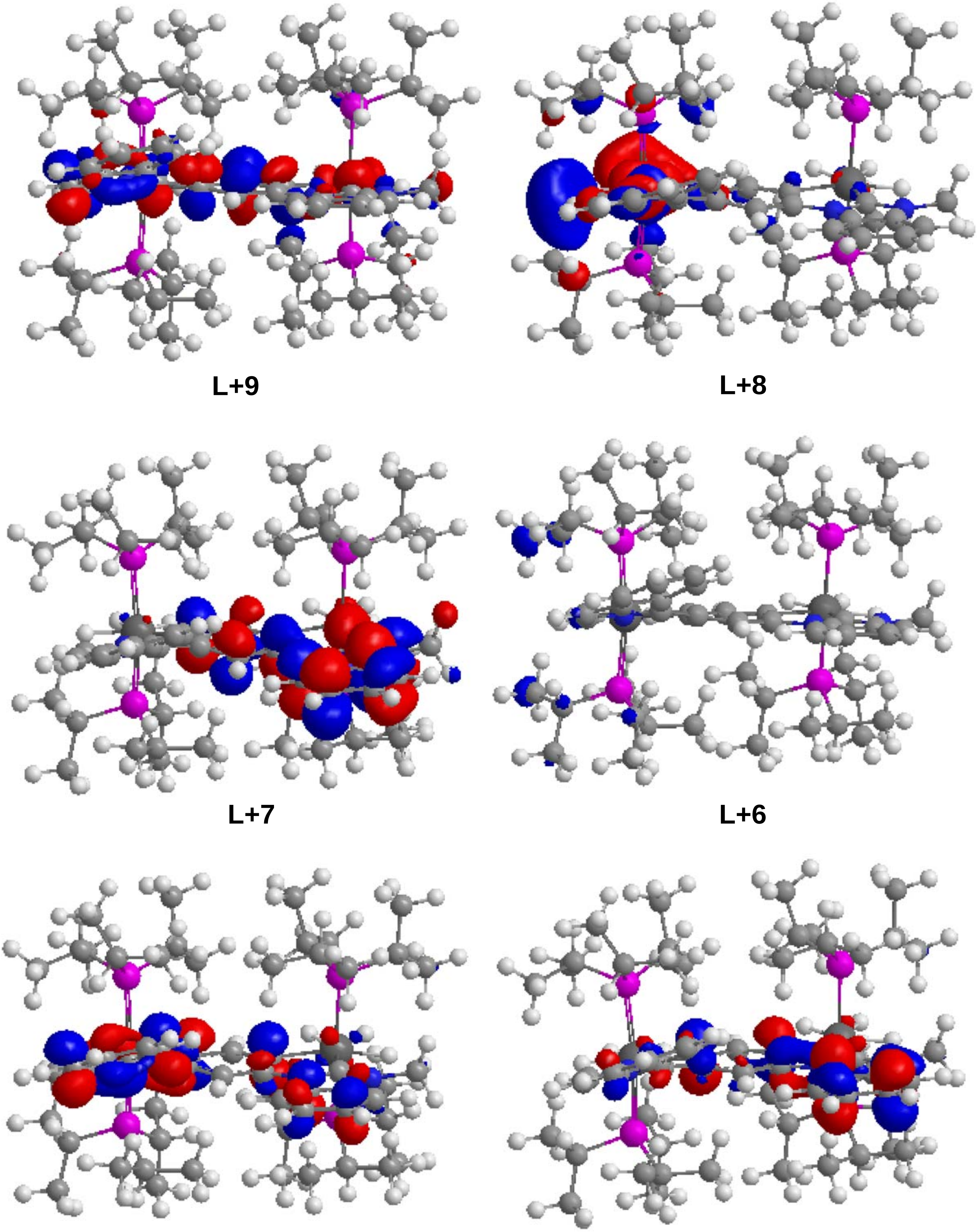

Figure S38. Frontier molecular orbitals of complex **7** (isovalue 0.003 au).

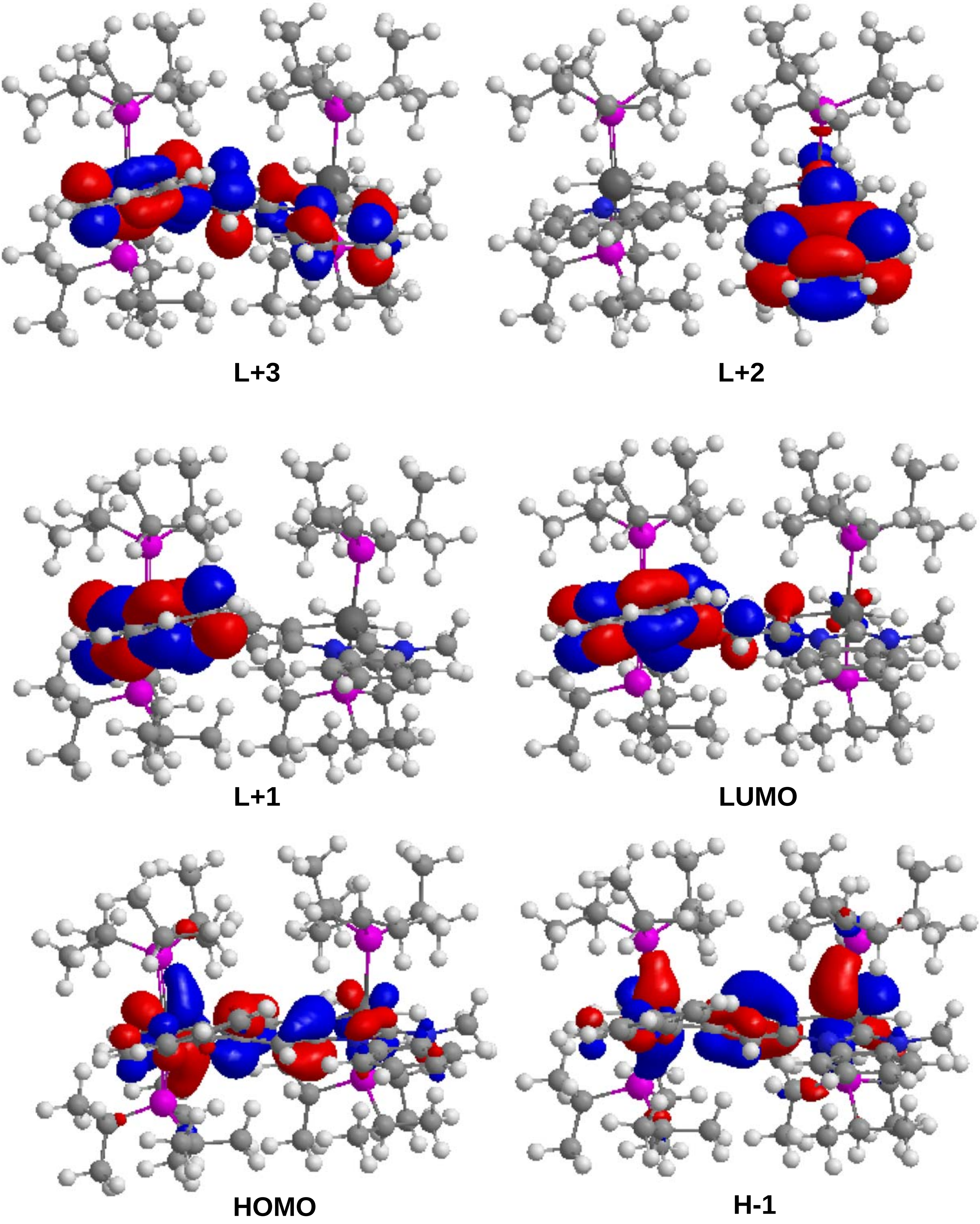

Figure S38. Continued

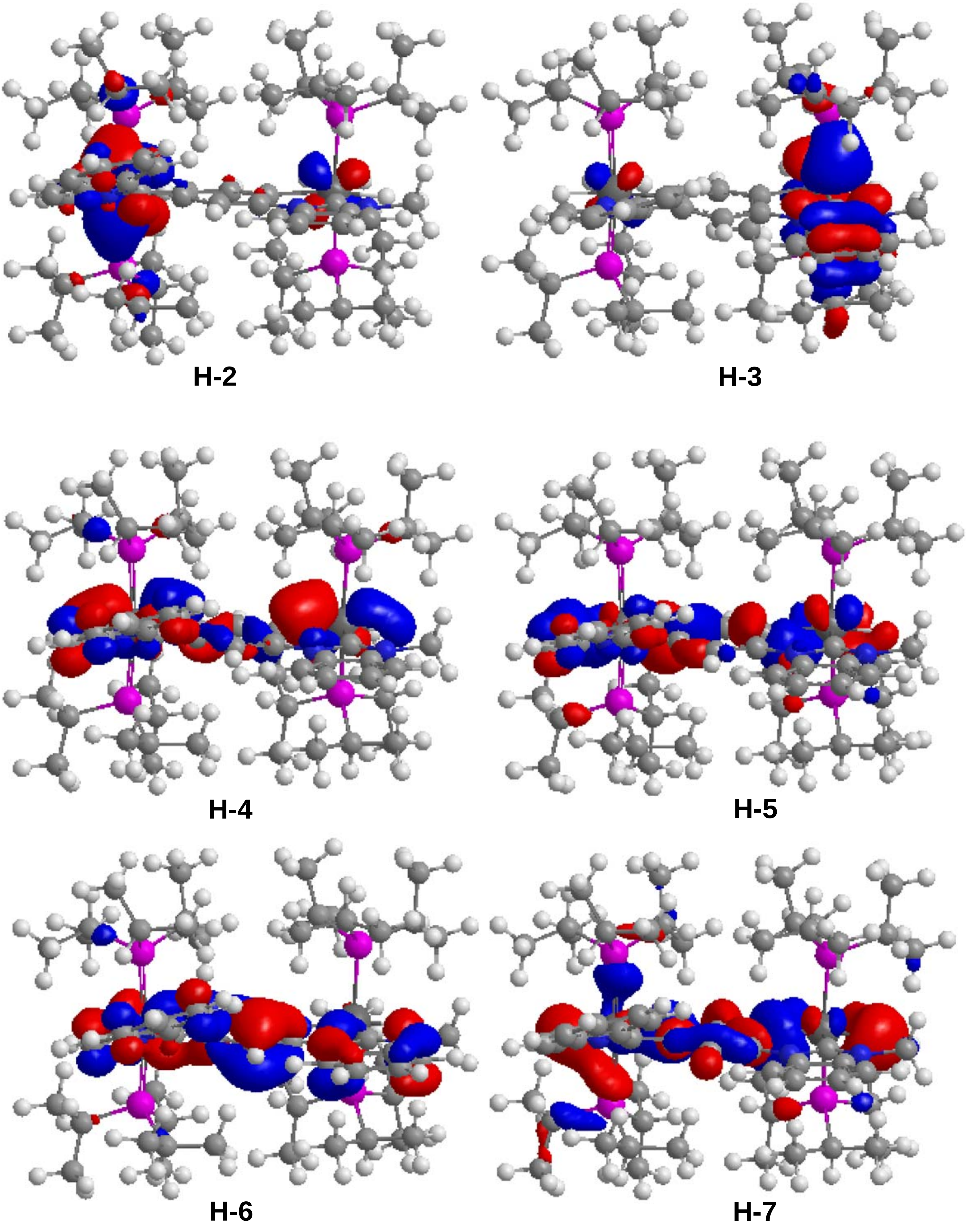

Figure S38. Continued

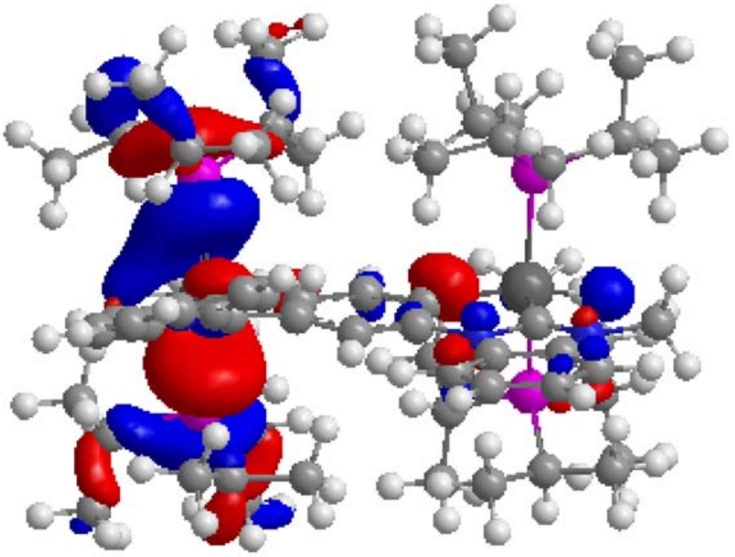

H-8

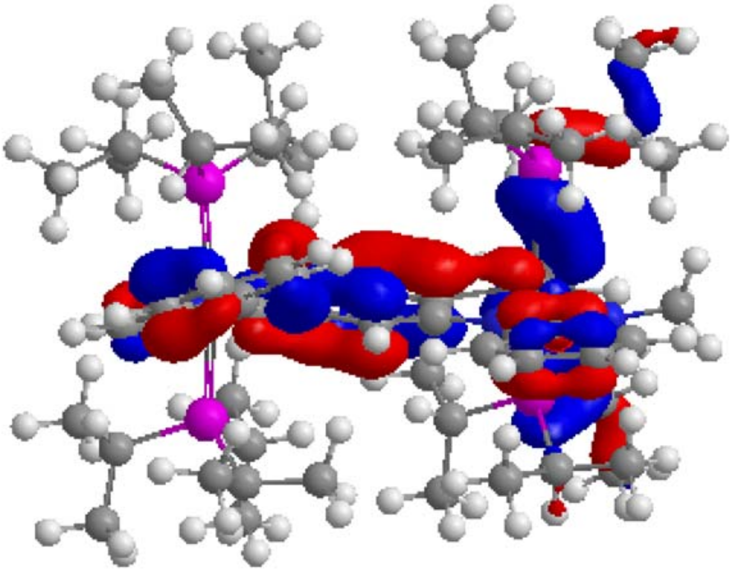

H-9

Figure S38. Continued

• **Cyclic Voltammogram of Complex 7**

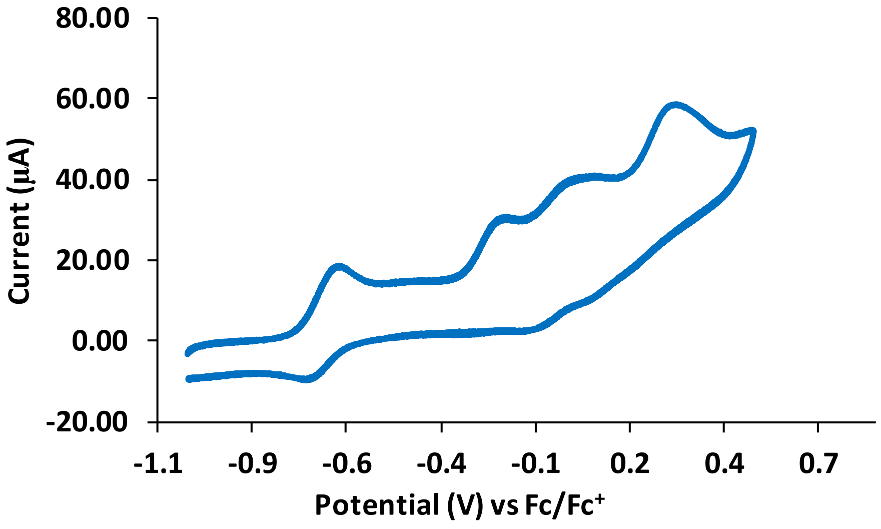

Figure S39. Cyclic voltammogram of complex **7** in dichloromethane 10^{-3} M solution with $[Bu_4N]PF_6$ as supporting electrolyte (0.1 M) at a scan rate of 100 mV s^{-1}. The potentials were referenced to the Fc/Fc^+ couple.

• **Optimized Structures of Complexes 2-7, [7]$^{+}$, [7]$^{2+}$, [7]$^{3+}$, and [7]$^{4+}$**

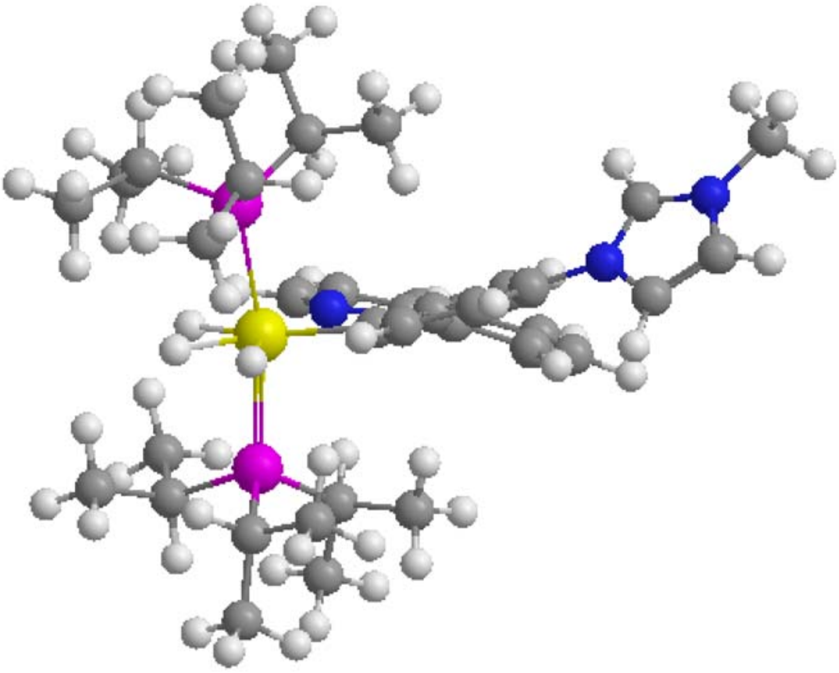

Figure S40. Optimized structure of compound **2**.

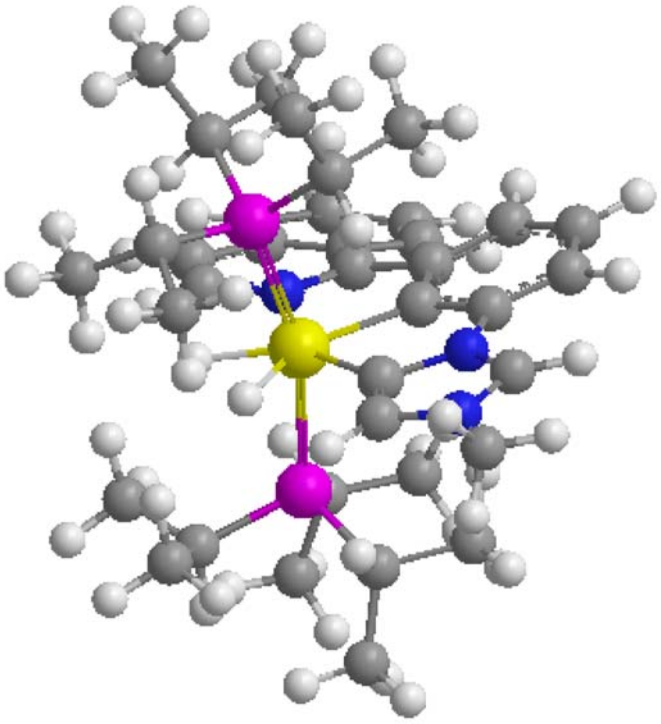

Figure S41. Optimized structure of compound **3**.

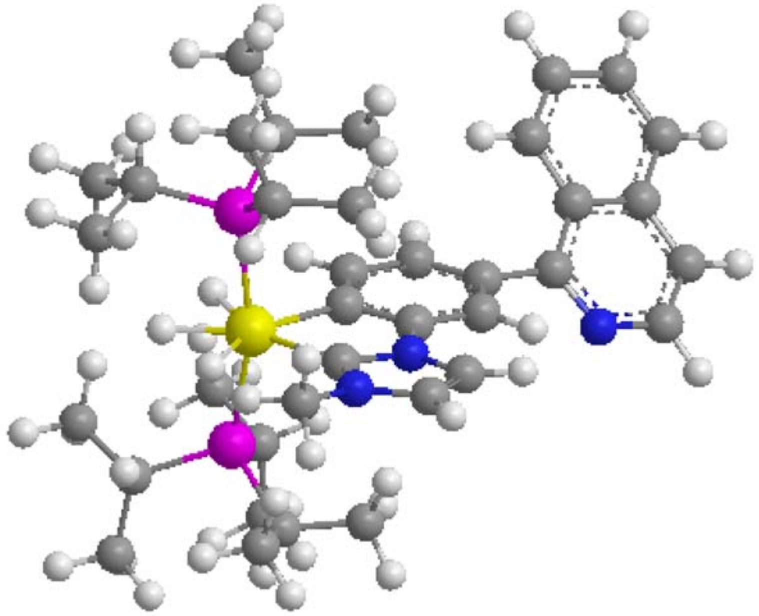

Figure S42. Optimized structure of compound **4.**

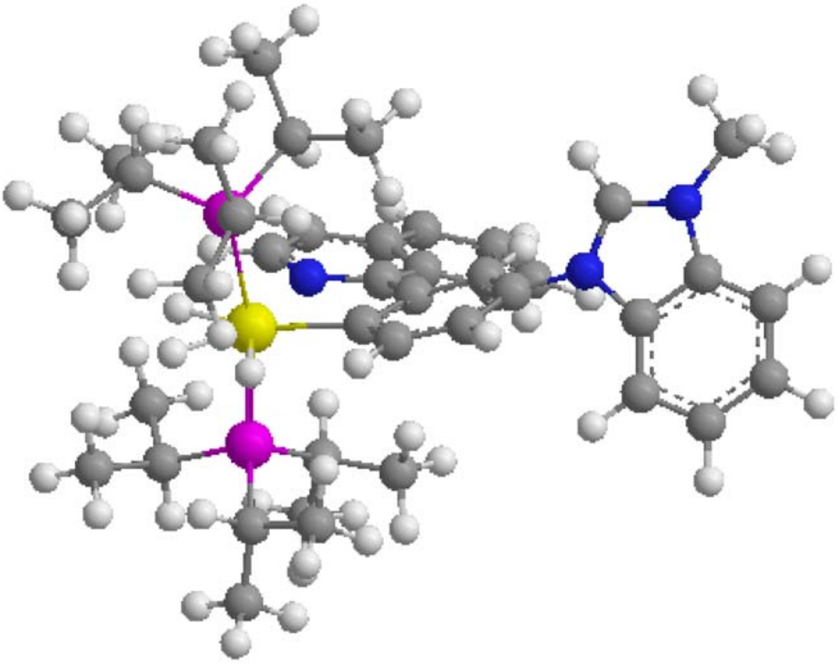

Figure S43. Optimized structure of compound **5.**

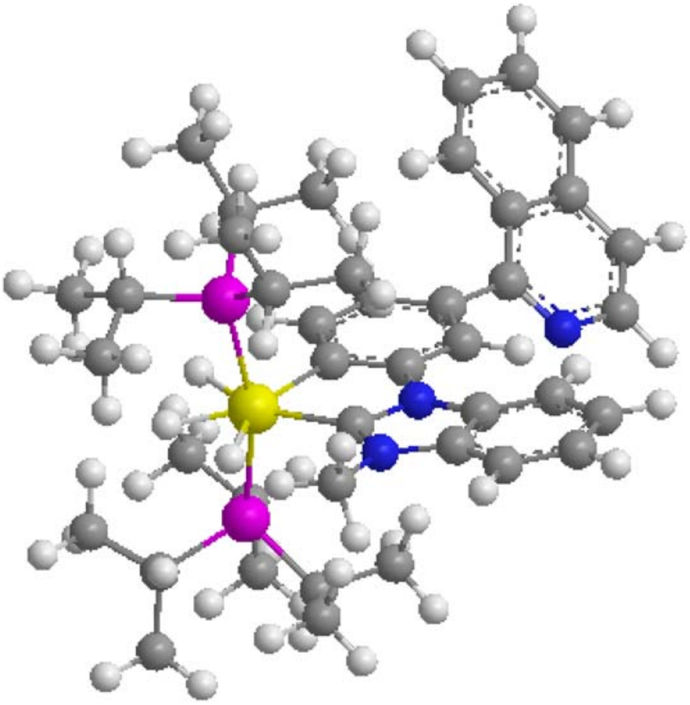

Figure S44. Optimized structure of compound **6.**

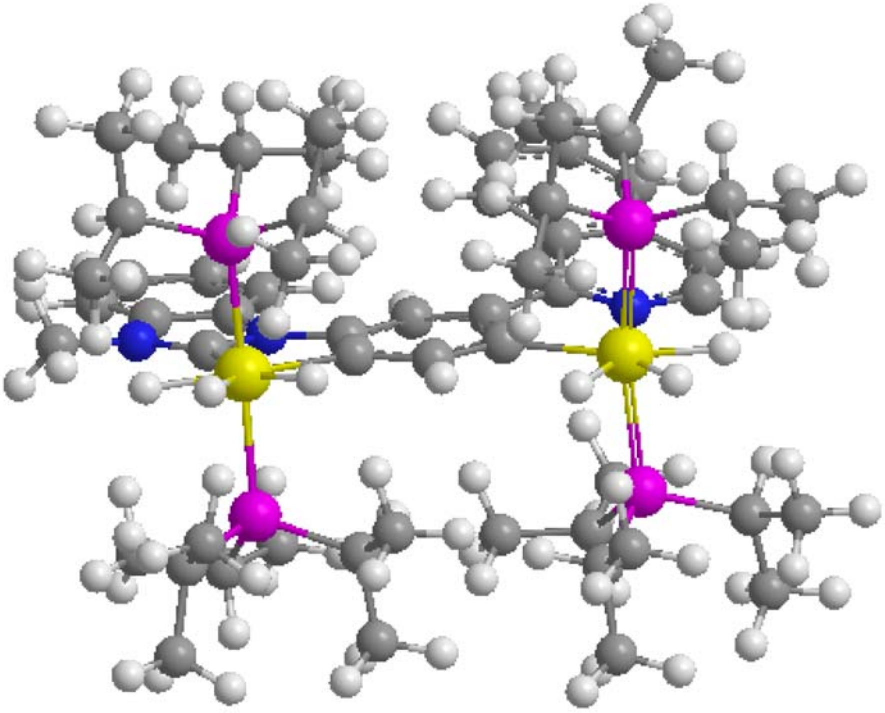

Figure S45. Optimized structure of compound **7**.

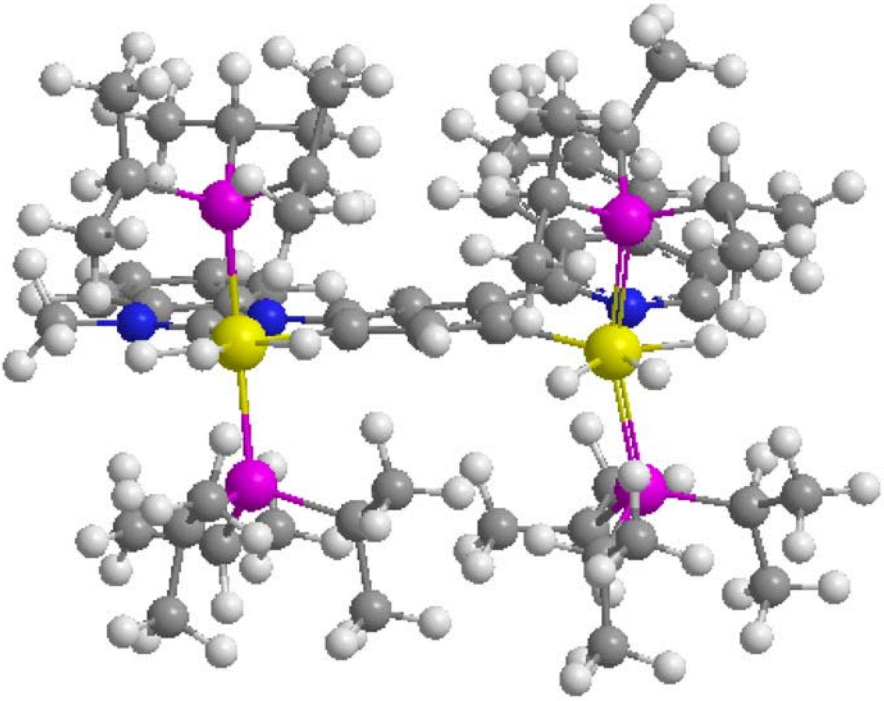

Figure S46. Optimized structure of compound [**7**]$^{+}$.

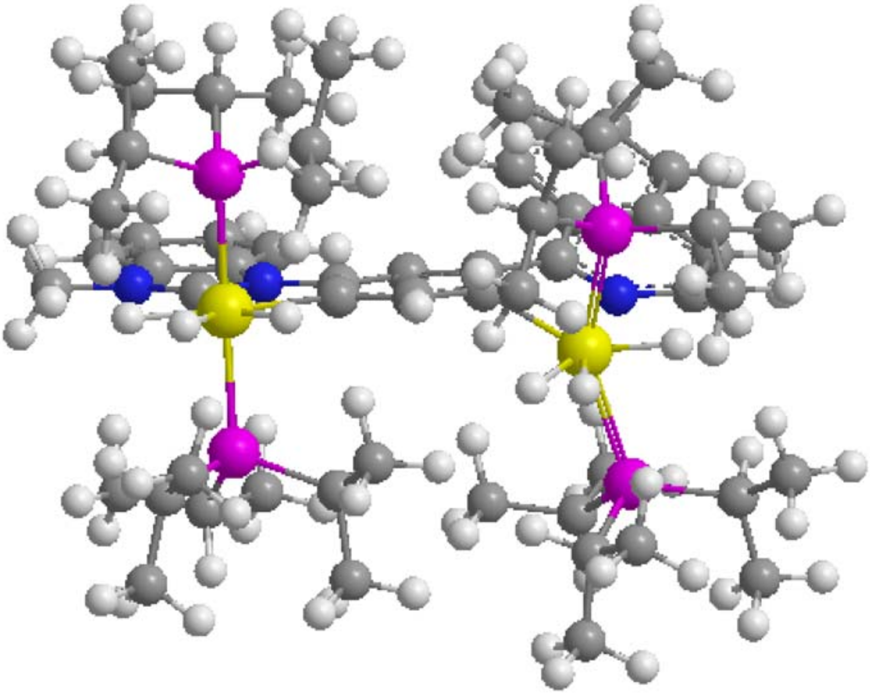

Figure S47. Optimized structure of compound [**7**]$^{2+}$.

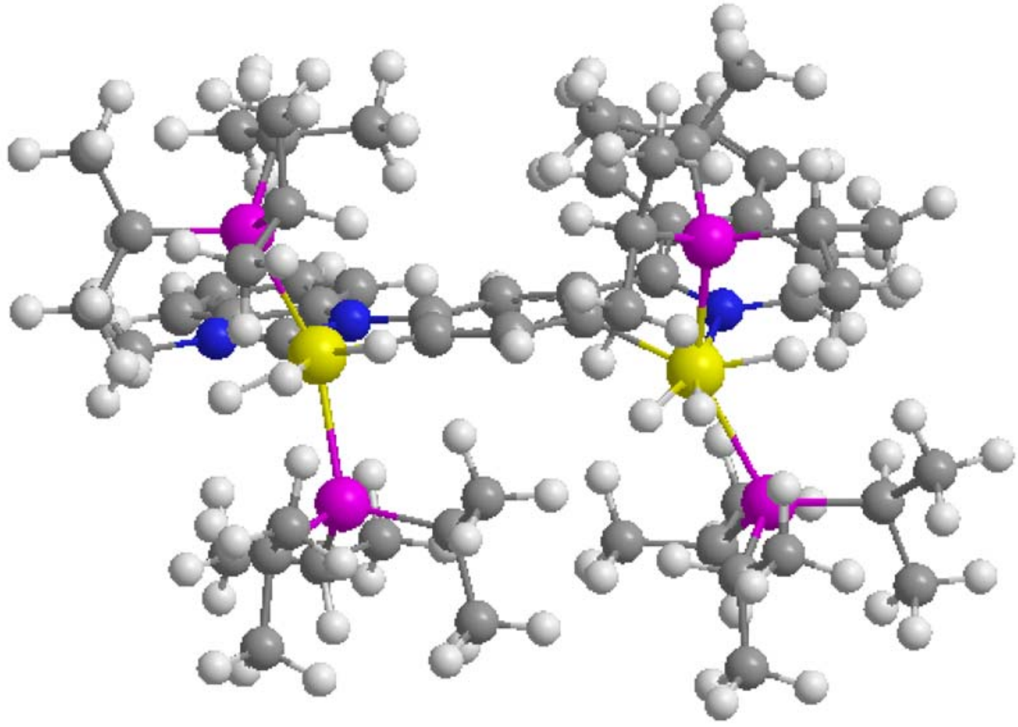

Figure S48. Optimized structure of compound [**7**]$^{3+}$.

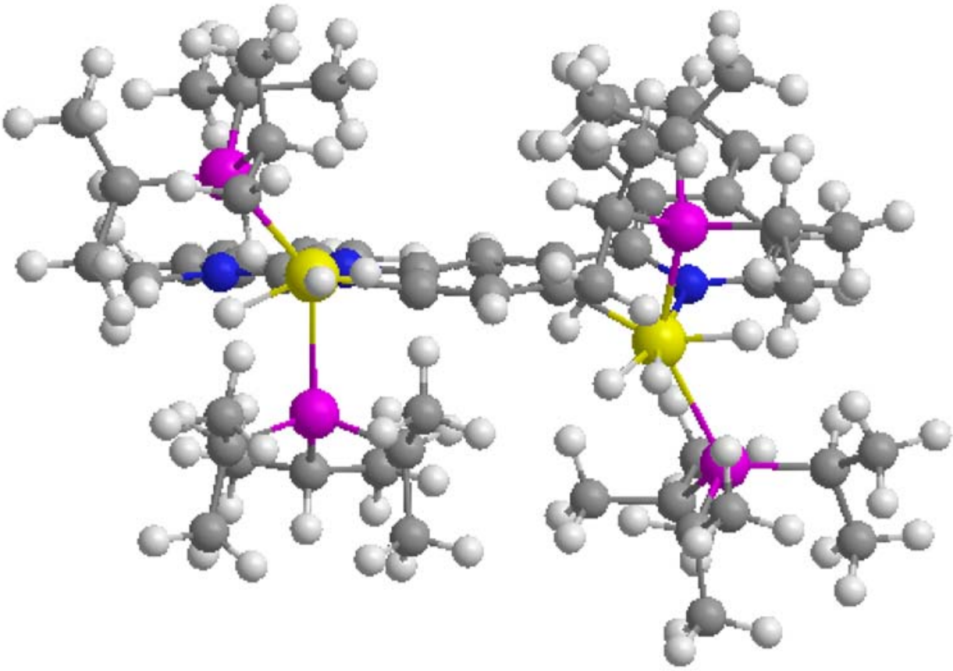

Figure S49. Optimized structure of compound [**7**]$^{4+}$.

● **UV-vis-NIR Spectra of Complex 7**

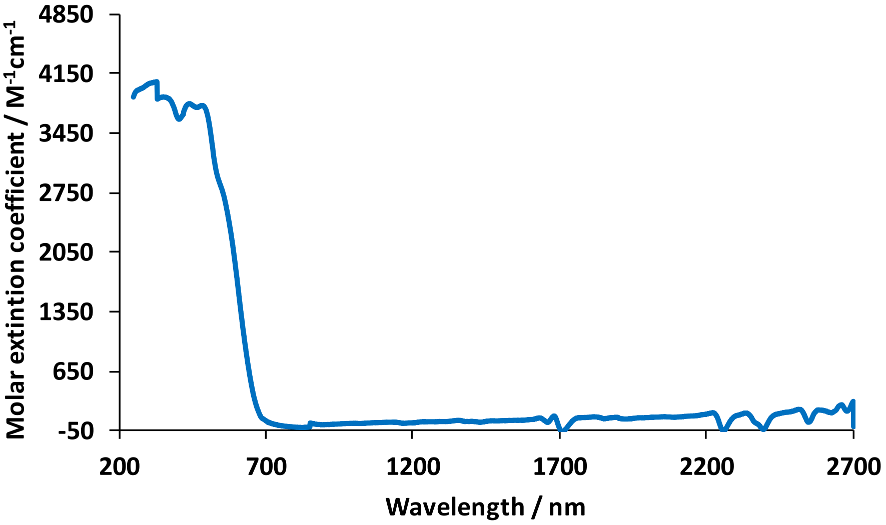

Figure S50. UV-vis-NIR spectrum of complex **7** in CH_2Cl_2.

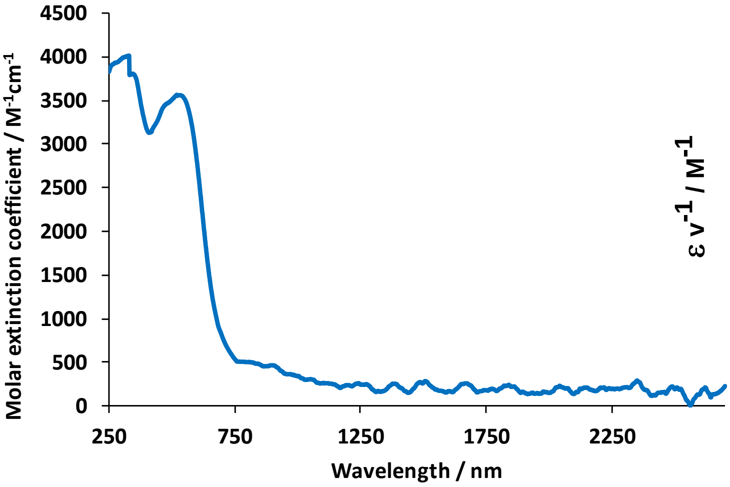

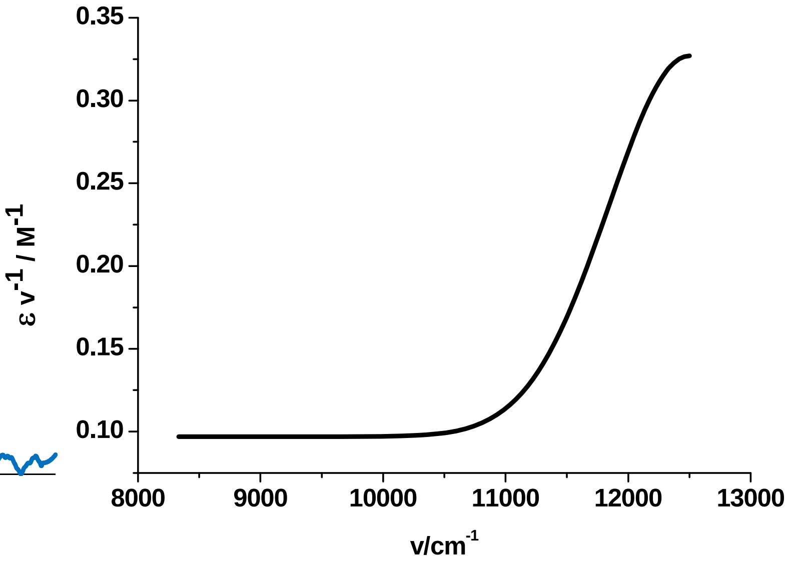

Figure S51. UV-vis-NIR spectrum of complex **[7]**$^+$ in CH_2Cl_2 (left) and $\varepsilon\nu^{-1}$ (M^{-1}) vs ν (cm^{-1}) Gaussian fit (right).

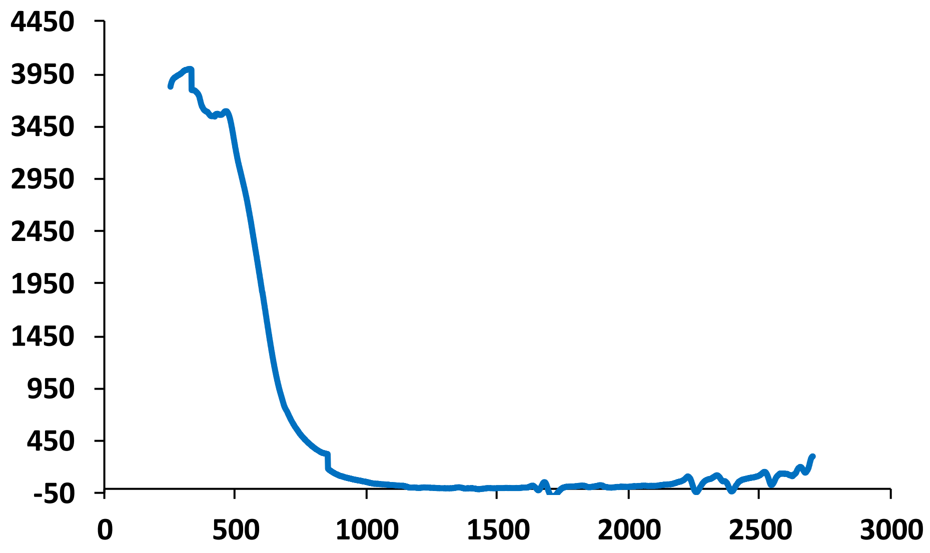

Figure S52. UV-vis-NIR spectrum of complex **[7]**$^{2+}$ in CH_2Cl_2.

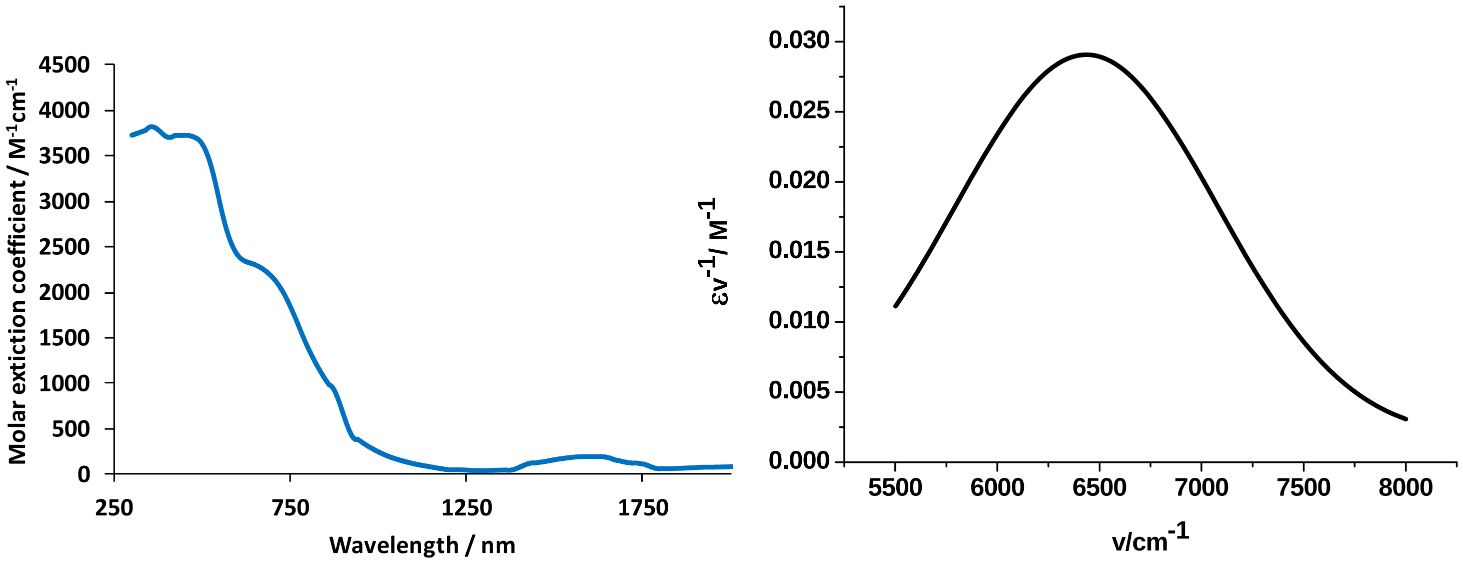

Figure S53. UV-vis-NIR spectrum of complex **[7]**$^{3+}$ in CH_2Cl_2 (left) and εv^{-1} (M^{-1}) vs ν (cm^{-1}) Gaussian fit (right).

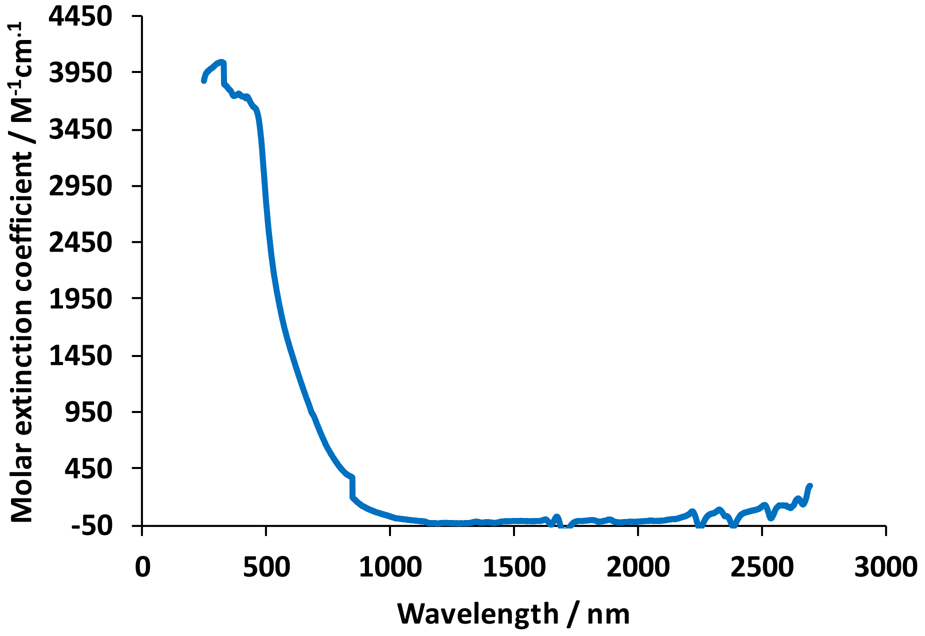

Figure S54. UV-vis-NIR spectrum of complex **[7]**$^{4+}$ in CH_2Cl_2.

● UV-vis-NIR Spectra of Complexes [7]$^{+}$ and [7]$^{3+}$ (Observed and Calculated)

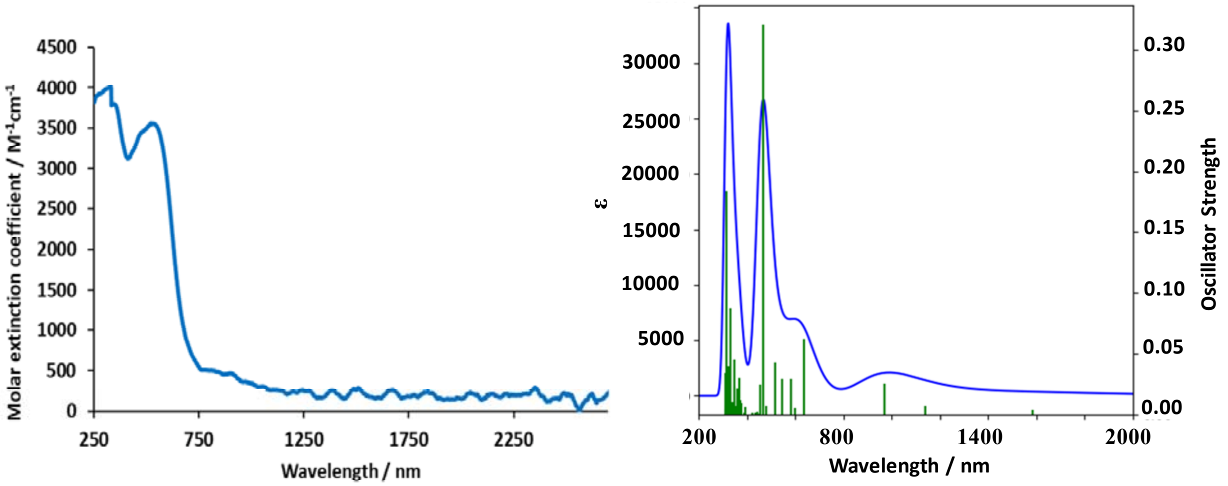

Figure S55. Observed UV-vis-NIR spectrum of complex **[7]$^{+}$** in CH_2Cl_2 (1.0 x 10^{-3} M) and calculated spectrum (B3LYP-D3(SMD)/6-31G**(SDD)) in CH_2Cl_2.

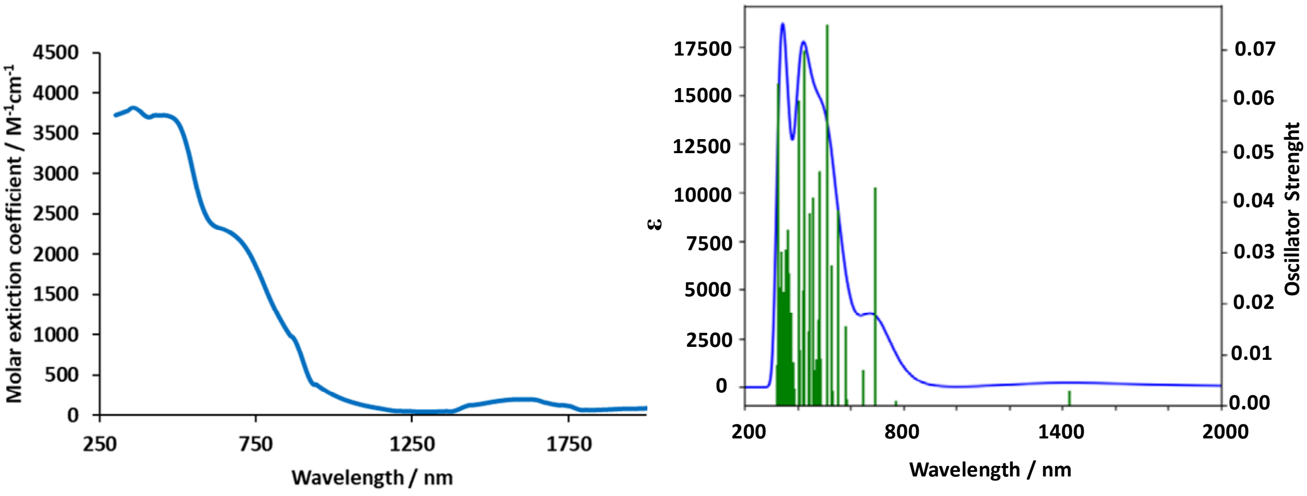

Figure S56. Observed UV-vis-NIR spectrum of complex **[7]$^{3+}$** in CH_2Cl_2 (1.0 x 10^{-3} M) and calculated spectrum (B3LYP-D3(SMD)/6-31G**(SDD)) in CH_2Cl_2.

● **Calculated HOMO, SOMO and LUMO of complexes 7, $[7]^{+}$, $[7]^{2+}$, $[7]^{3+}$ and $[7]^{4+}$**

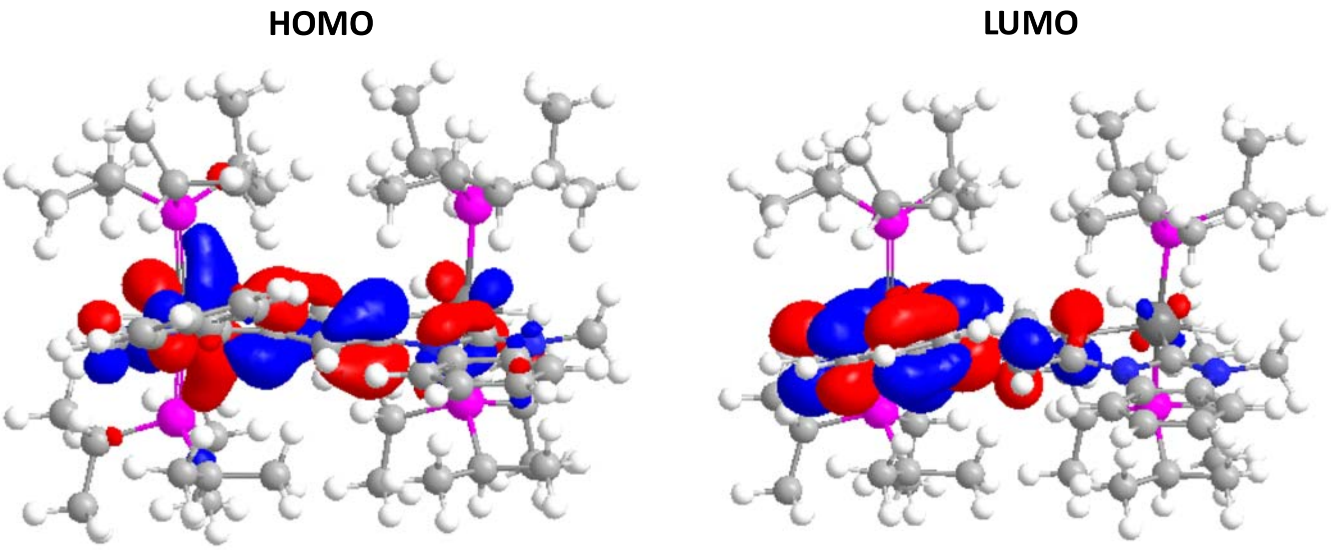

Figure S57. HOMO and LUMO of complex **7**.

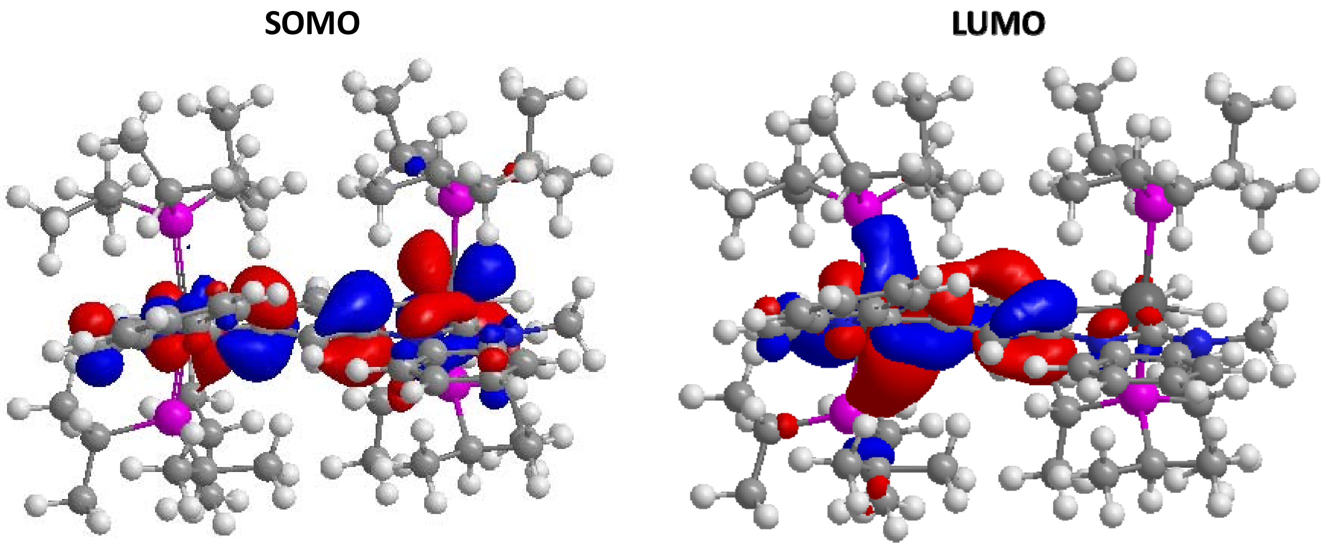

Figure S58. SOMO and LUMO of complex $[\mathbf{7}]^{+}$.

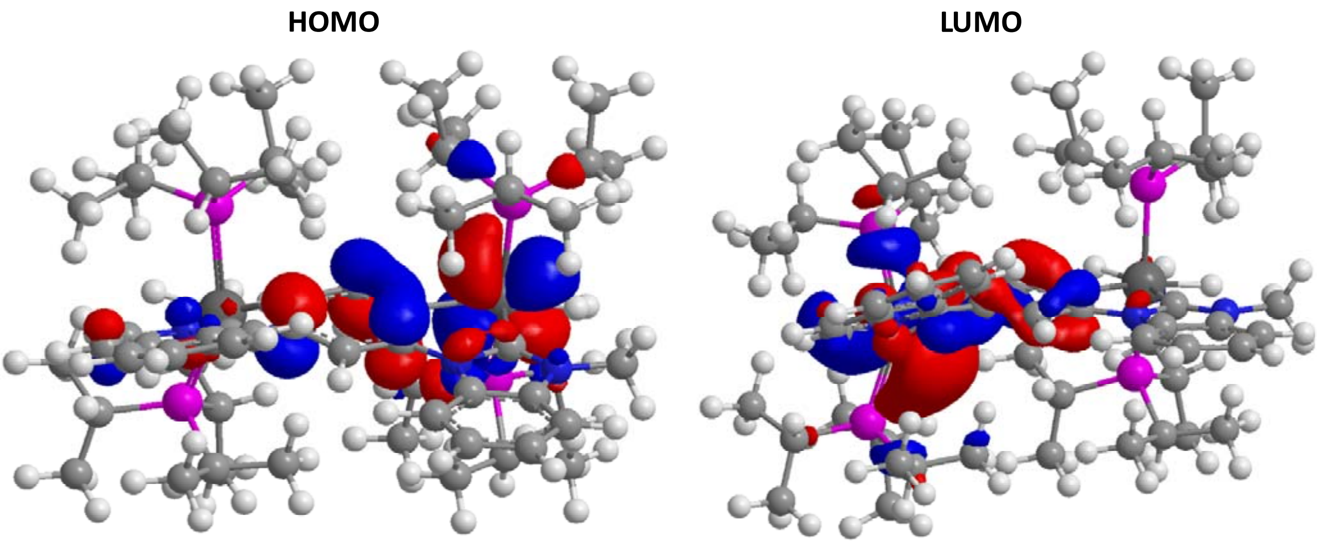

Figure S59. HOMO and LUMO of complex **[7]**$^{2+}$.

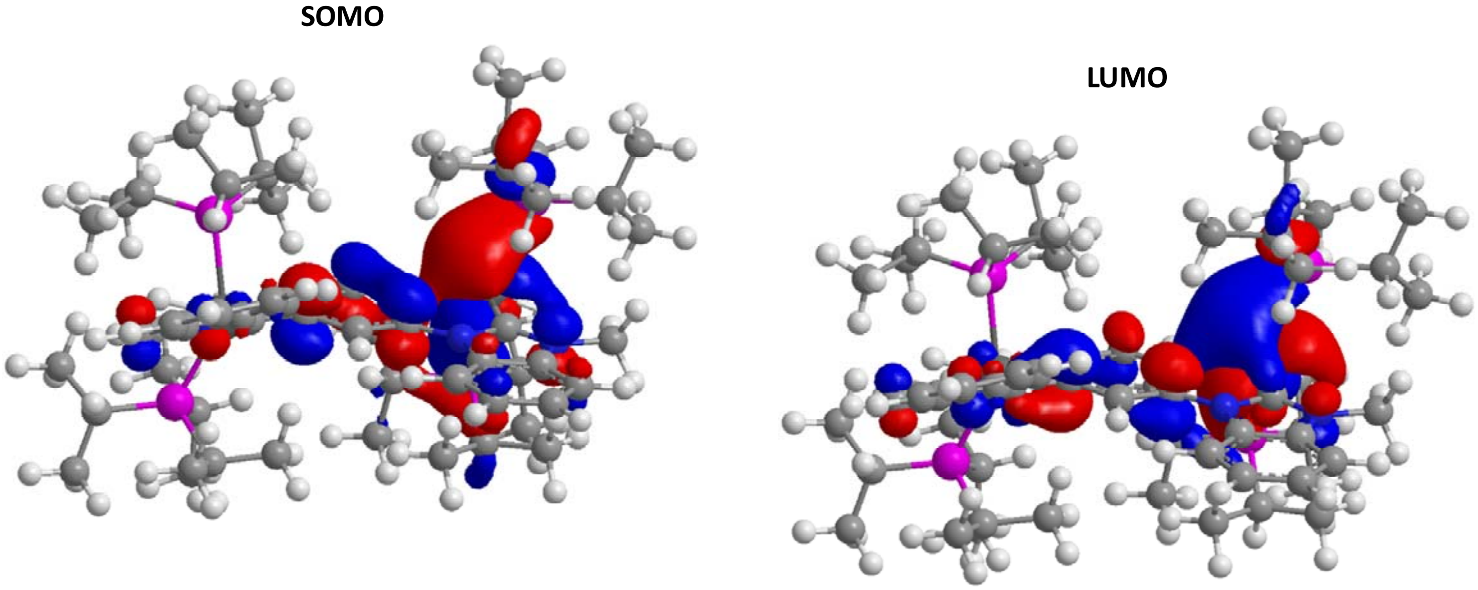

Figure S60. SOMO and LUMO of complex **[7]**$^{3+}$**.**

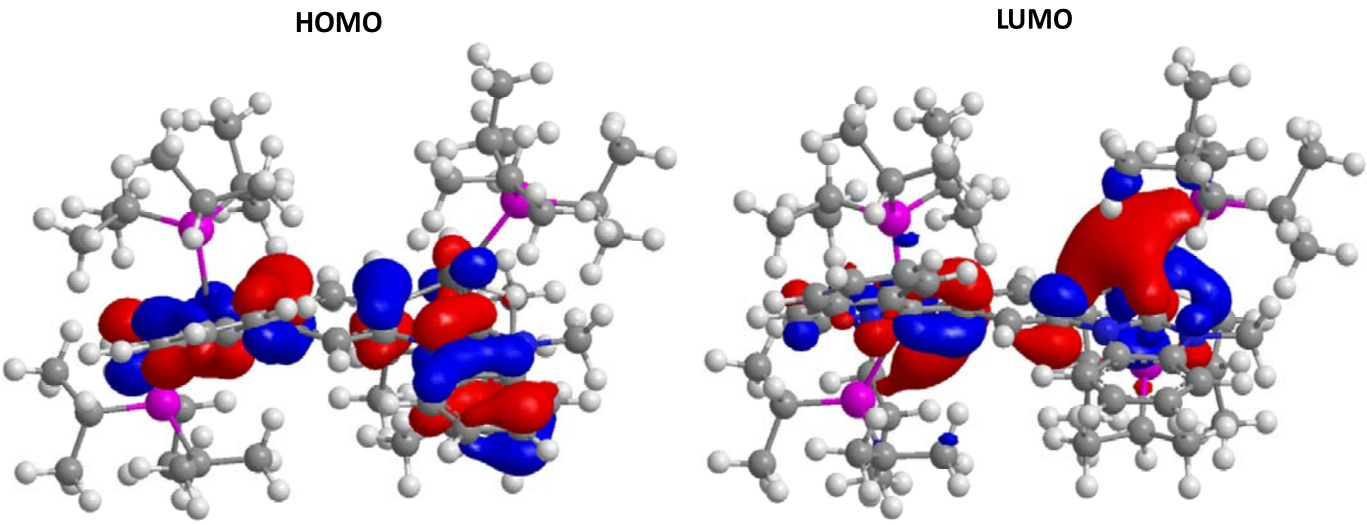

Figure S61. HOMO and LUMO of complex [**7**]$^{4+}$.

● **References**

(1) Aracama, M.; Esteruelas, M. A.; Lahoz, F. J.; Lopez, J. A.; Meyer, U.; A. Oro, L.; Werner, H. Synthesis, Reactivity, Molecular Structure, and Catalytic Activity of the Novel Dichlorodihydridoosmium(IV) Complexes $OsH_2Cl_2(PR_3)_2$ (PR_3 = P^iPr_3, PMe^tBu_2). *Inorg. Chem.* **1991**, *30*, 288-293.

(2) Blessing, R. H. *Acta Crystallogr.* **1995**, *A51*, 33. SADABS: Area-detector absorption correction; Bruker- AXS, Madison, WI, 1996.

(3) SHELXL-2016/6. Sheldrick, G. M. *Acta Cryst.* **2008**, *A64*, 112-122.

(4) (a) Lee, C.; Yang, W.; Parr, R. G. Development of the Colle-Salvetti correlation-energy formula into a functional of the electron density. *Phys. Rev. B* **1988**, *37*, 785-789. (b) Becke, A. D. Density-functional exchange-energy approximation with correct asymptotic behavior. *J. Chem. Phys.* **1993**, *98*, 5648-5652. (c) Stephens, P. J.; Devlin, F. J.; Chabalowski, C. F.; Frisch, M. J. Ab Initio Calculation of Vibrational Absorption and Circular Dichroism Spectra Using Density Functional Force Fields. *J. Phys. Chem.* **1994**, *98*, 11623-11627.

(5) Grimme, S.; Antony, J.; Ehrlich, S.; Krieg, H. A consistent and accurate ab initio parametrization of density functional dispersion correction (DFT-D) for the 94 elements H-Pu. *J. Chem. Phys.* **2010**, *132*, 154104.

(6) Gaussian 09, Revision D.01, Frisch, M. J.; Trucks, G. W.; Schlegel H. B.; Scuseria, G. E.; Robb, M. A.; Cheeseman, J. R.; Scalmani, G.; Barone, V.; Mennucci, B.; Petersson, G. A.; Nakatsuji, H.; Caricato, M.; Li, X.; Hratchian, H. P.; Izmaylov, A. F.; Bloino, J.; Zheng, G.; Sonnenberg, J. L.; Hada, M.; Ehara, M.; Toyota, K.; Fukuda, R.; Hasegawa, J.; Ishida, M.; Nakajima, T.; Honda, Y.; Kitao, O.; Nakai, H.; Vreven, T.; Montgomery, J. A.; Peralta, Jr., J. E.; Ogliaro, F.; Bearpark, M.; Heyd, J. J.; Brothers, E.; Kudin, K. N.; Staroverov, V. N.; Keith, T.; Kobayashi, R.; Normand, J.; Raghavachari, K.; Rendell, A.; Burant, J. C.; Iyengar, S. S.; Tomasi, J.; Cossi, M.; Rega, N.; Millam, J. M.; Klene, M.; Knox, J. E.; Cross, J. B.; Bakken, V.; Adamo, C.; Jaramillo, J.; Gomperts, R.; Stratmann, R. E.; Yazyev, O.; Austin, A. J.; Cammi, R.; Pomelli, C.; Ochterski, J. W.; Martin, R. L.; Morokuma, K.; Zakrzewski, V. G.; Voth, G. A.; Salvador, P.; Dannenberg, J. J.; Dapprich, S.; Daniels, A. D.; Farkas, O.; Foresman, J. B.; Ortiz, J. V.; Cioslowski, J.; Fox, D. J. Gaussian, Inc., Wallingford CT, 2013.

(7) Andrea, D.; Häußermann, U. M.; Dolg, M.; Stoll, H.; Preuss, H. Energy-adjusted ab initio pseudopotentials for the second and third row transition elements. *Theor. Chim. Acta* **1990**, *77*, 123-141.

(8) Ehlers, A. W.; Bohme, M.; Dapprich, S.; Gobbi, A.; Hollwarth, A.; Jonas, V.; Kohler, K. F.; Stegmann, R.; Veldkamp, A.; Frenking, G. A set of f-polarization functions for pseudo-potential basis sets of the transition metals SC-Cu, Y-Ag and La-Au. *Chem. Phys. Lett.* **1993**, *208*, 111-114.

(9) (a) Hehre, W. J.; Ditchfield, R.; Pople, J. A. Self-Consistent Molecular Orbital Methods. XII. Further Extensions of Gaussian-Type Basis Sets for Use in Molecular Orbital Studies of Organic Molecules. *J. Chem. Phys.* **1972**, *56*, 2257-2261. (b) Francl,

M. M.; Pietro, W. J.; Hehre, W. J.; Binkley, J. S.; Gordon, M. S.; DeFrees, D. J.; Pople, J. A. Self-consistent molecular orbital methods. XXIII. A polarization-type basis set for second-row elements. *J. Chem. Phys.* **1982**, *77*, 3654-3665.

(10) Marenich, A. V.; Cramer, C. J.; Truhlar, D. G. Universal Solvation Model Based on Solute Electron Density and on a Continuum Model of the Solvent Defined by the Bulk Dielectric Constant and Atomic Surface Tensions. *J. Phys. Chem. B* **2009**, *113*, 6378-6396.

(11) O'Boyle, N. M.; Tenderholt, A. L.; Langner, K. M. cclib: A Library for Package-Independent Computational Chemistry Algorithms. *J. Comput. Chem.* **2008**, *29*, 839-845.
